# Supplementary material for: Iron-deplete diet enhances Caenorhabditis elegans lifespan via oxidative stress response pathways
Source: EMBO J. 2025 Nov 10;44(24):7565–89. doi: 10.1038/s44318-025-00634-7 (PMC12706066; doi:10.1038/s44318-025-00634-7)
Supplement: Supplementary file 1 — Appendix [file 44318_2025_634_MOESM1_ESM.pdf]

Appendix

Iron-deplete diet enhances *Caenorhabditis elegans* lifespan via oxidative stress response pathways

Priyanka Das[1], Ravi[1], and Jogender Singh[1,*]

[1]Department of Biological Sciences, Indian Institute of Science Education and Research, Mohali, Punjab, 140306, India

[*]For correspondence: jogender@iisermohali.ac.in

Table of contents

Appendix Figure S1 2

Appendix Figure S2 2

Appendix Figure S3 3

Appendix Figure S4 4

Appendix Figure S5 5

Appendix References.. 6

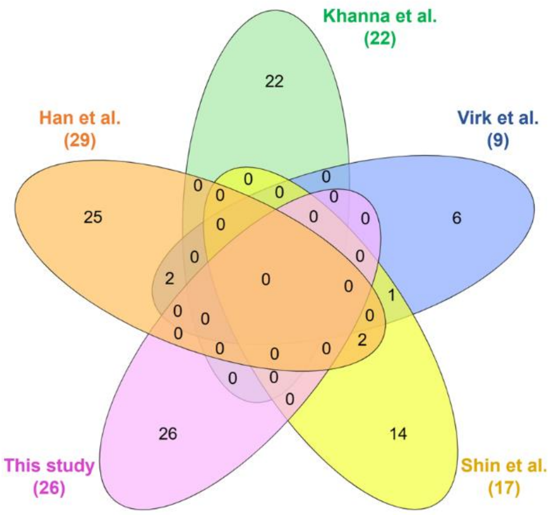

Appendix Figure S1. The pro-longevity *E. coli* mutants identified in this study differ from previously identified mutants

Venn diagram showing the overlap among *E. coli* mutants identified in previous genome-wide bacterial screens for mutants that extend *C. elegans* lifespan (Han *et al*, 2017; Khanna *et al*, 2016; Shin *et al*, 2020; Virk *et al*, 2016) and the mutants identified in this study.

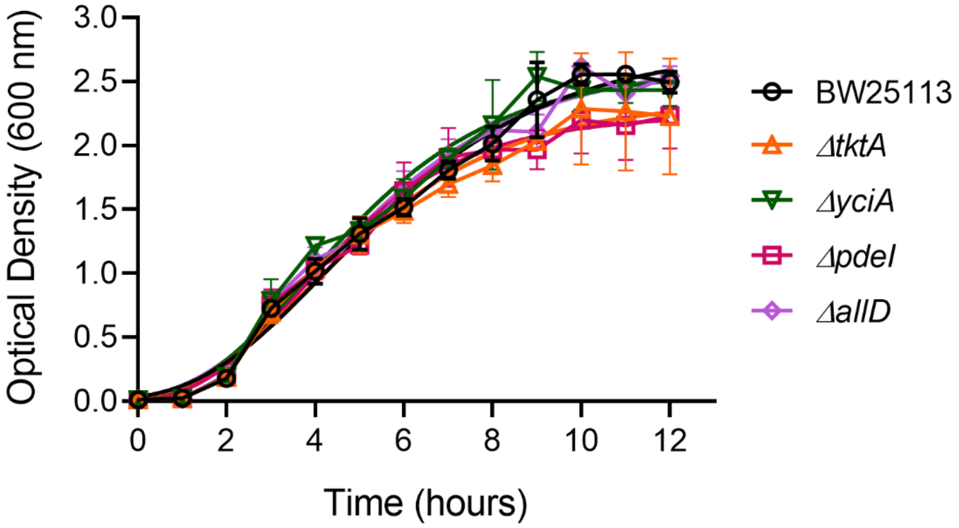

Appendix Figure S2. *E. coli* mutants do not exhibit growth defects

Growth curves of different *E. coli* strains in Luria-Bertani (LB) broth at 37°C. Data represent the mean and standard deviation from three independent experiments.

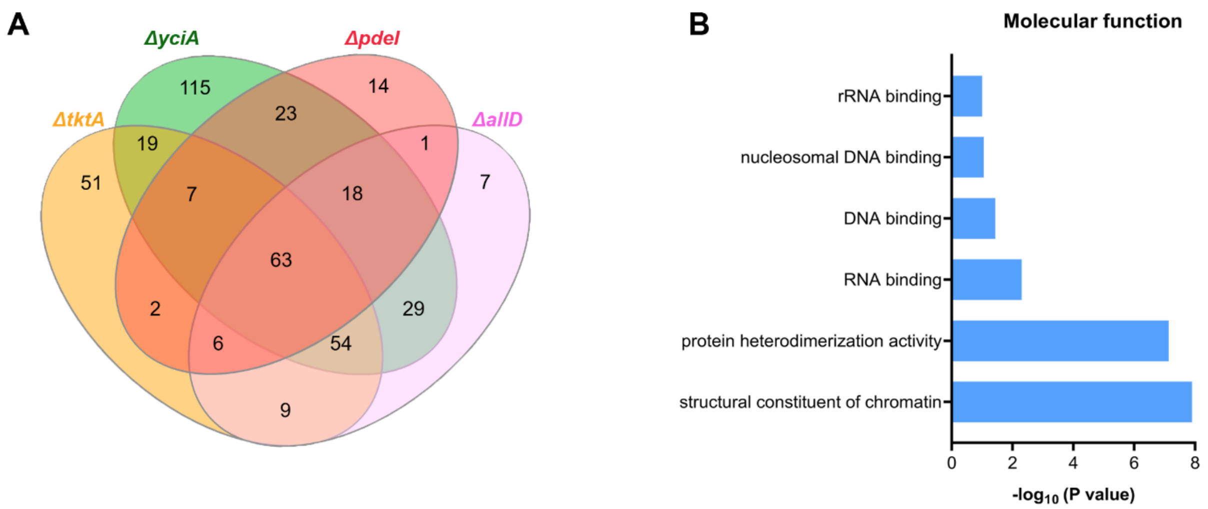

Appendix Figure S3. Different *E. coli* mutants induce similar transcriptomic changes in *C. elegans*

(A) Venn diagram showing the overlap among genes downregulated in N2 worms grown on *E. coli* mutants *ΔtktA*, *ΔyciA*, *ΔpdeI*, and *ΔallD* compared to the BW25113 control.

(B) Gene ontology enrichment analysis of molecular function for the common 63 genes downregulated in N2 worms grown on *E. coli* mutants *ΔtktA*, *ΔyciA*, *ΔpdeI*, and *ΔallD*. The statistical analysis was performed using Fisher's exact test.

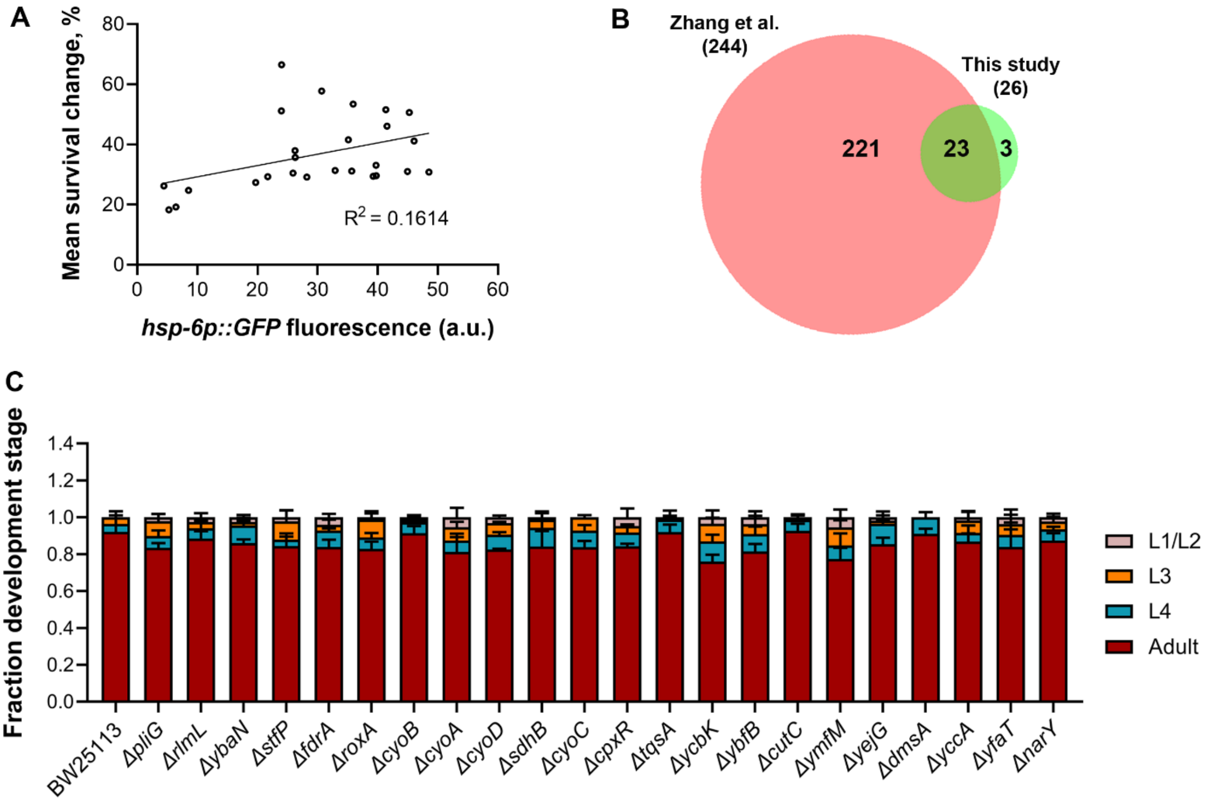

Appendix Figure S4. Iron supplementation rescues mutant diet phenotypes

(A) Correlation between *hsp-6p::GFP* fluorescence levels and the percent mean survival change in lifespan in wild-type N2 worms fed on different FAT-7-suppressing diets.

(B) Venn diagram showing the overlap between *E. coli* mutants identified by (Zhang *et al*, 2019) and those identified in this study. The Venn diagram was obtained using the web tool BioVenn (https://www.biovenn.nl/).

(C) Quantification of different developmental stages of N2 worms grown at 20°C for 60 hours after transferring synchronized L1 larvae onto *E. coli* BW25113 and mutant diets supplemented with 4 mM ferric chloride (n = 3 biological replicates; animals per condition per replicate >60). Data represent the mean and standard deviation from three independent experiments.

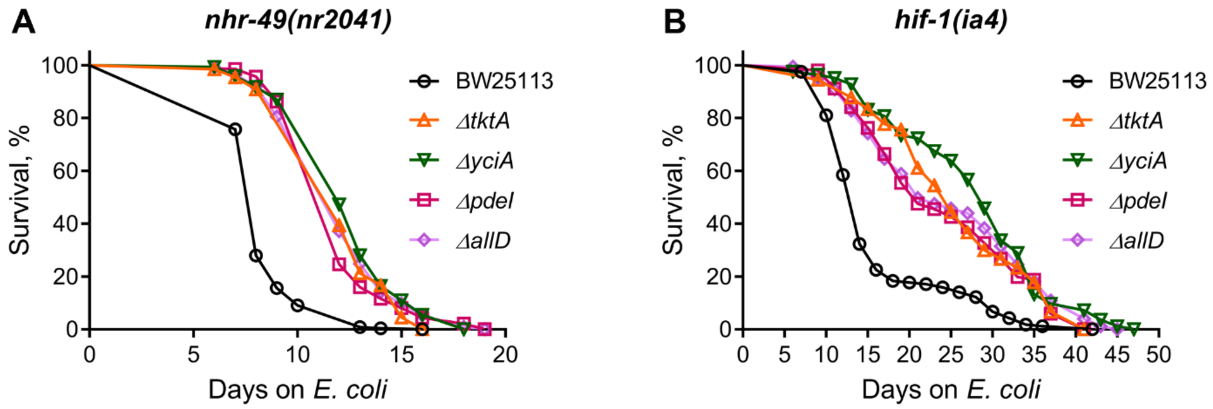

Appendix Figure S5. HIF-1 and NHR-49 are not required for lifespan extension on mutant diets

(A) Representative survival curves of *nhr-49(nr2041)* worms fed on *ΔtktA*, *ΔyciA*, *ΔpdeI*, and *ΔallD E. coli* mutants along with the BW25113 control. $p < 0.001$ for all the mutant diets compared to the BW25113 control (n = 3 biological replicates; animals per condition per replicate > 60).

(B) Representative survival curves of *hif-1(ia4)* worms fed on *ΔtktA*, *ΔyciA*, *ΔpdeI*, and *ΔallD E. coli* mutants along with the BW25113 control. $p < 0.001$ for all the mutant diets compared to the BW25113 control (n = 3 biological replicates; animals per condition per replicate > 80).

Appendix References:

Han B, Sivaramakrishnan P, Lin C-CJ, Neve IAA, He J, Tay LWR, Sowa JN, Sizovs A, Du G, Wang J, *et al* (2017) Microbial Genetic Composition Tunes Host Longevity. *Cell* 169: 1249-1262.e13

Khanna A, Kumar J, Vargas MA, Barrett L, Katewa S, Li P, McCloskey T, Sharma A, Naudé N, Nelson C, *et al* (2016) A genome-wide screen of bacterial mutants that enhance dauer formation in C. elegans. *Sci Rep* 6: 38764

Shin M-G, Lee J-W, Han J-S, Lee B, Jeong J-H, Park S-H, Kim J-H, Jang S, Park M, Kim S-Y, *et al* (2020) Bacteria-derived metabolite, methylglyoxal, modulates the longevity of C. elegans through TORC2/SGK-1/DAF-16 signaling. *Proc Natl Acad Sci* 117: 17142–17150

Virk B, Jia J, Maynard CA, Raimundo A, Lefebvre J, Richards SA, Chetina N, Liang Y, Helliwell N, Cipinska M, *et al* (2016) Folate Acts in *E. coli* to Accelerate *C. elegans* Aging Independently of Bacterial Biosynthesis. *Cell Rep* 14: 1611–1620

Zhang J, Li X, Olmedo M, Holdorf AD, Shang Y, Artal-Sanz M, Yilmaz LS & Walhout AJM (2019) A Delicate Balance between Bacterial Iron and Reactive Oxygen Species Supports Optimal C. elegans Development. *Cell Host Microbe* 26: 400-411.e3
