# Supplementary figures and images for: Iron-deplete diet enhances Caenorhabditis elegans lifespan via oxidative stress response pathways (part 1 of 2)
Source: EMBO J. 2025 Nov 10;44(24):7565–89. doi: 10.1038/s44318-025-00634-7 (PMC12706066; doi:10.1038/s44318-025-00634-7)

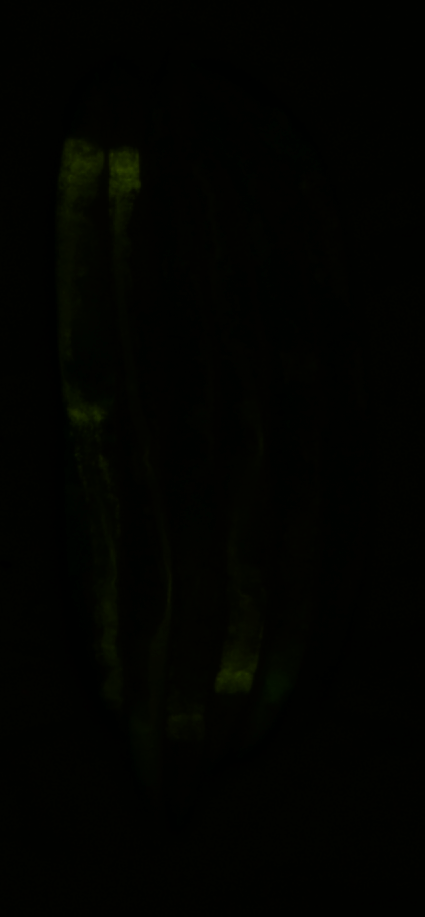

Supplement: Supplementary file 9 — Source data Fig. 3 [file 44318_2025_634_MOESM9_ESM.zip › Figure 3/Source data_Figure 3G/BW25113.tif]

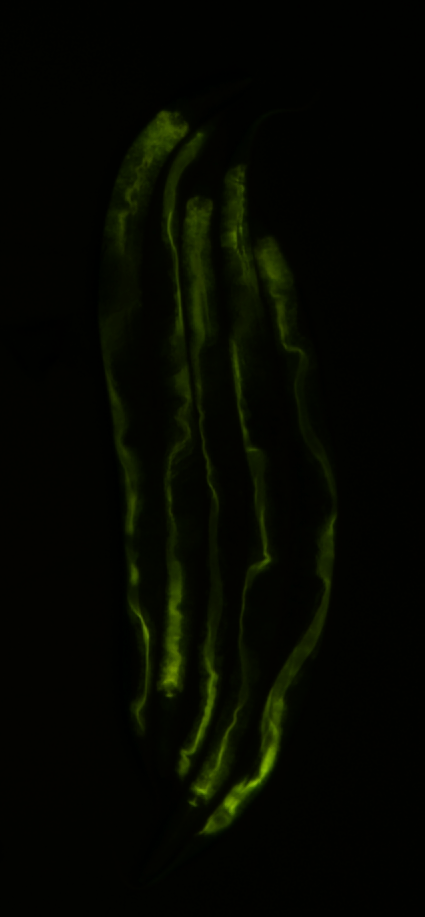

Supplement: Supplementary file 9 — Source data Fig. 3 [file 44318_2025_634_MOESM9_ESM.zip › Figure 3/Source data_Figure 3G/ΔallD.tif]

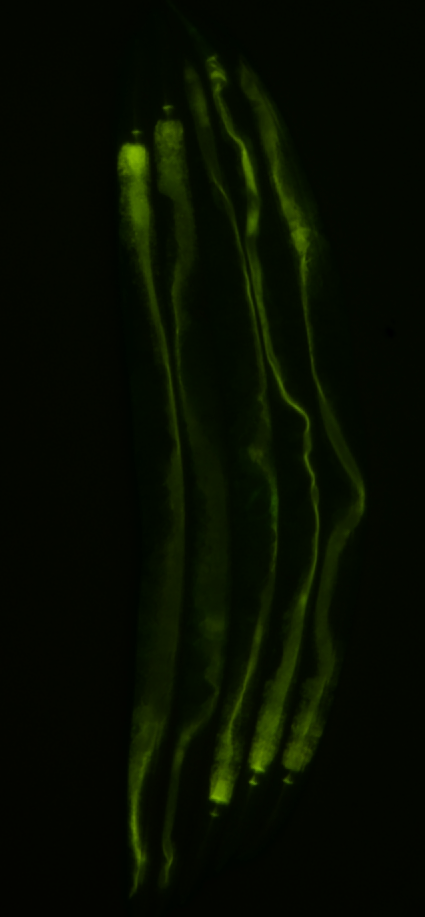

Supplement: Supplementary file 9 — Source data Fig. 3 [file 44318_2025_634_MOESM9_ESM.zip › Figure 3/Source data_Figure 3G/ΔpdeI.tif]

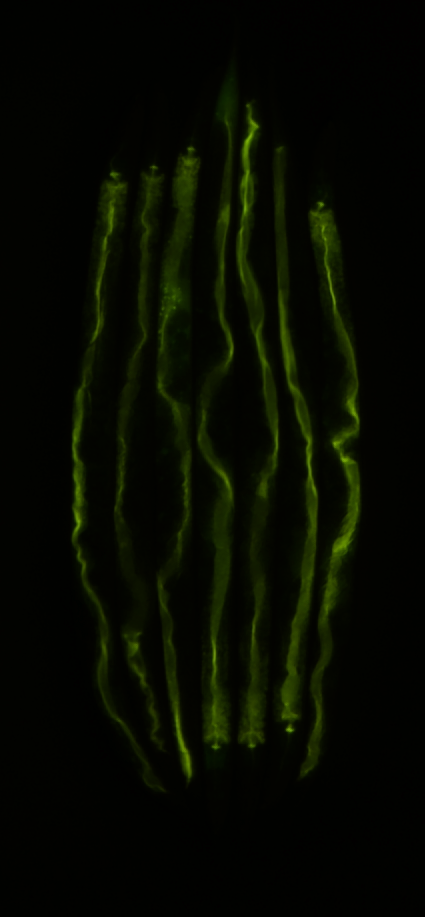

Supplement: Supplementary file 9 — Source data Fig. 3 [file 44318_2025_634_MOESM9_ESM.zip › Figure 3/Source data_Figure 3G/ΔtktA.tif]

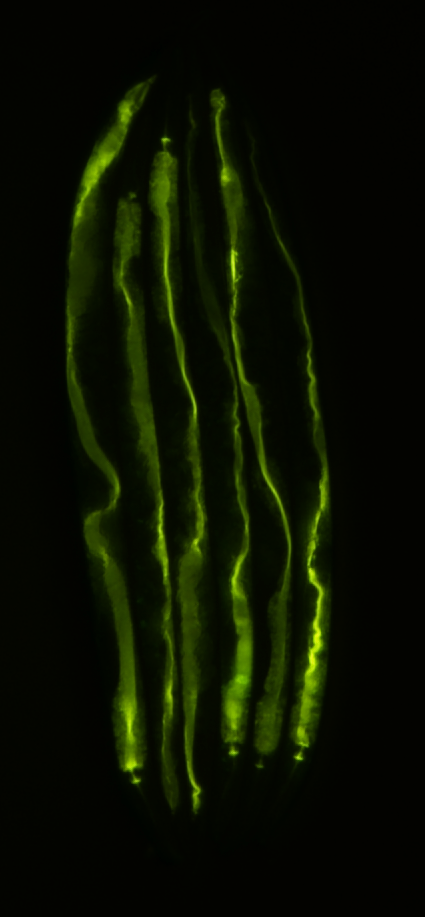

Supplement: Supplementary file 9 — Source data Fig. 3 [file 44318_2025_634_MOESM9_ESM.zip › Figure 3/Source data_Figure 3G/ΔyciA.tif]

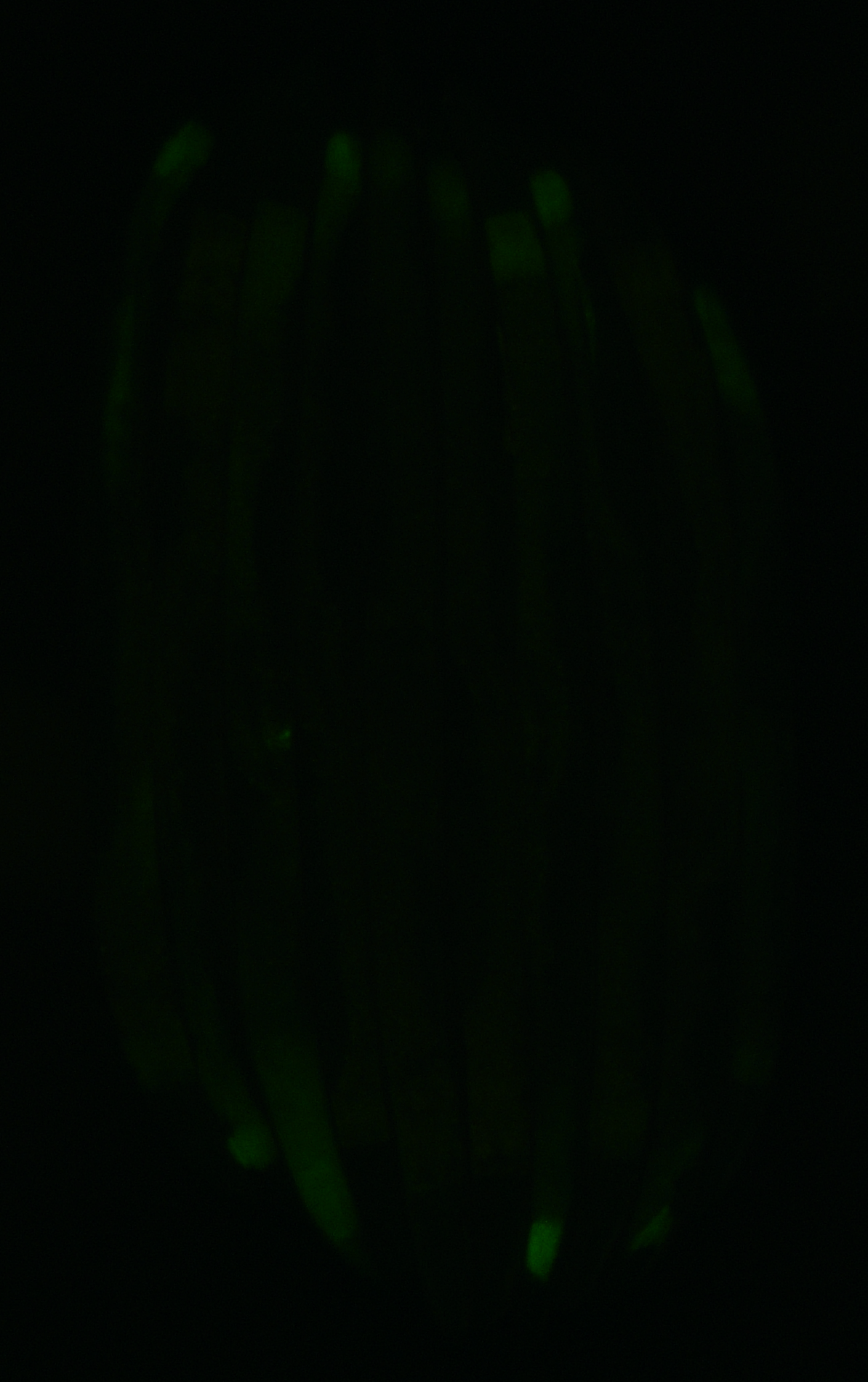

Supplement: Supplementary file 10 — Source data Fig. 4 [file 44318_2025_634_MOESM10_ESM.zip › Figure 4/Source data_Figure 4A/BW25113.tif]

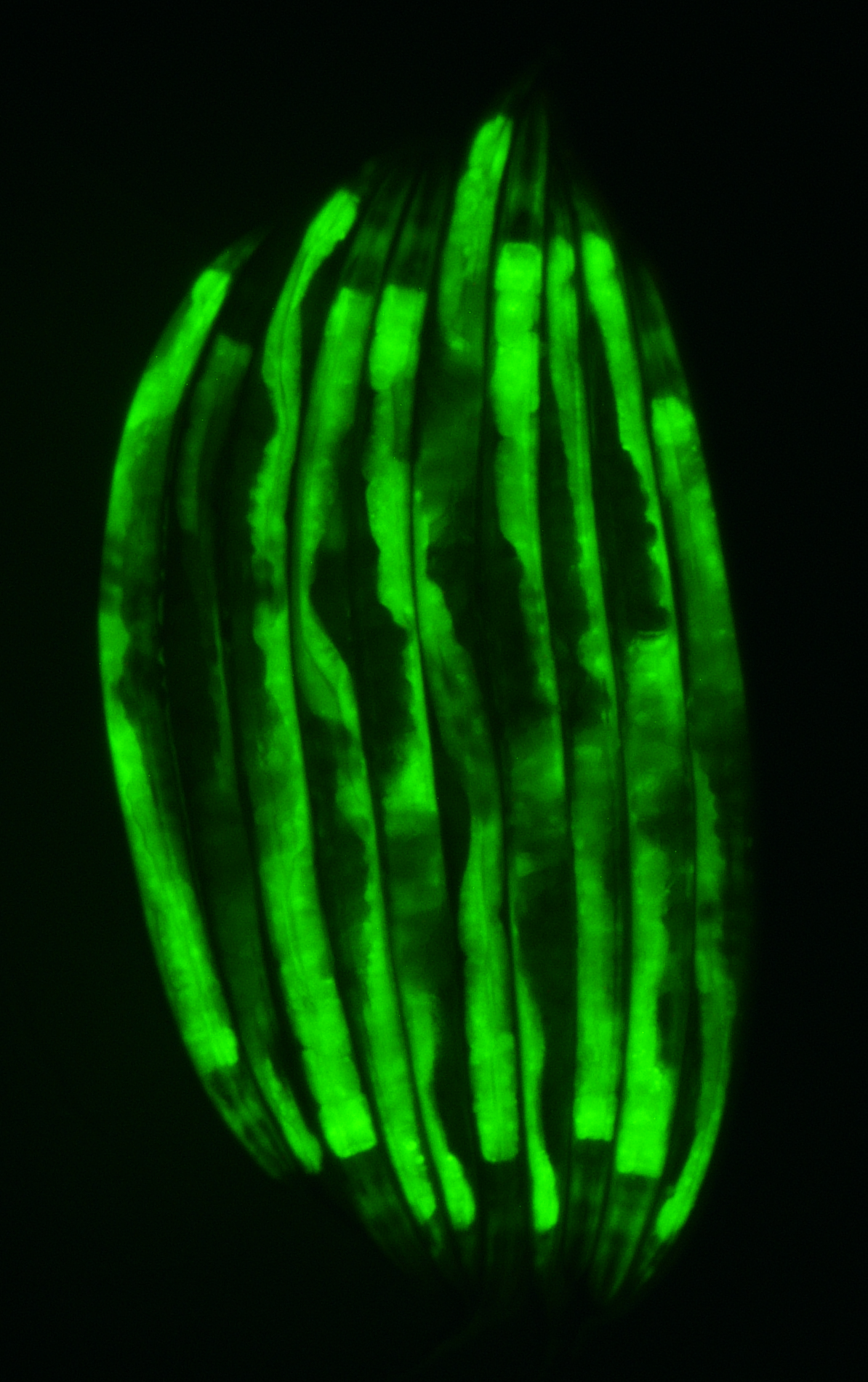

Supplement: Supplementary file 10 — Source data Fig. 4 [file 44318_2025_634_MOESM10_ESM.zip › Figure 4/Source data_Figure 4A/ΔallD.tif]

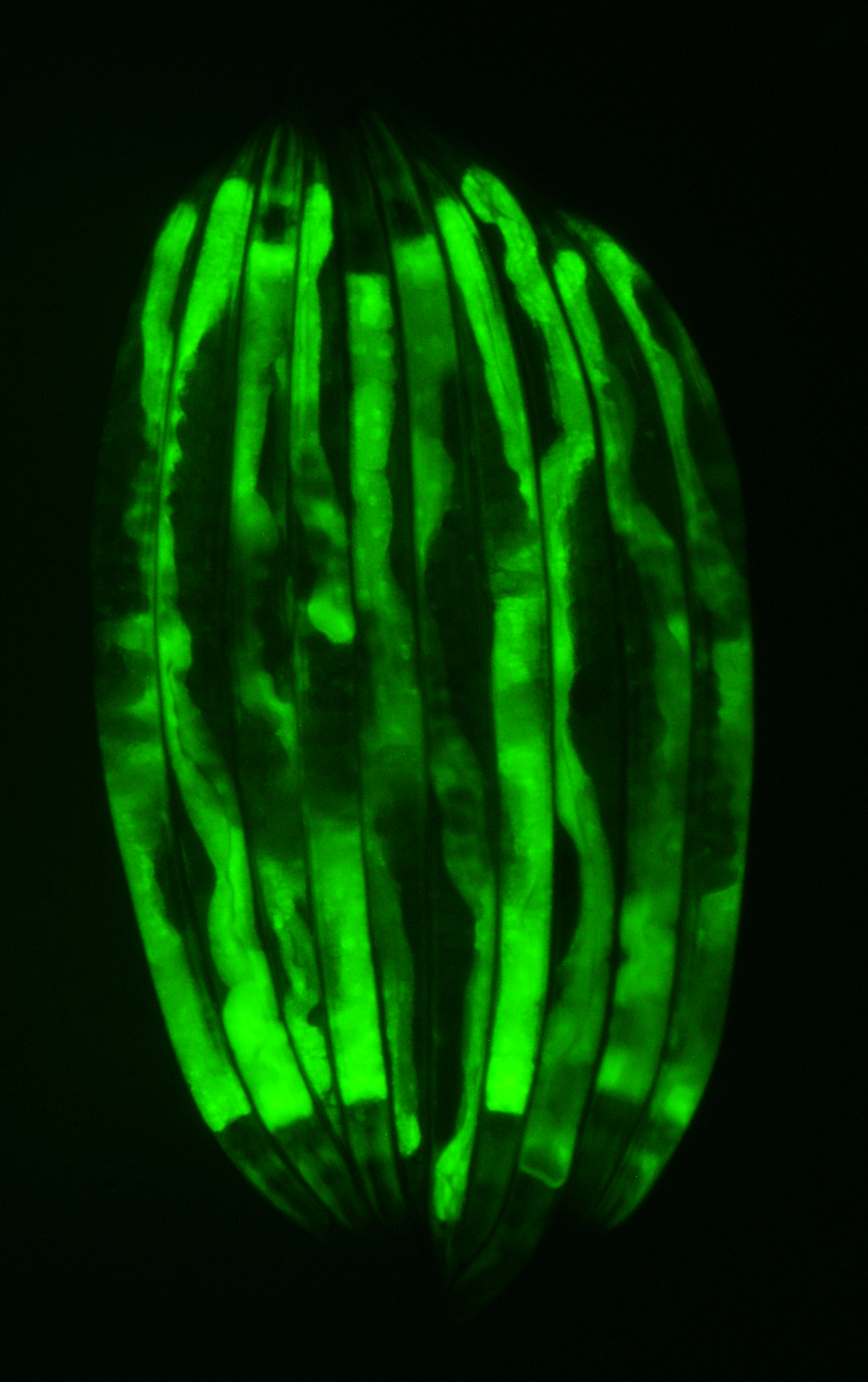

Supplement: Supplementary file 10 — Source data Fig. 4 [file 44318_2025_634_MOESM10_ESM.zip › Figure 4/Source data_Figure 4A/ΔcpxR.tif]

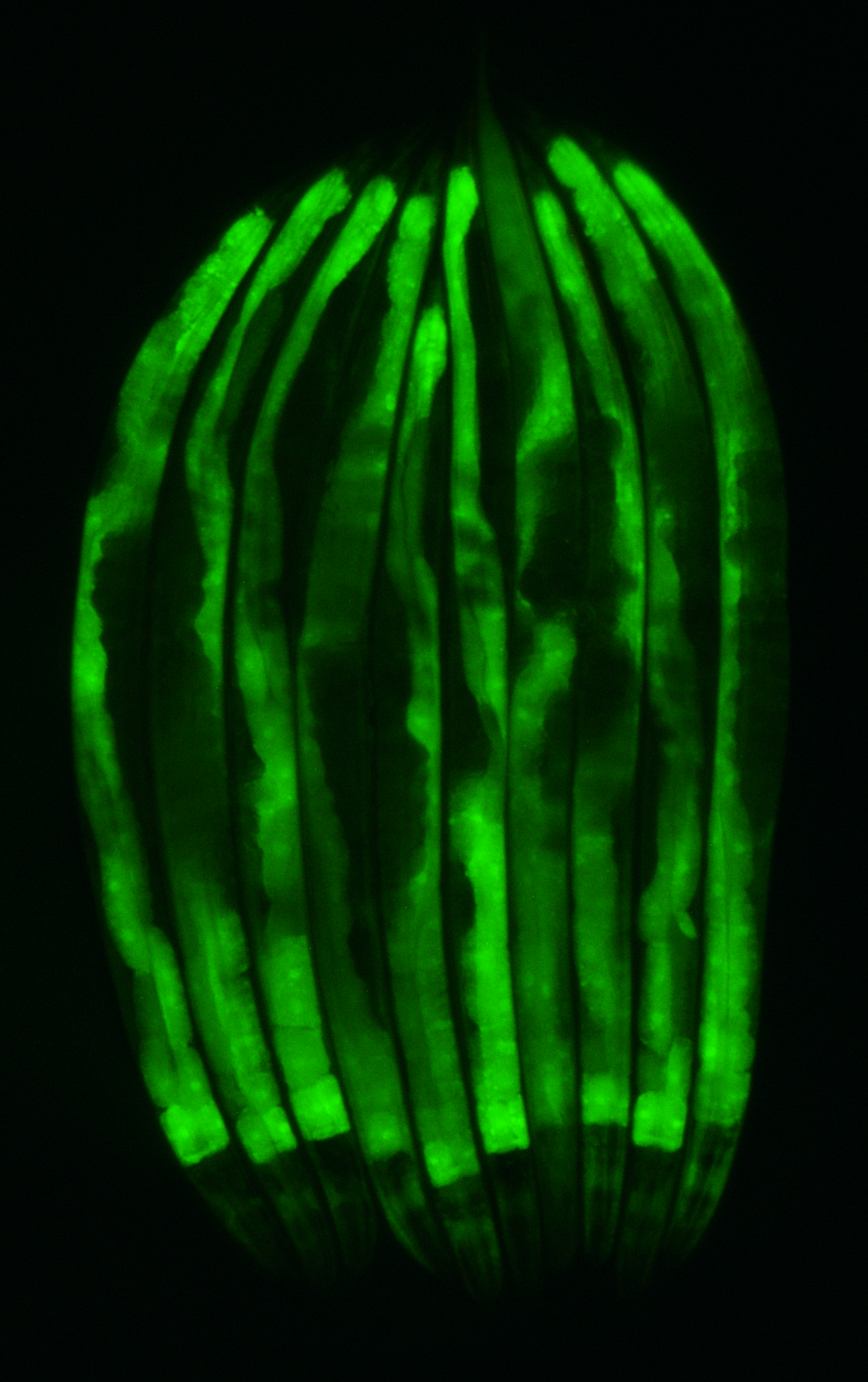

Supplement: Supplementary file 10 — Source data Fig. 4 [file 44318_2025_634_MOESM10_ESM.zip › Figure 4/Source data_Figure 4A/ΔcutC.tif]

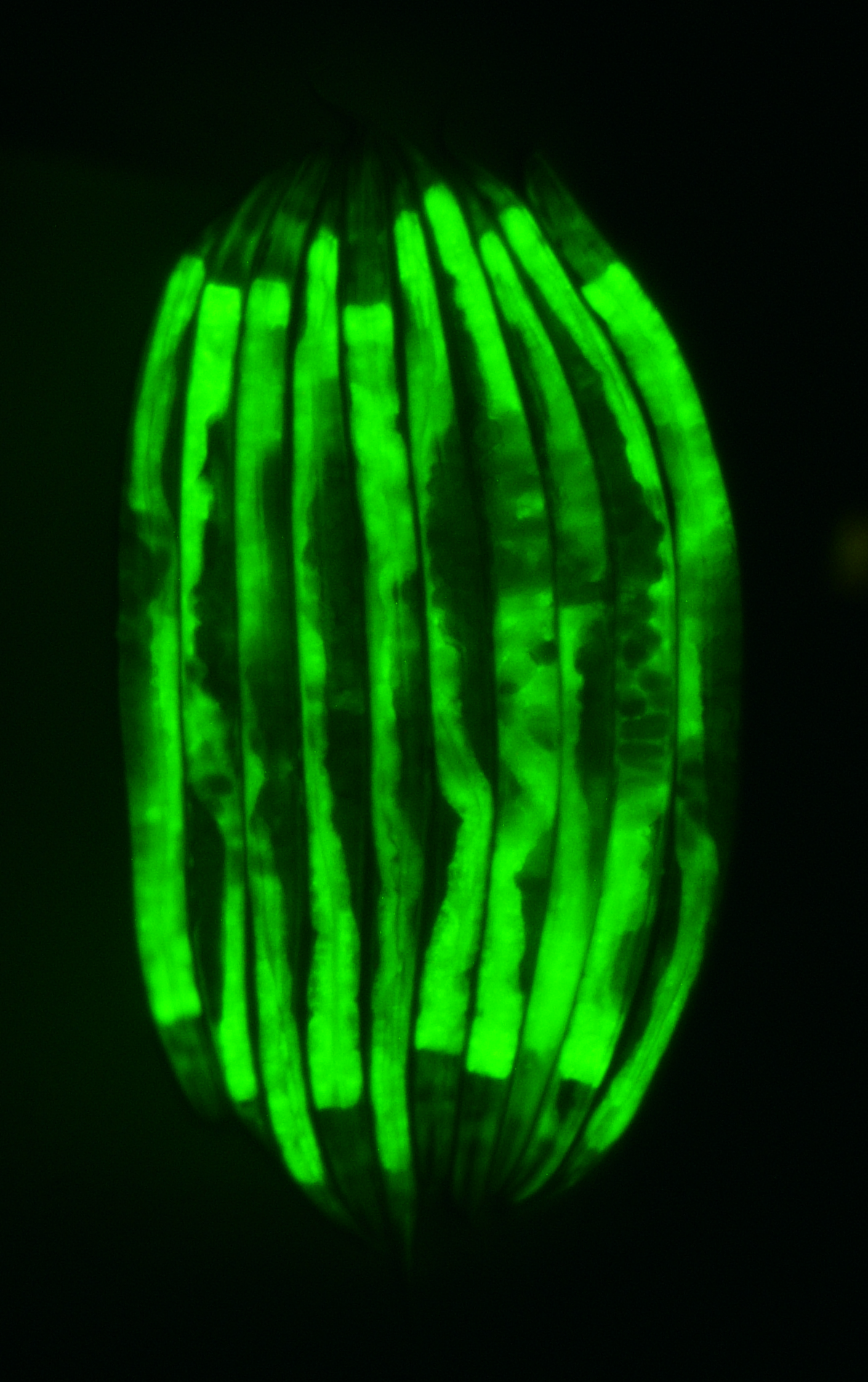

Supplement: Supplementary file 10 — Source data Fig. 4 [file 44318_2025_634_MOESM10_ESM.zip › Figure 4/Source data_Figure 4A/ΔcyoA.tif]

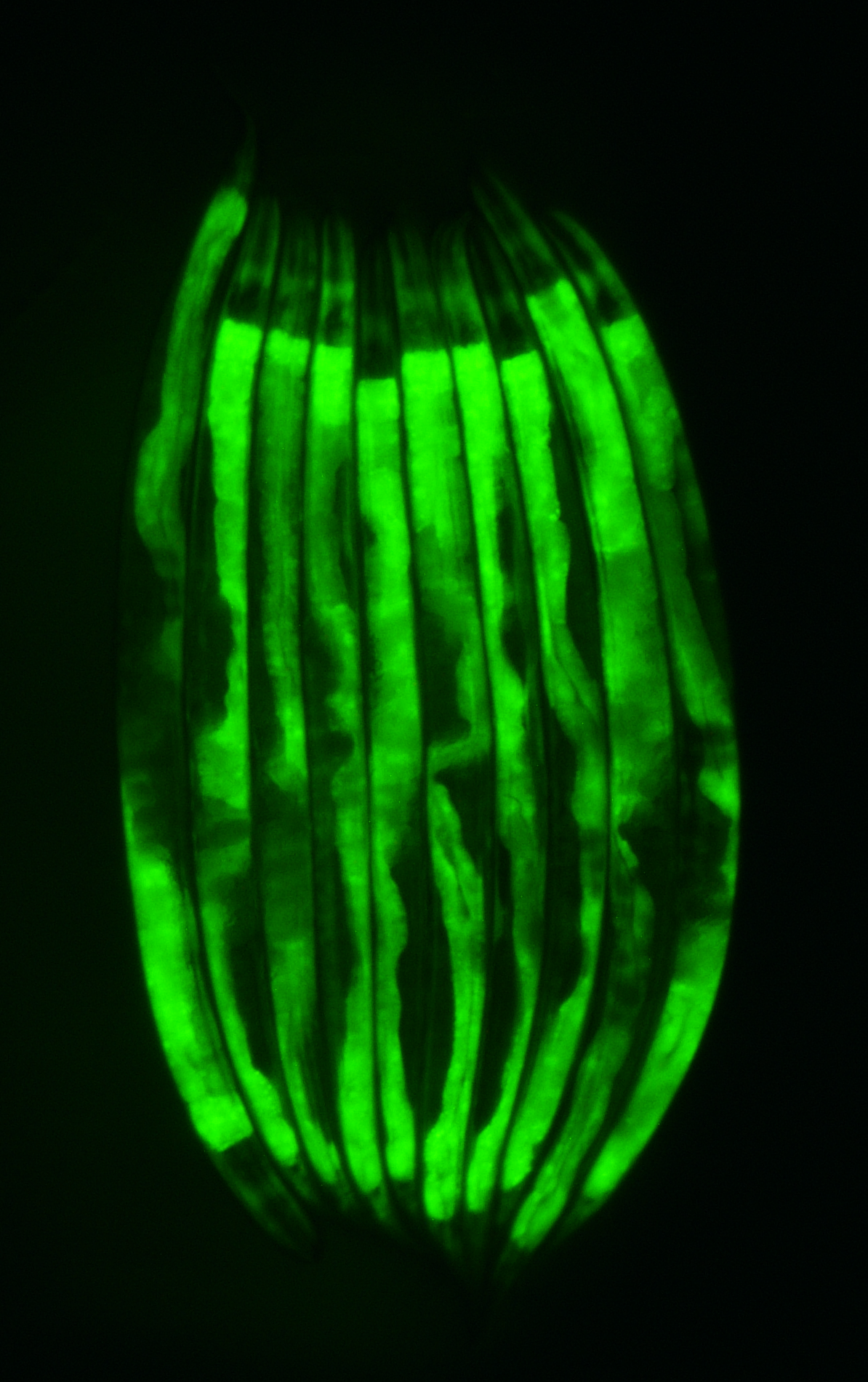

Supplement: Supplementary file 10 — Source data Fig. 4 [file 44318_2025_634_MOESM10_ESM.zip › Figure 4/Source data_Figure 4A/ΔcyoB.tif]

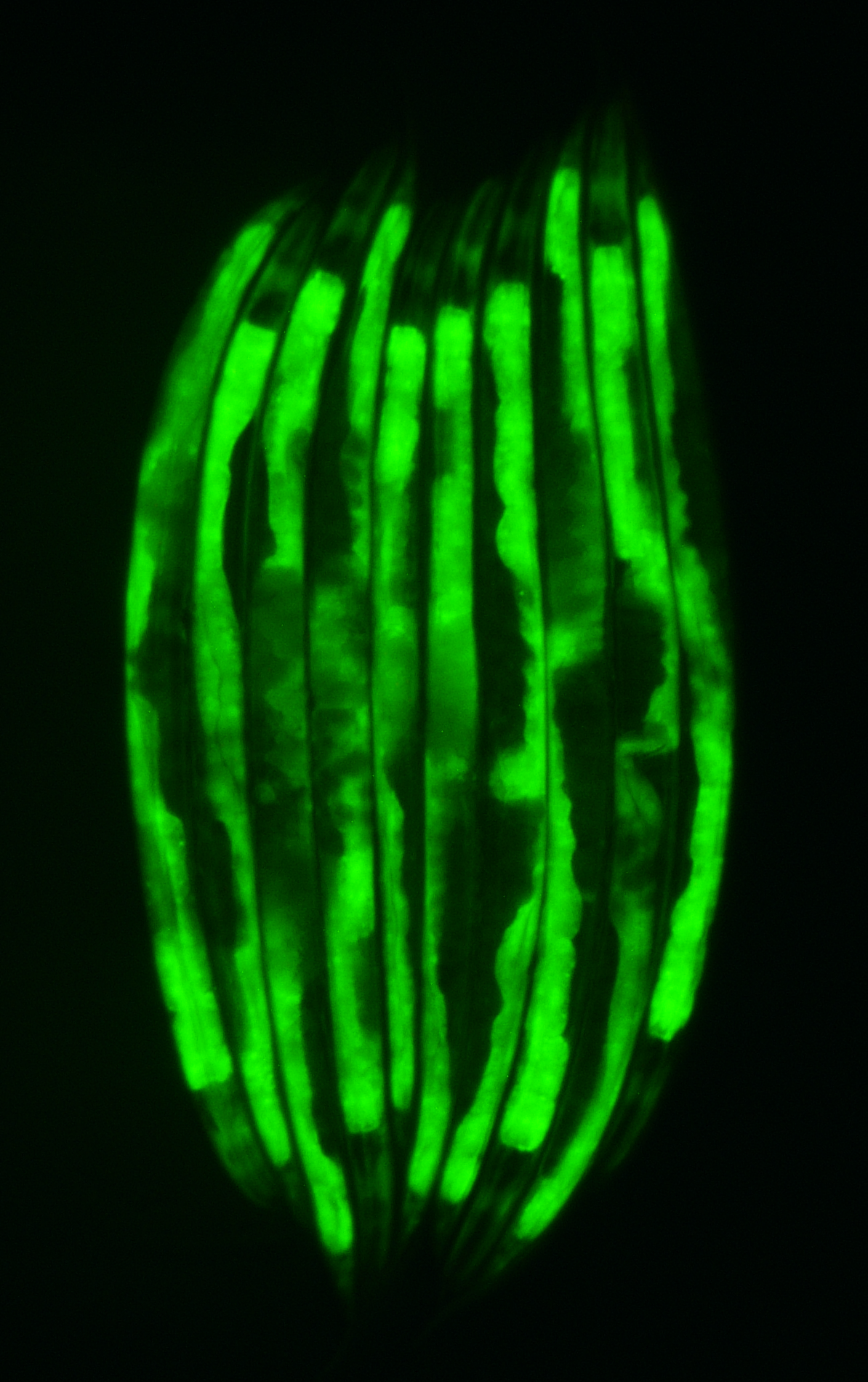

Supplement: Supplementary file 10 — Source data Fig. 4 [file 44318_2025_634_MOESM10_ESM.zip › Figure 4/Source data_Figure 4A/ΔcyoC.tif]

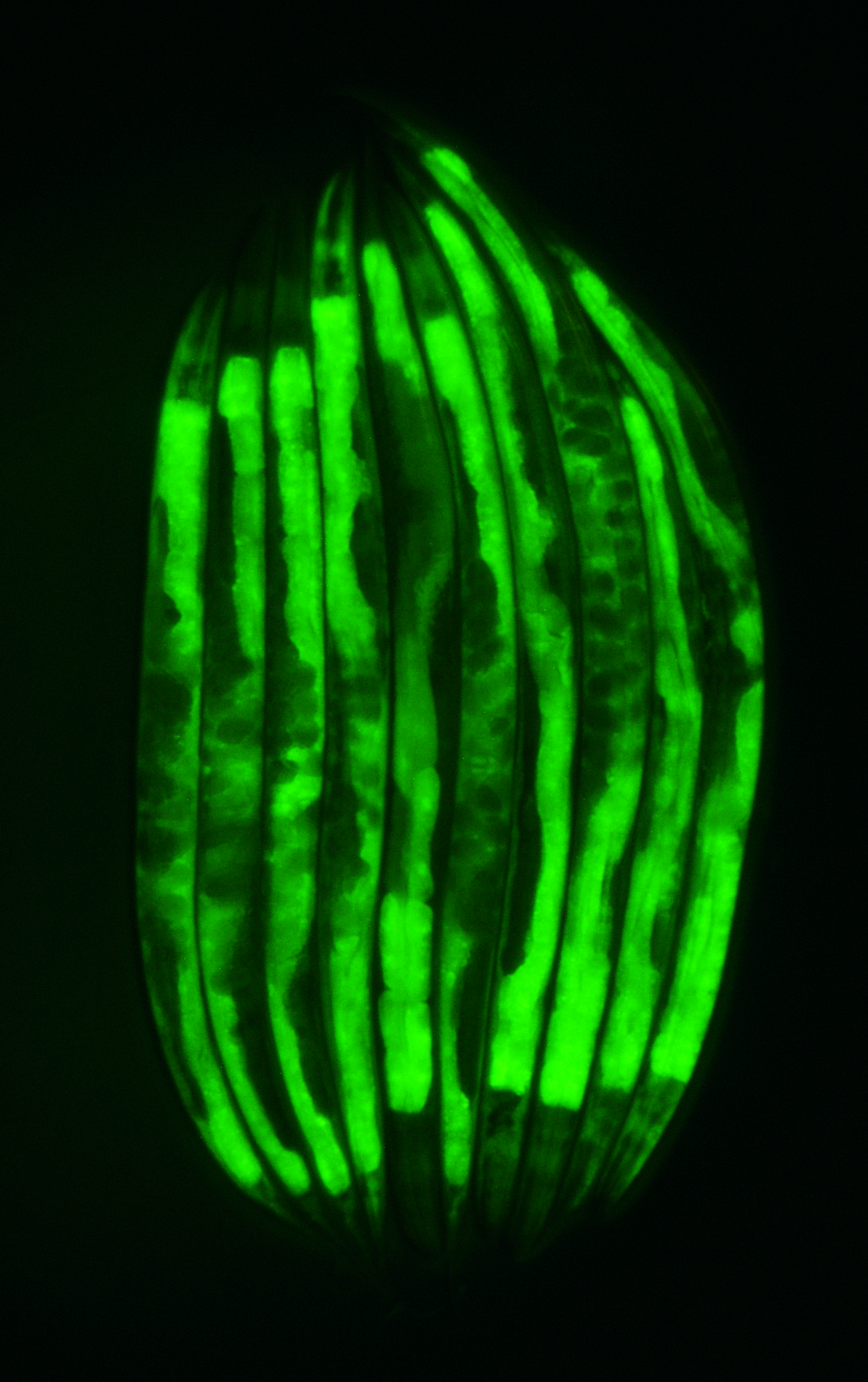

Supplement: Supplementary file 10 — Source data Fig. 4 [file 44318_2025_634_MOESM10_ESM.zip › Figure 4/Source data_Figure 4A/ΔcyoD.tif]

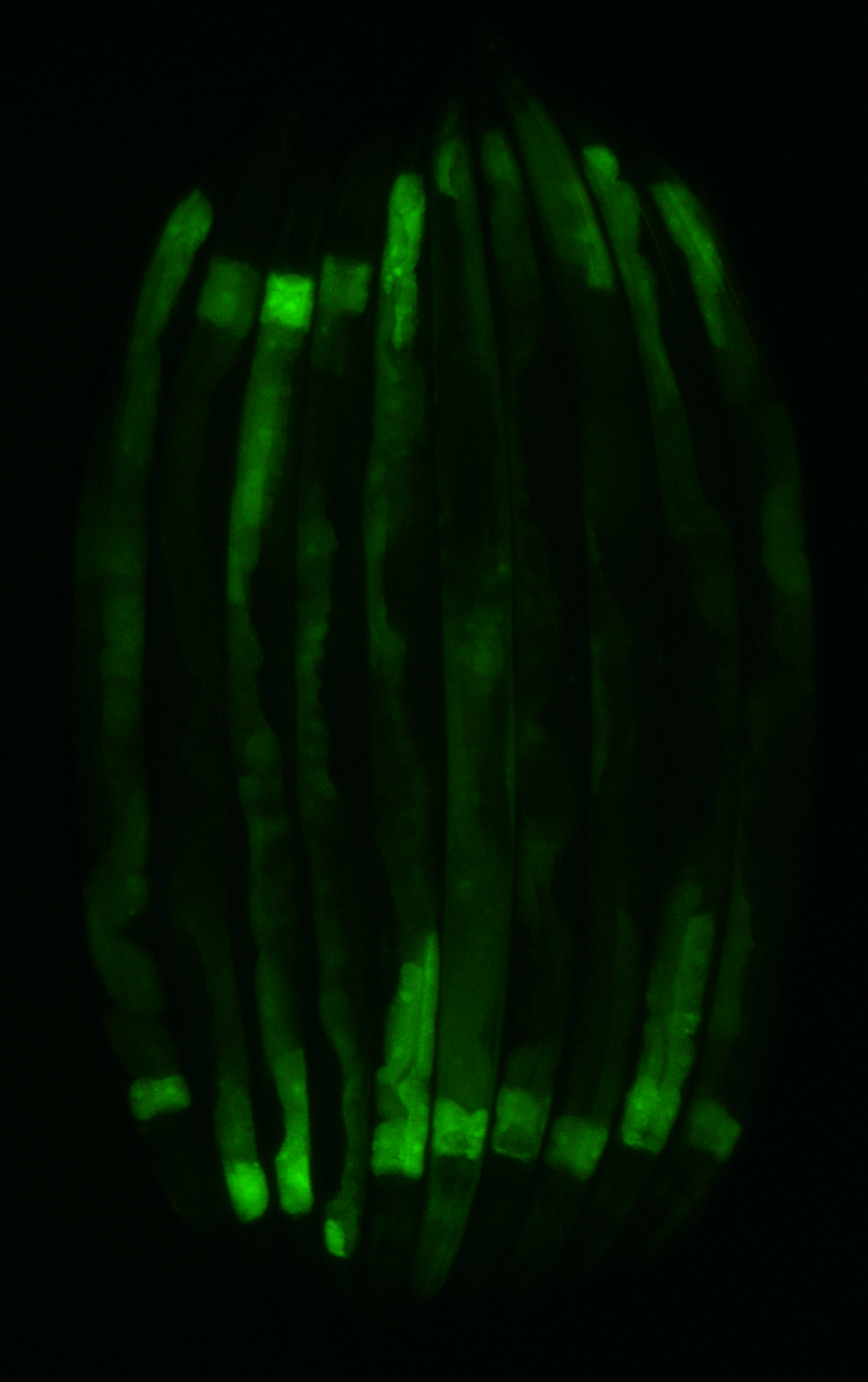

Supplement: Supplementary file 10 — Source data Fig. 4 [file 44318_2025_634_MOESM10_ESM.zip › Figure 4/Source data_Figure 4A/ΔdmsA.tif]

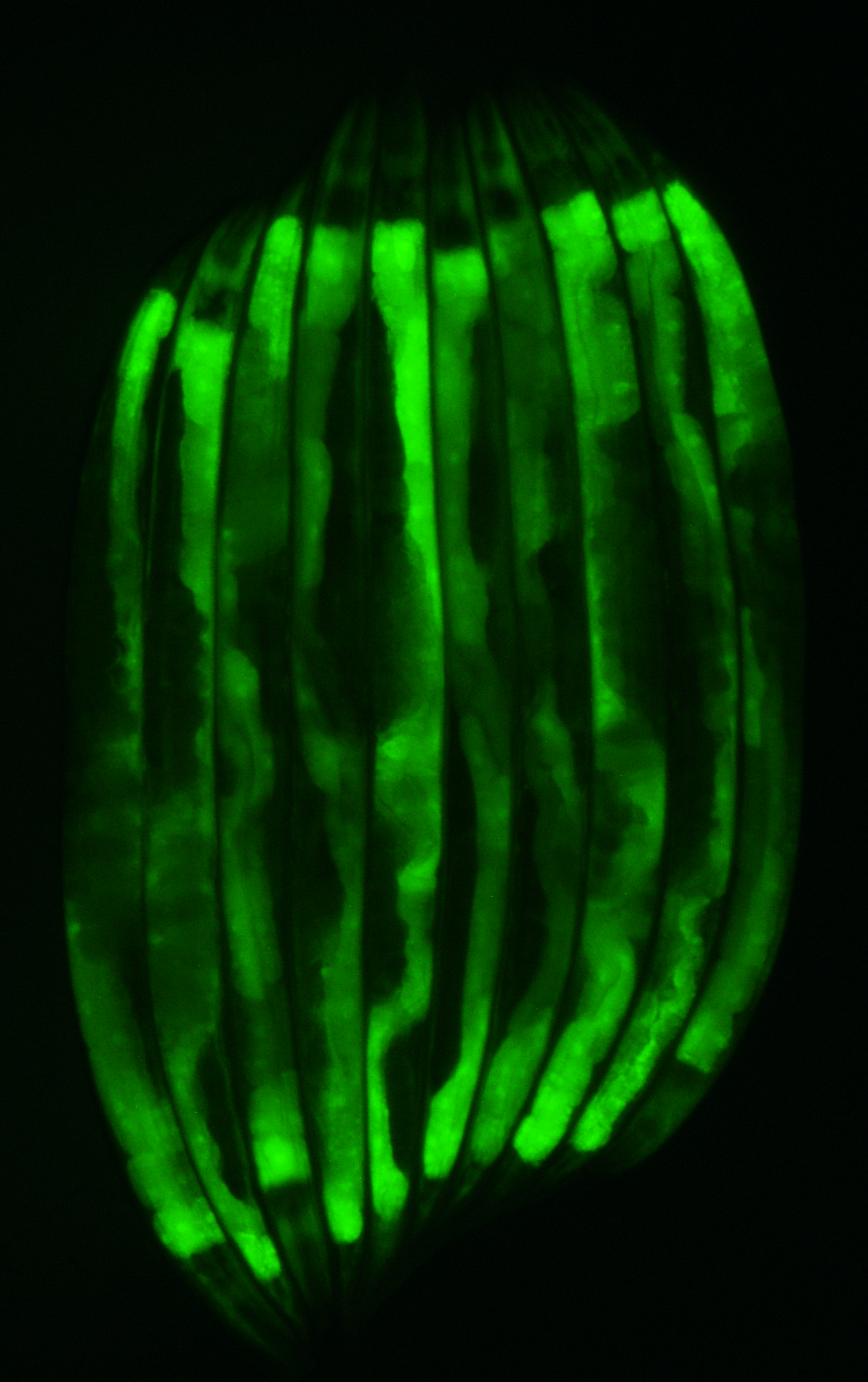

Supplement: Supplementary file 10 — Source data Fig. 4 [file 44318_2025_634_MOESM10_ESM.zip › Figure 4/Source data_Figure 4A/ΔfdrA .tif]

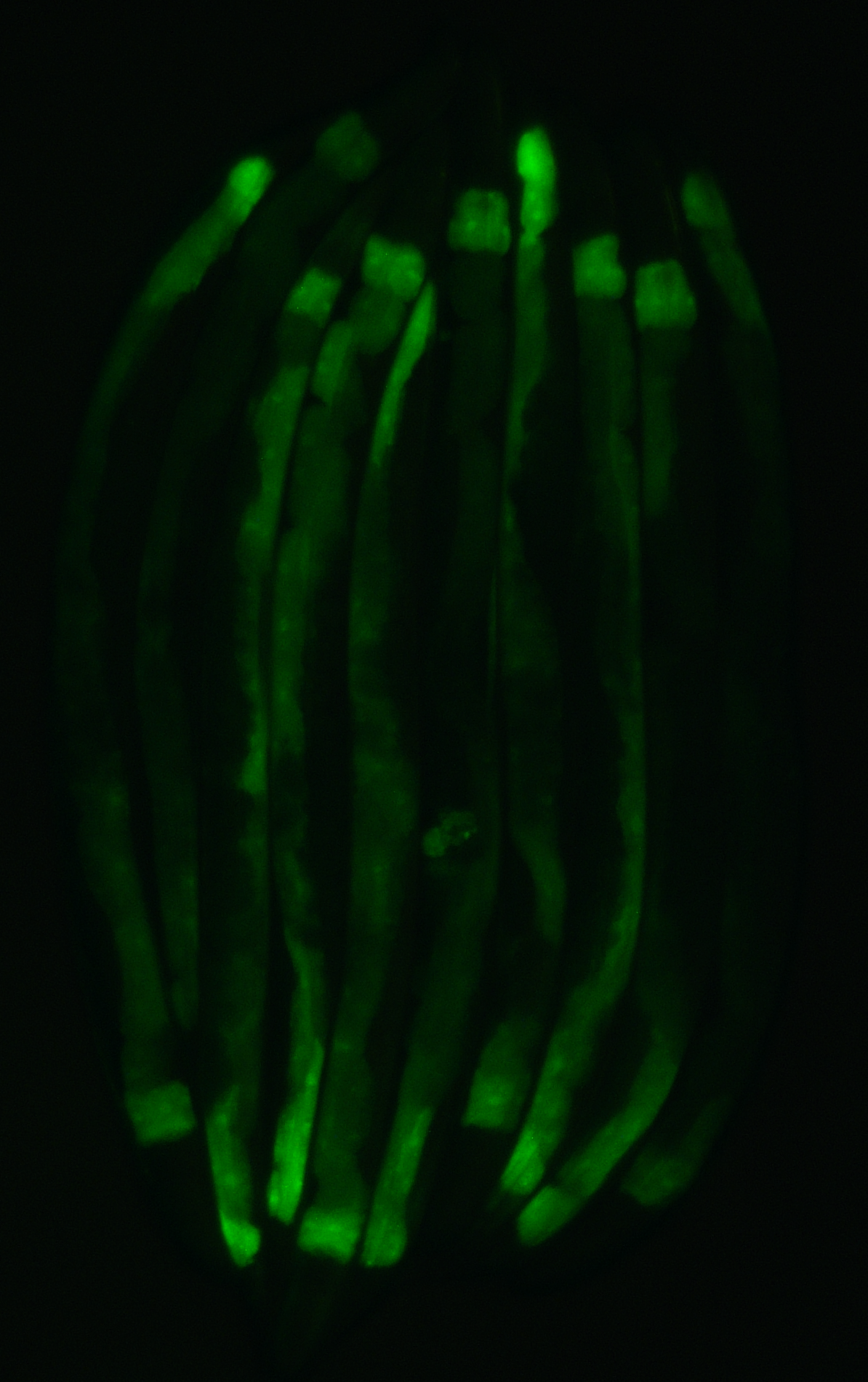

Supplement: Supplementary file 10 — Source data Fig. 4 [file 44318_2025_634_MOESM10_ESM.zip › Figure 4/Source data_Figure 4A/ΔnarY.tif]

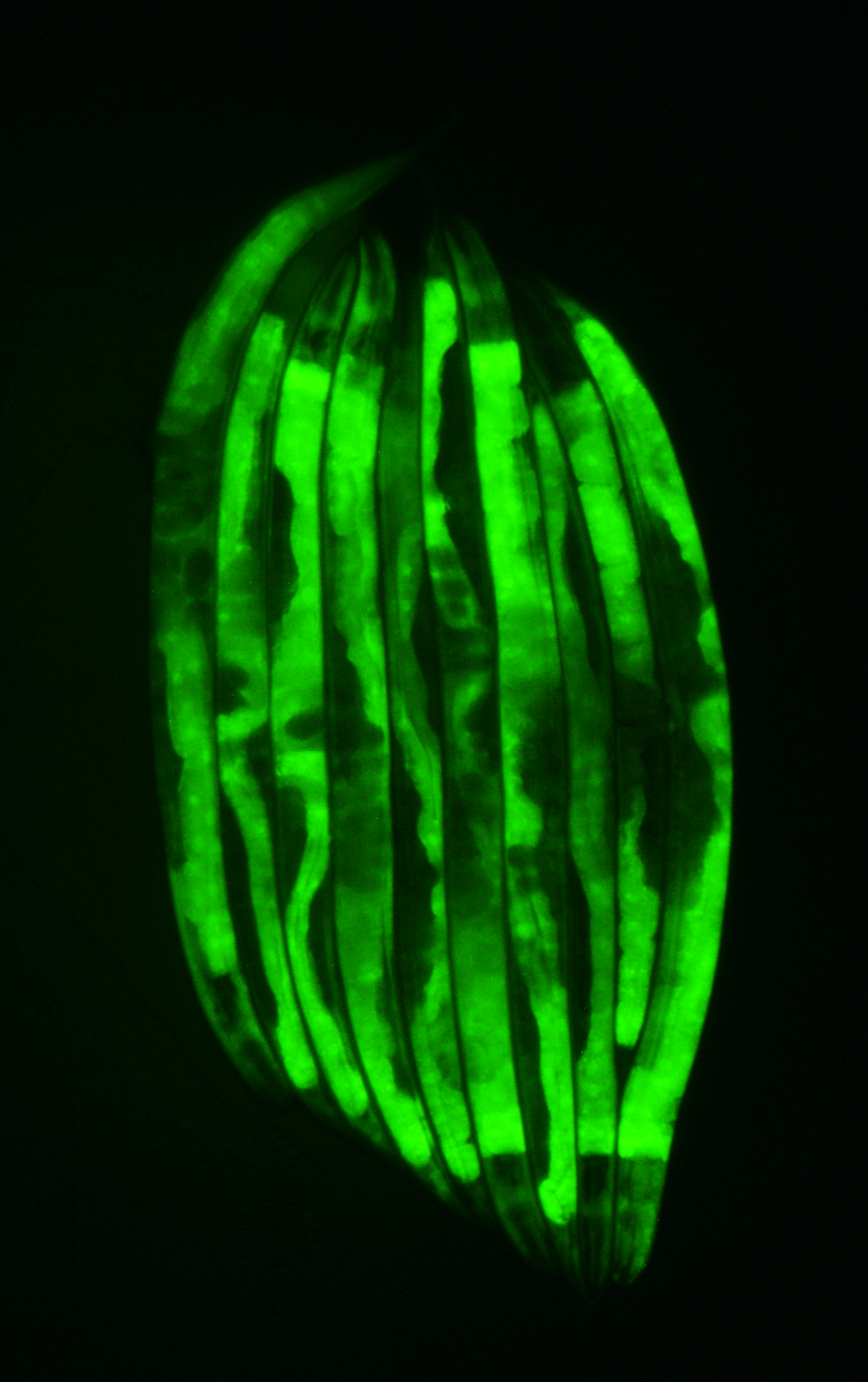

Supplement: Supplementary file 10 — Source data Fig. 4 [file 44318_2025_634_MOESM10_ESM.zip › Figure 4/Source data_Figure 4A/ΔpdeI.tif]

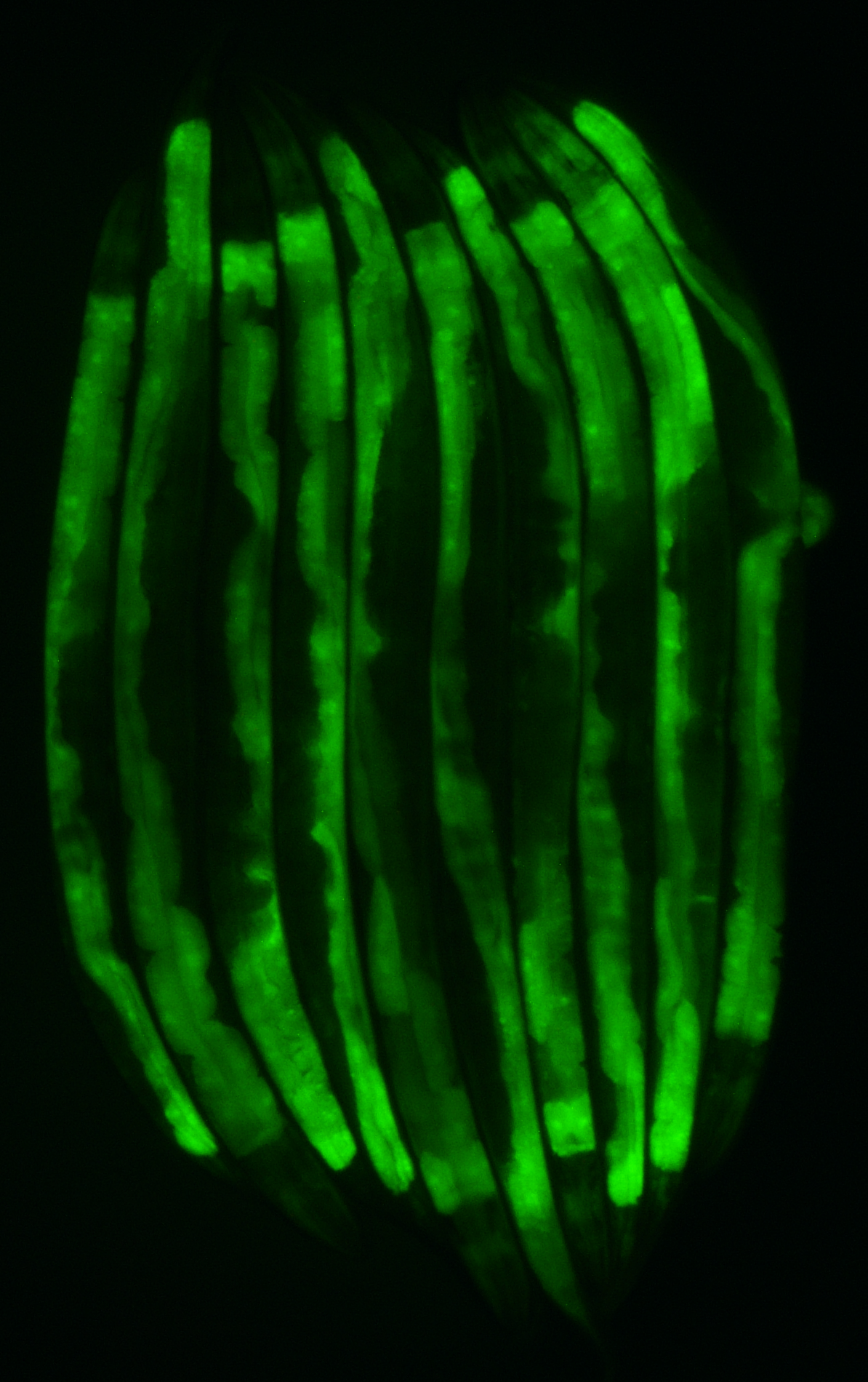

Supplement: Supplementary file 10 — Source data Fig. 4 [file 44318_2025_634_MOESM10_ESM.zip › Figure 4/Source data_Figure 4A/ΔpliG .tif]

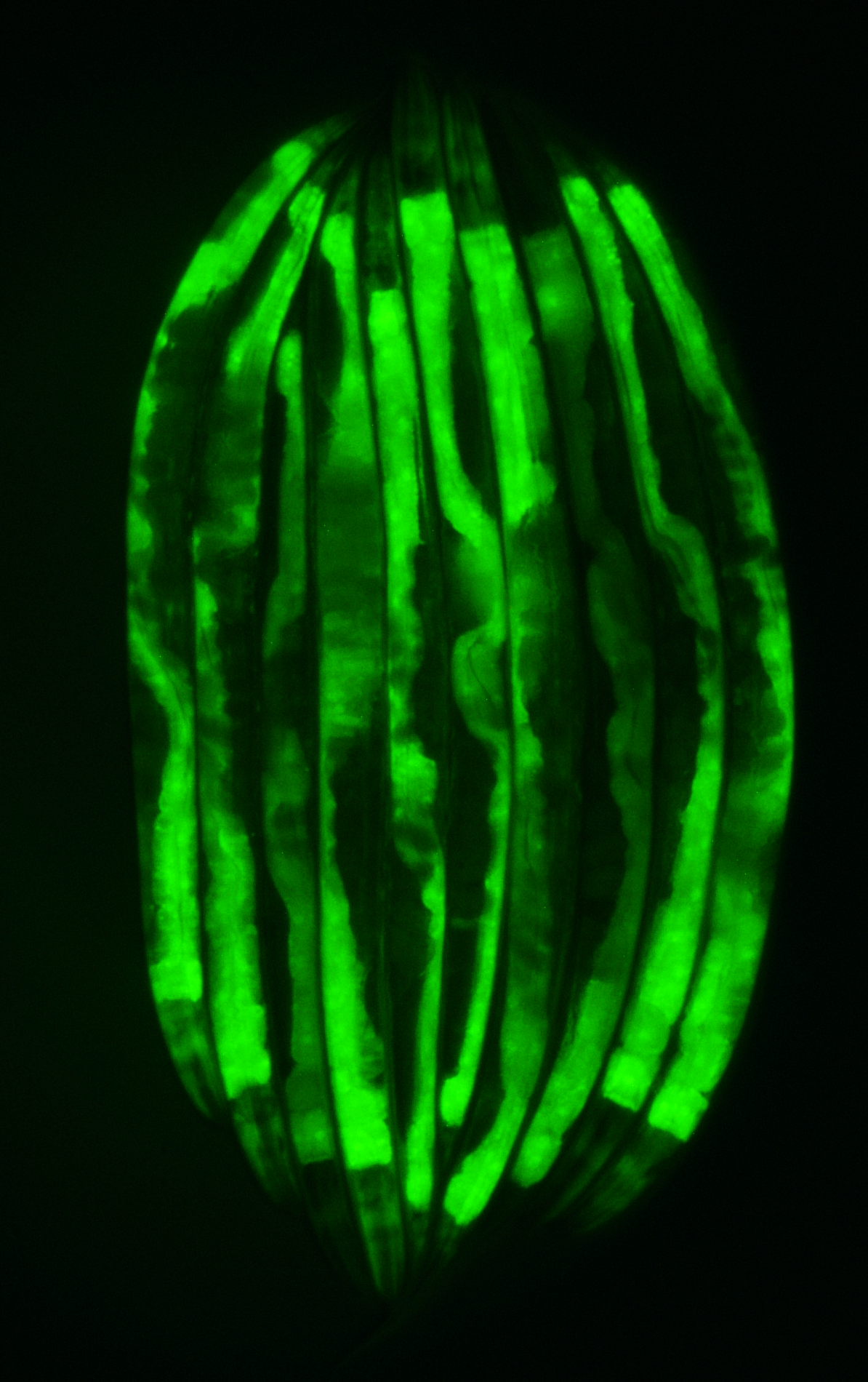

Supplement: Supplementary file 10 — Source data Fig. 4 [file 44318_2025_634_MOESM10_ESM.zip › Figure 4/Source data_Figure 4A/ΔrlmL.tif]

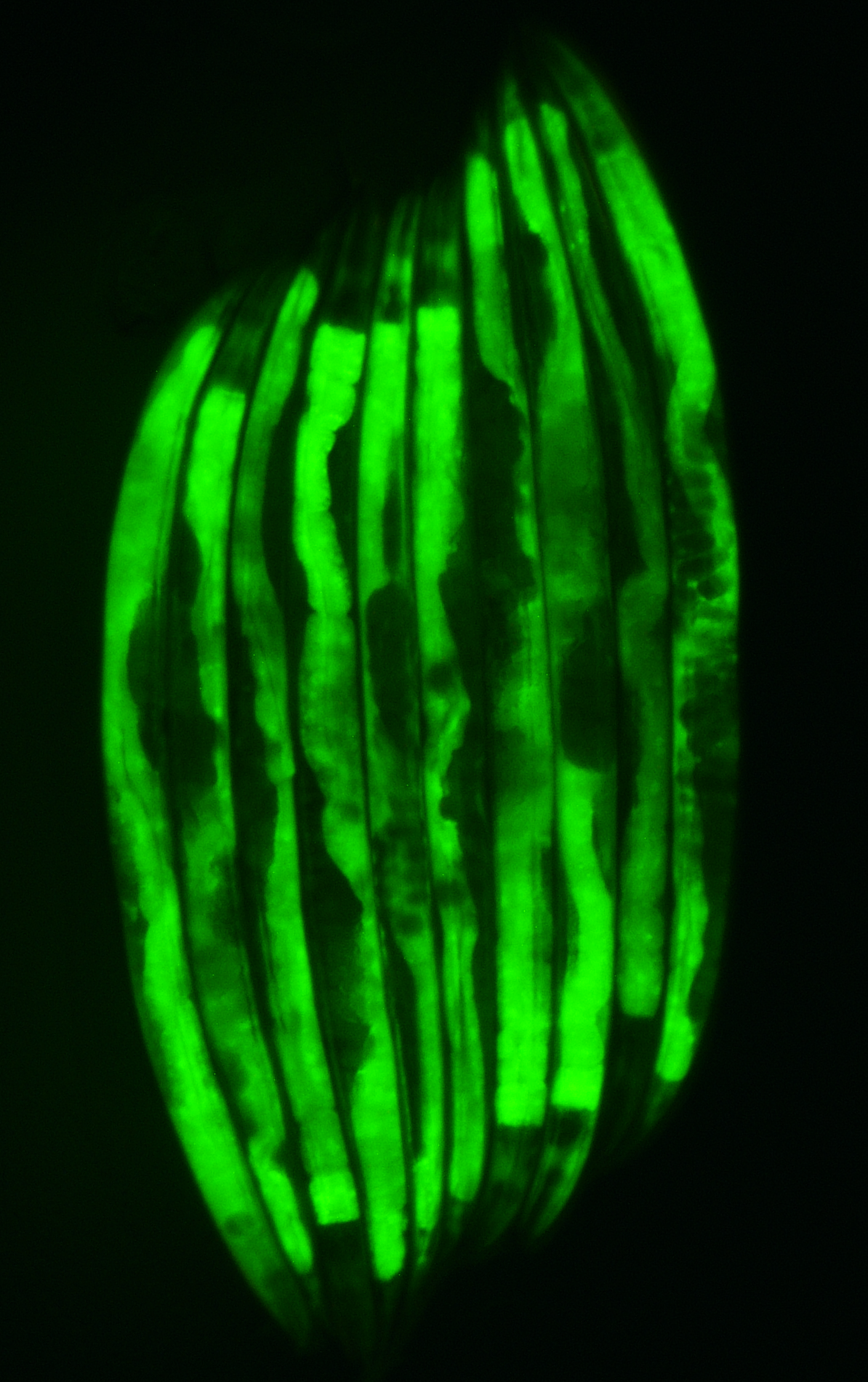

Supplement: Supplementary file 10 — Source data Fig. 4 [file 44318_2025_634_MOESM10_ESM.zip › Figure 4/Source data_Figure 4A/ΔroxA.tif]

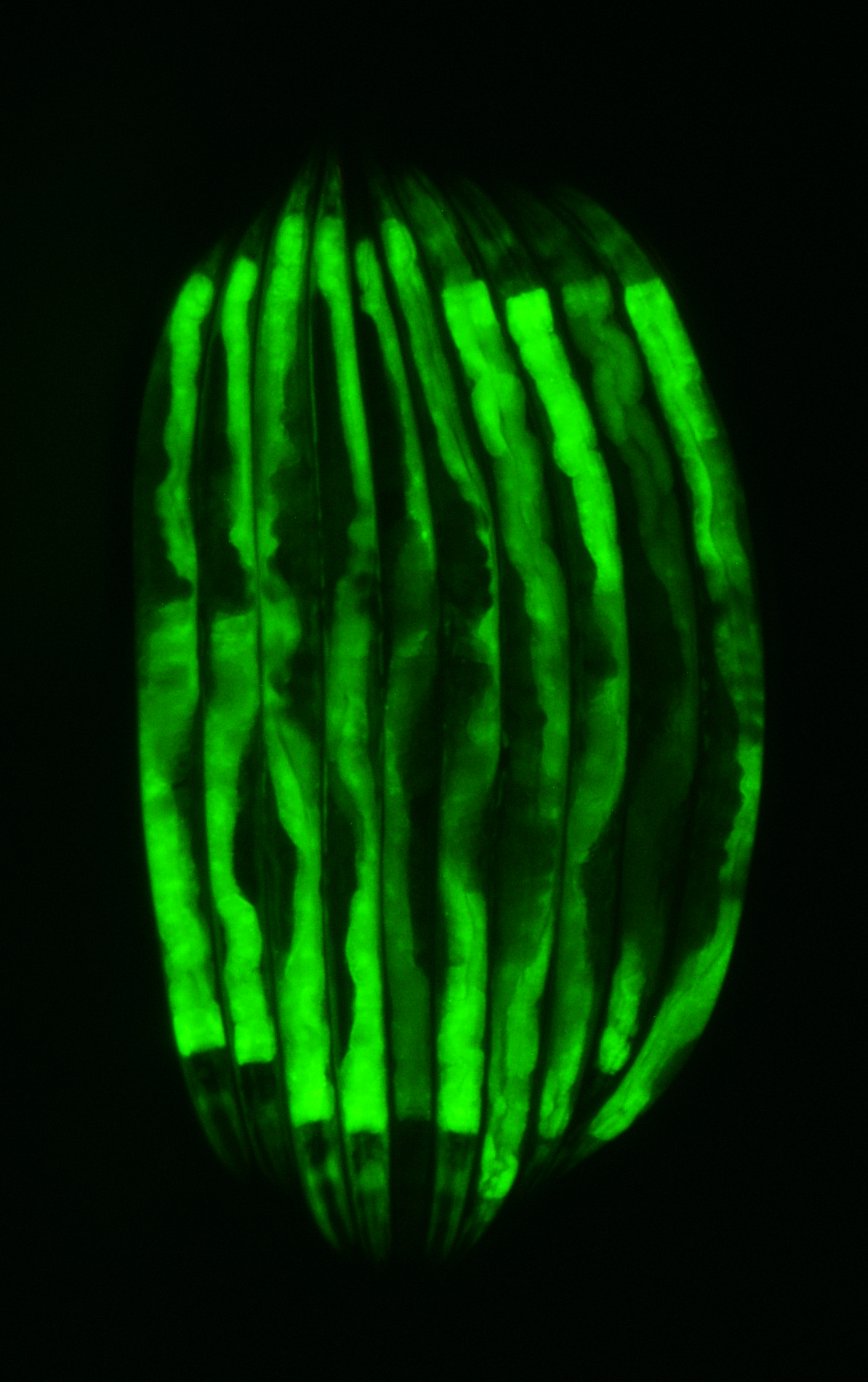

Supplement: Supplementary file 10 — Source data Fig. 4 [file 44318_2025_634_MOESM10_ESM.zip › Figure 4/Source data_Figure 4A/ΔsdhB.tif]

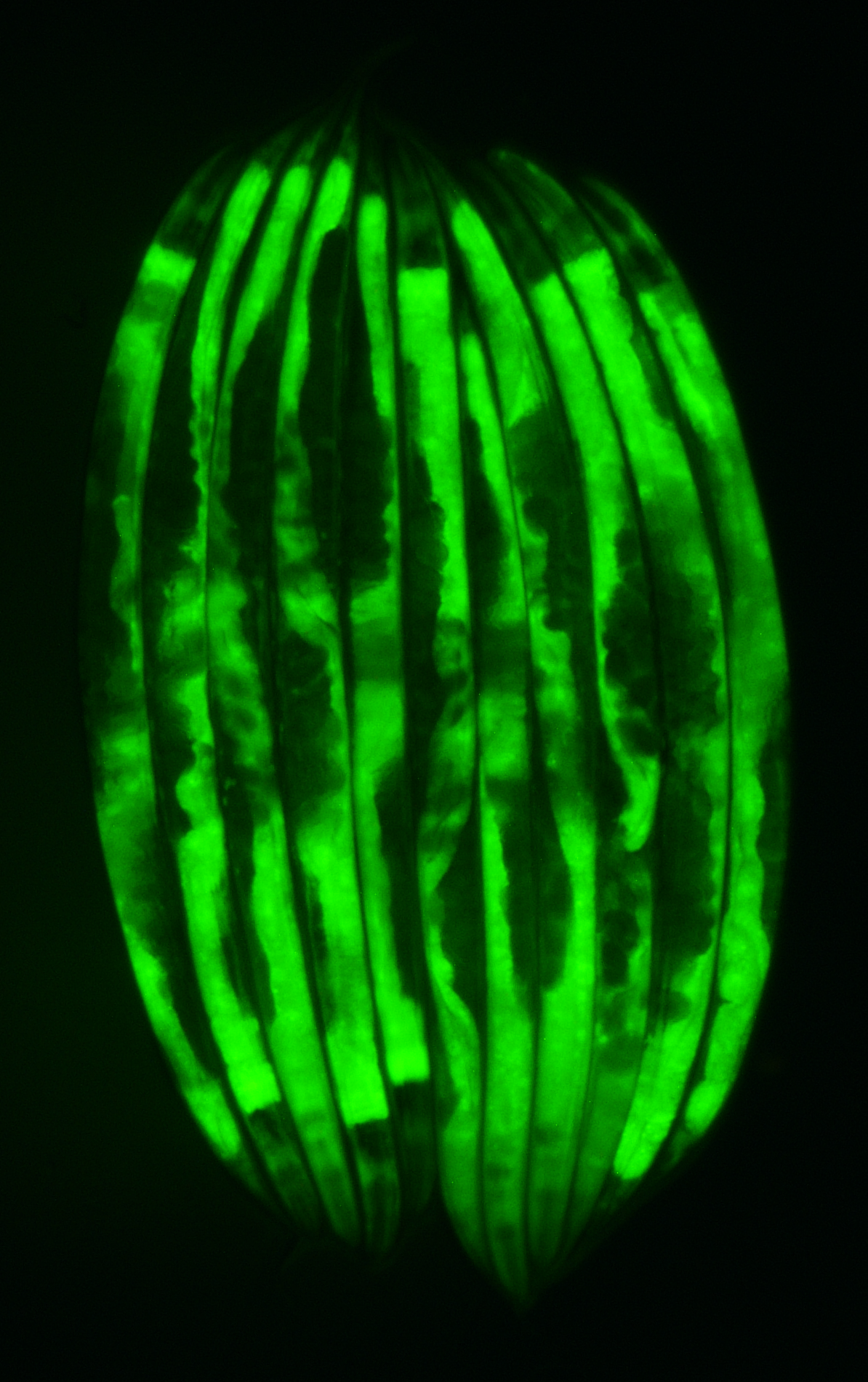

Supplement: Supplementary file 10 — Source data Fig. 4 [file 44318_2025_634_MOESM10_ESM.zip › Figure 4/Source data_Figure 4A/ΔstfP.tif]

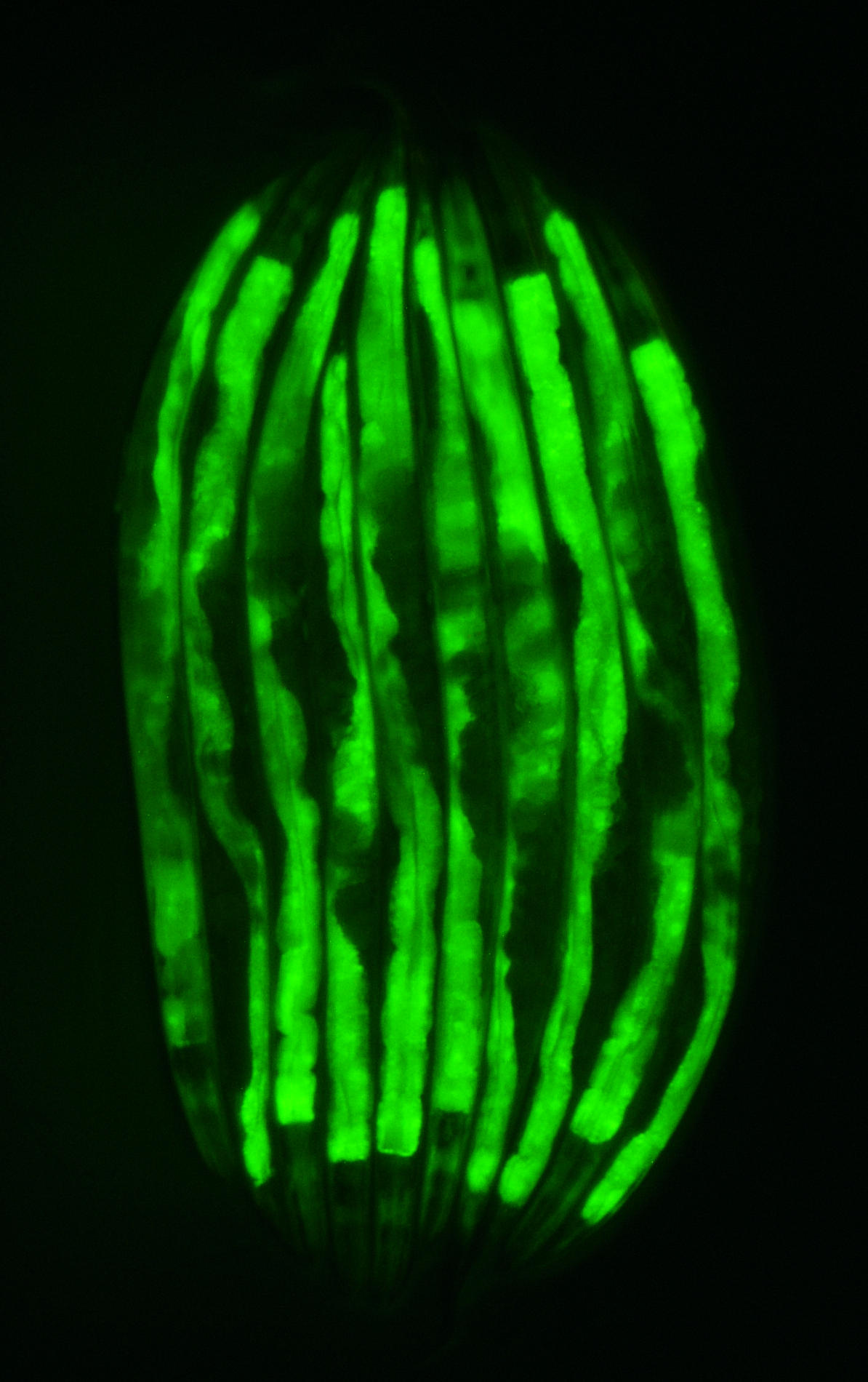

Supplement: Supplementary file 10 — Source data Fig. 4 [file 44318_2025_634_MOESM10_ESM.zip › Figure 4/Source data_Figure 4A/ΔtktA.tif]

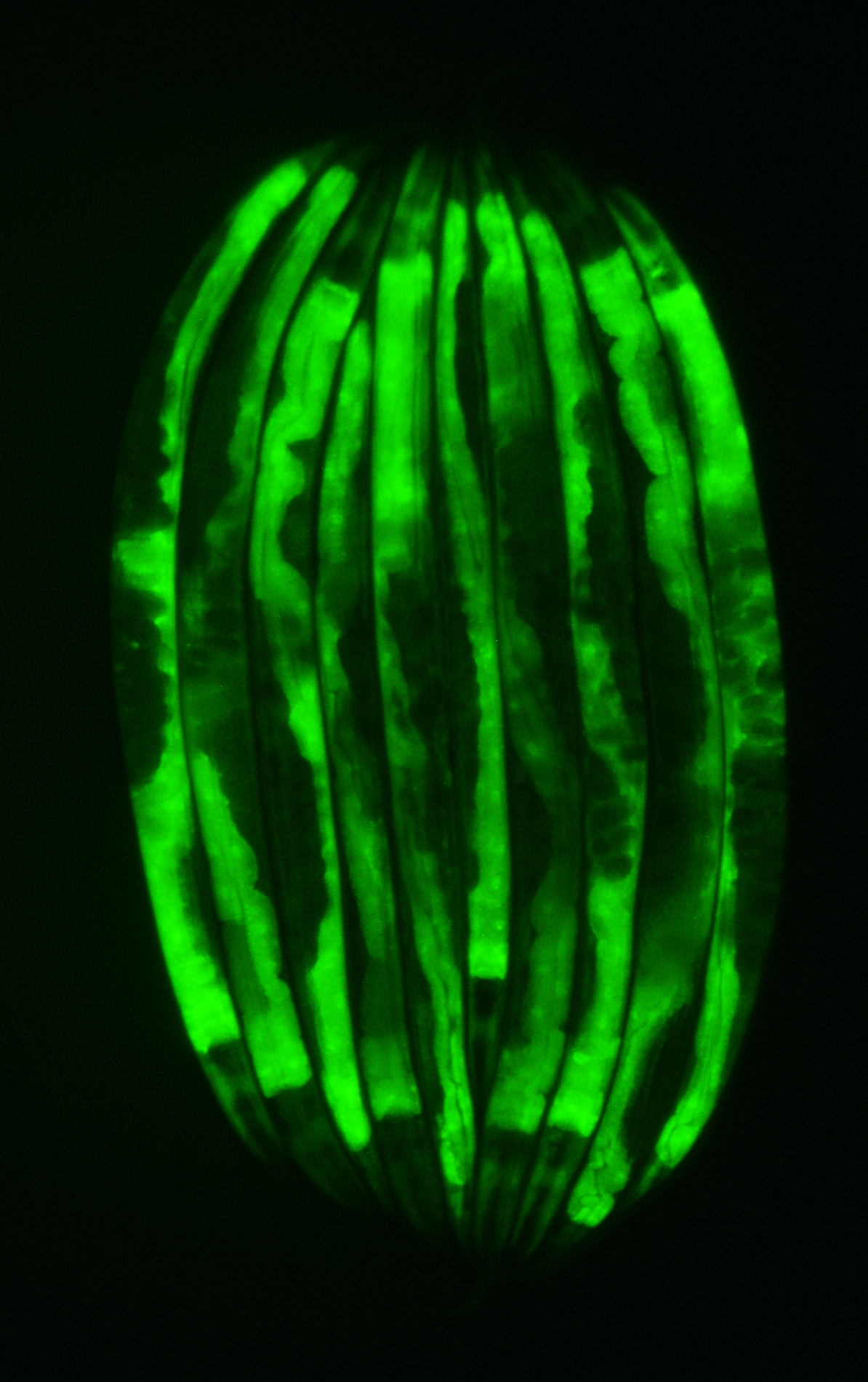

Supplement: Supplementary file 10 — Source data Fig. 4 [file 44318_2025_634_MOESM10_ESM.zip › Figure 4/Source data_Figure 4A/ΔtqsA.tif]

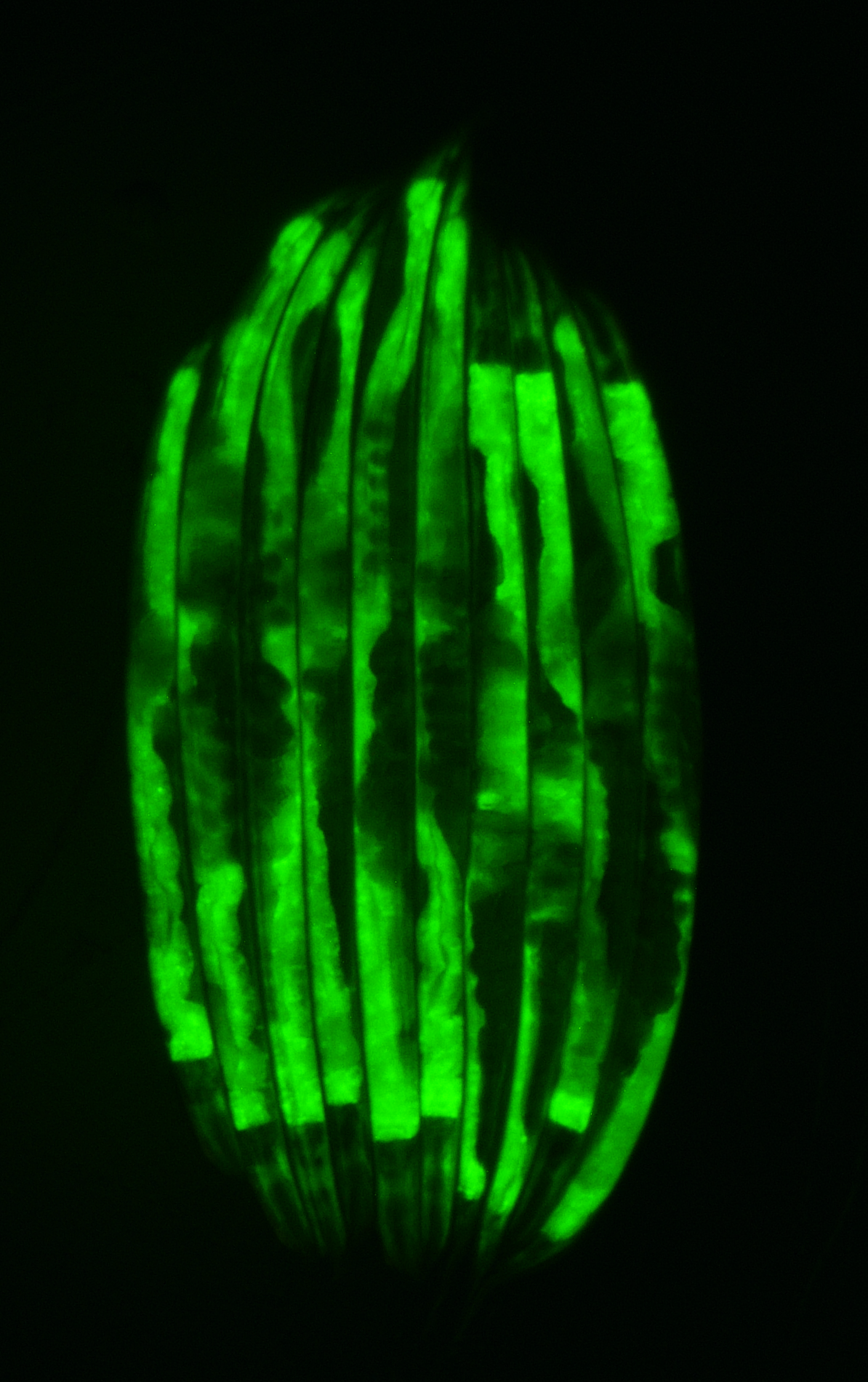

Supplement: Supplementary file 10 — Source data Fig. 4 [file 44318_2025_634_MOESM10_ESM.zip › Figure 4/Source data_Figure 4A/ΔybaN.tif]

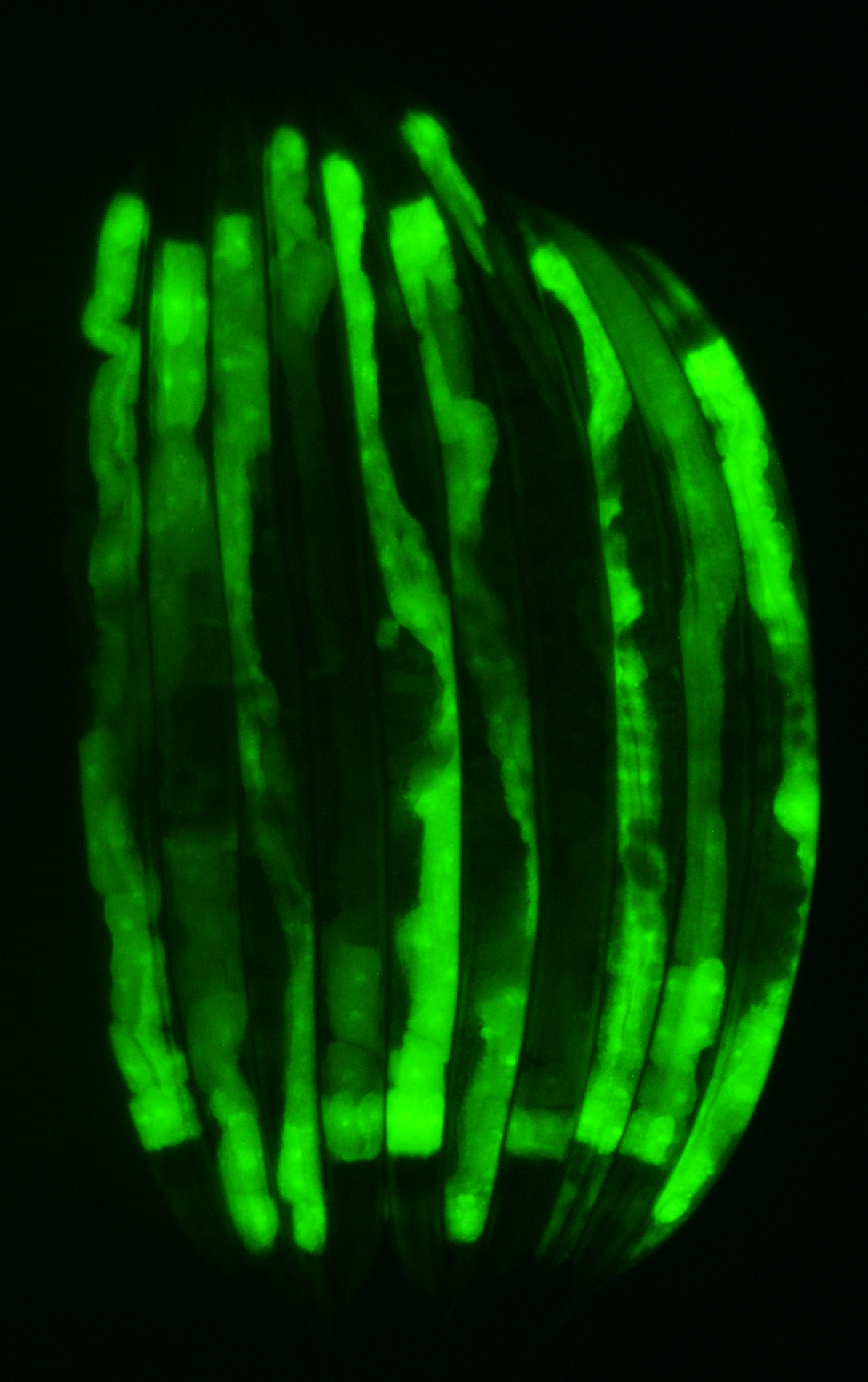

Supplement: Supplementary file 10 — Source data Fig. 4 [file 44318_2025_634_MOESM10_ESM.zip › Figure 4/Source data_Figure 4A/ΔybfB.tif]

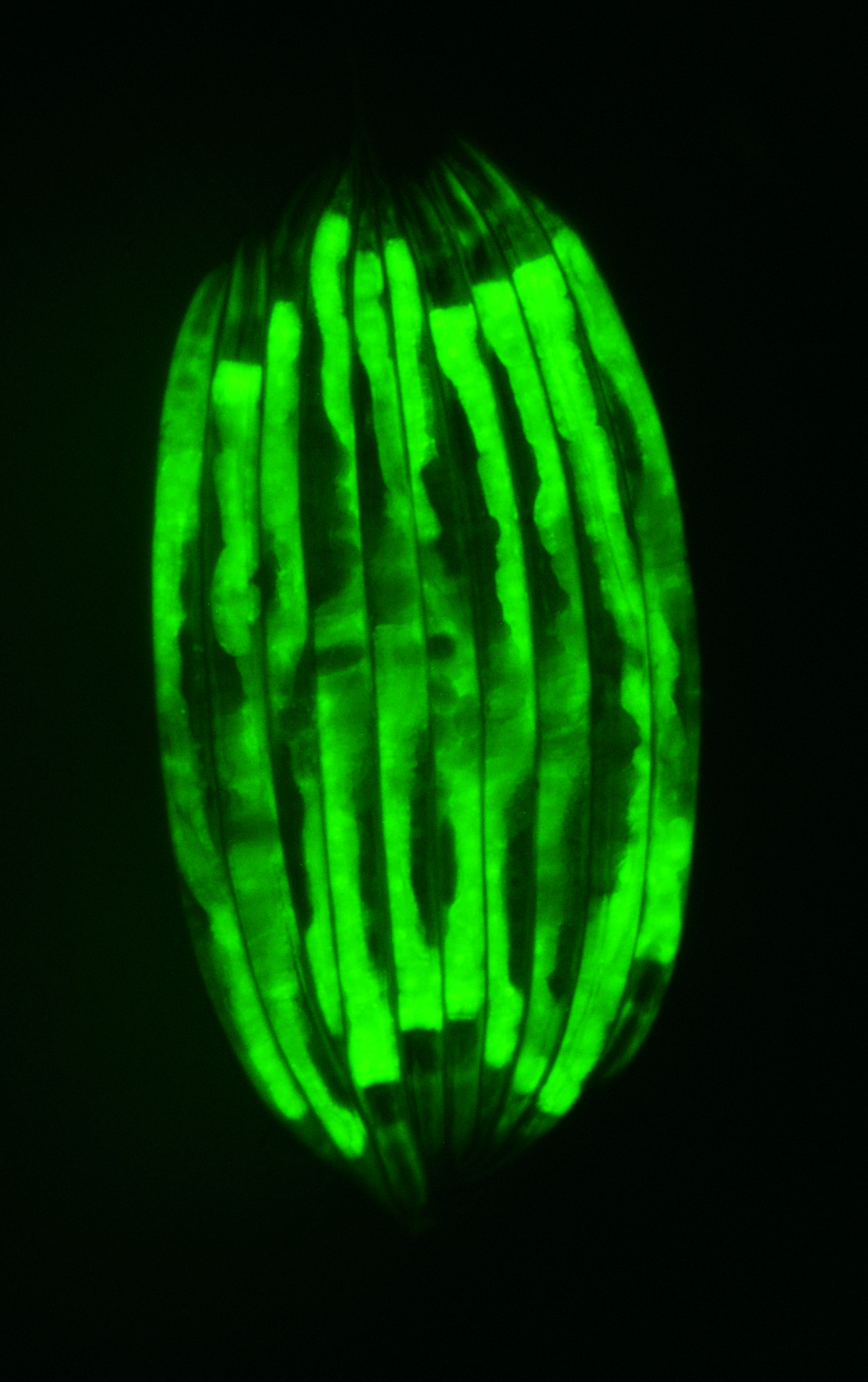

Supplement: Supplementary file 10 — Source data Fig. 4 [file 44318_2025_634_MOESM10_ESM.zip › Figure 4/Source data_Figure 4A/ΔycbK.tif]

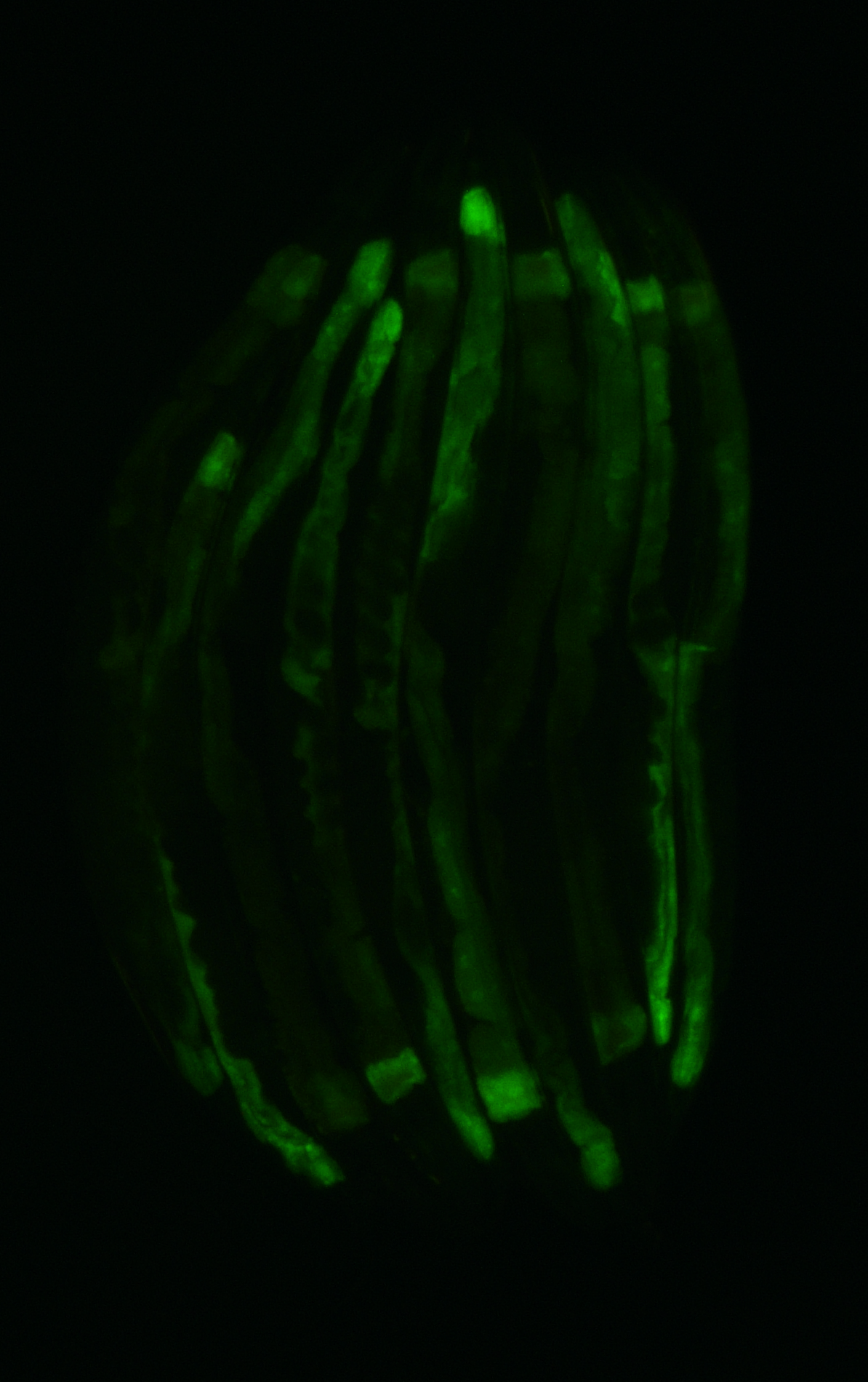

Supplement: Supplementary file 10 — Source data Fig. 4 [file 44318_2025_634_MOESM10_ESM.zip › Figure 4/Source data_Figure 4A/ΔyccA.tif]

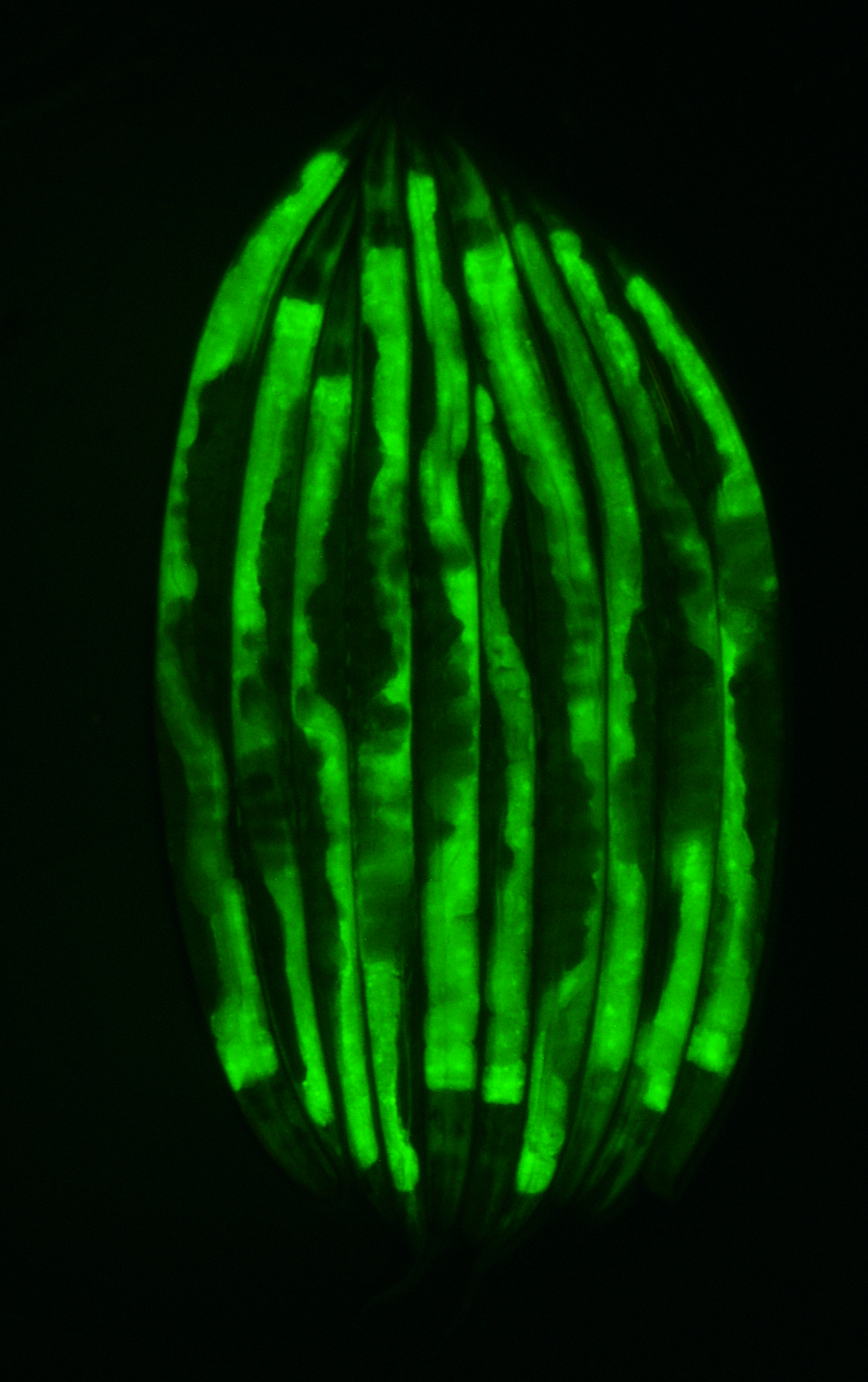

Supplement: Supplementary file 10 — Source data Fig. 4 [file 44318_2025_634_MOESM10_ESM.zip › Figure 4/Source data_Figure 4A/ΔyciA.tif]

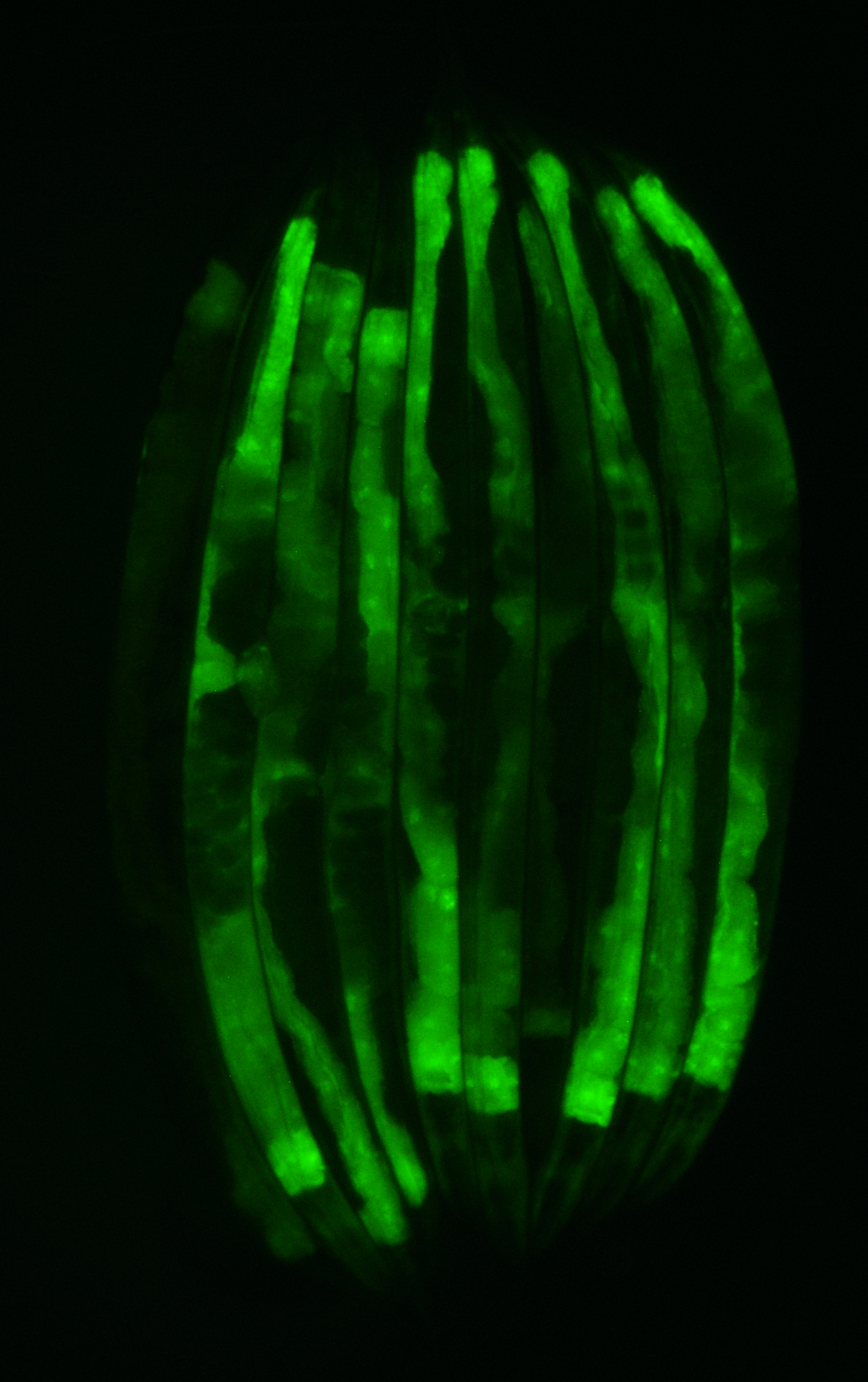

Supplement: Supplementary file 10 — Source data Fig. 4 [file 44318_2025_634_MOESM10_ESM.zip › Figure 4/Source data_Figure 4A/ΔyejG.tif]

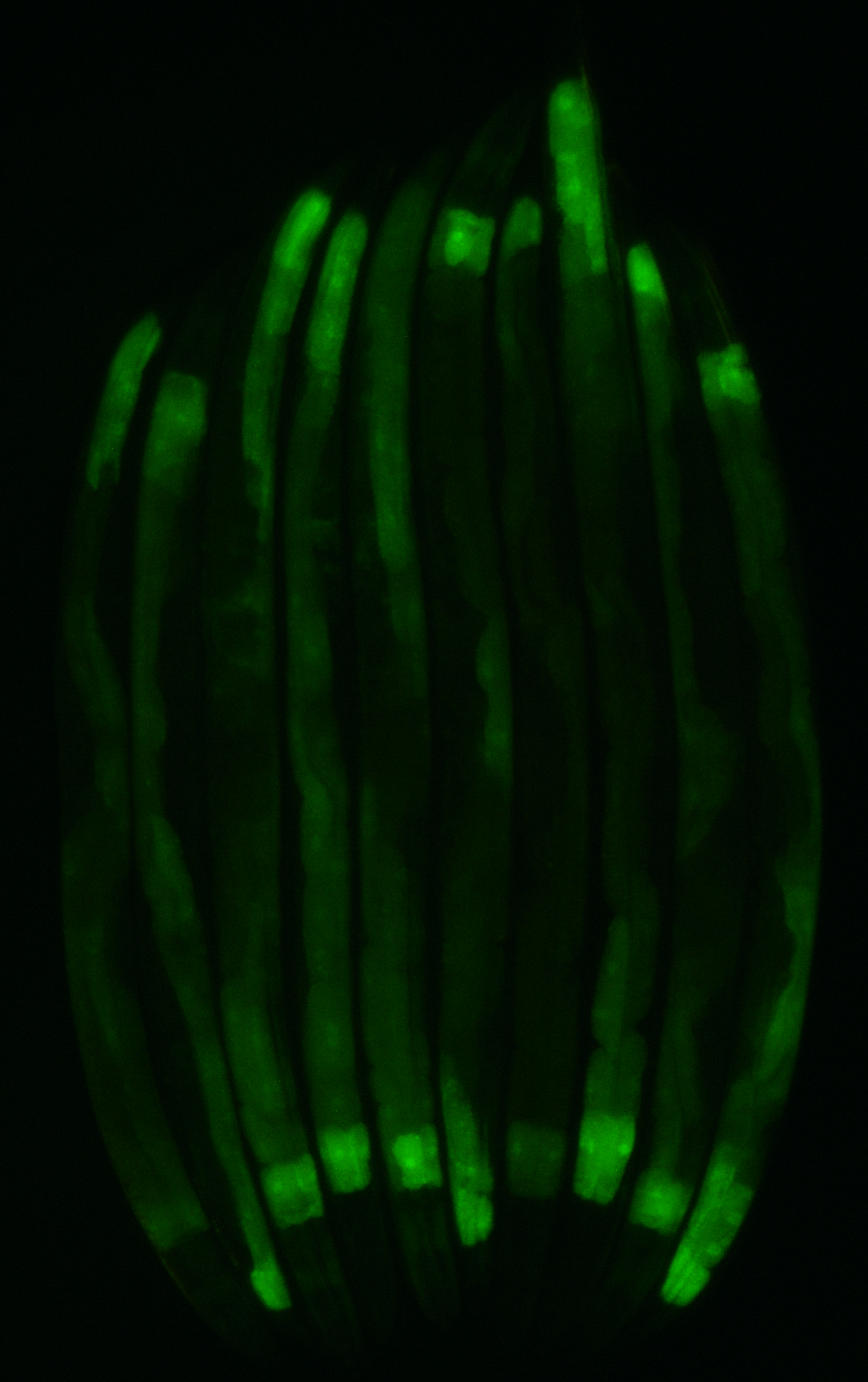

Supplement: Supplementary file 10 — Source data Fig. 4 [file 44318_2025_634_MOESM10_ESM.zip › Figure 4/Source data_Figure 4A/ΔyfaT.tif]

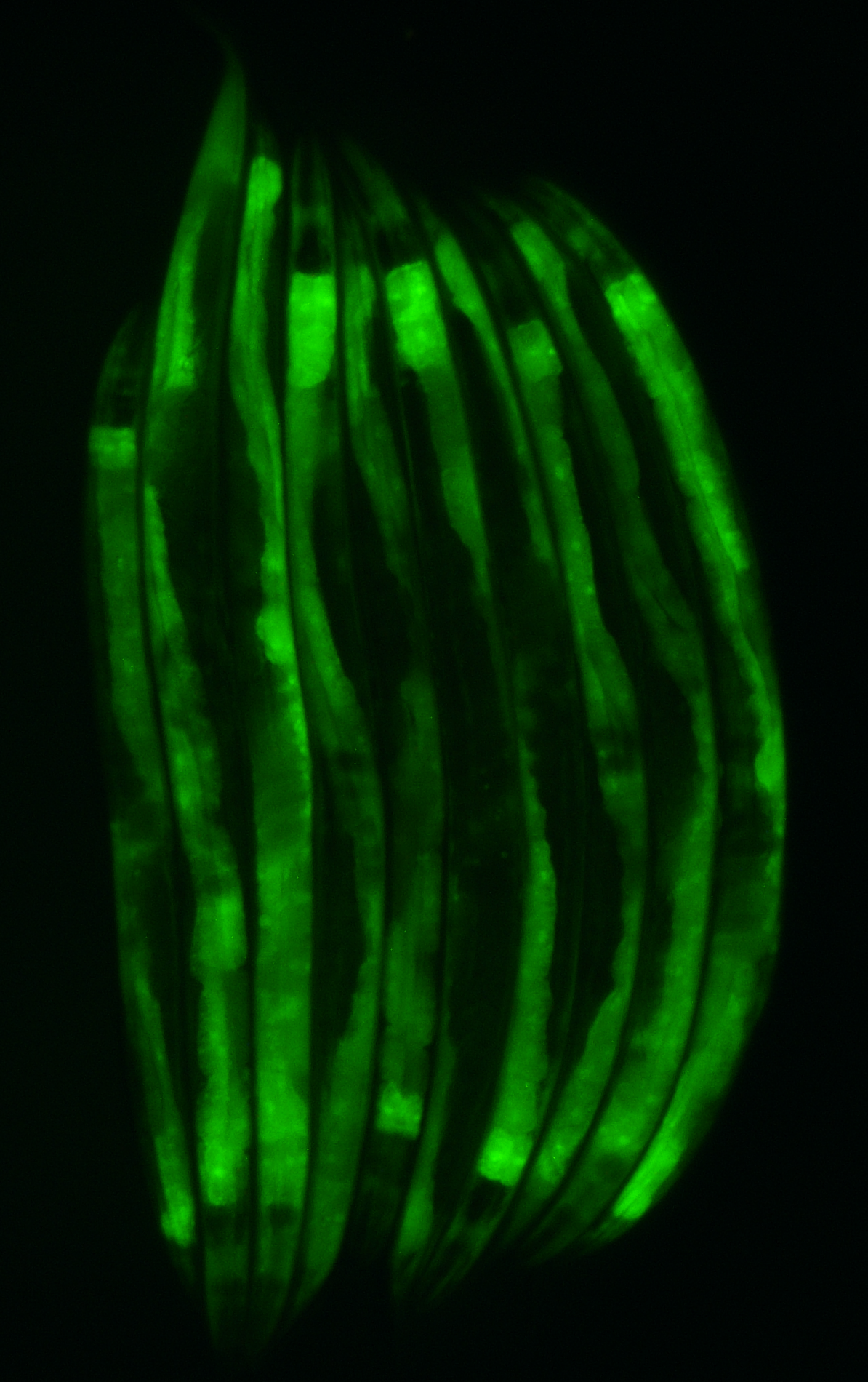

Supplement: Supplementary file 10 — Source data Fig. 4 [file 44318_2025_634_MOESM10_ESM.zip › Figure 4/Source data_Figure 4A/Δymfm.tif]

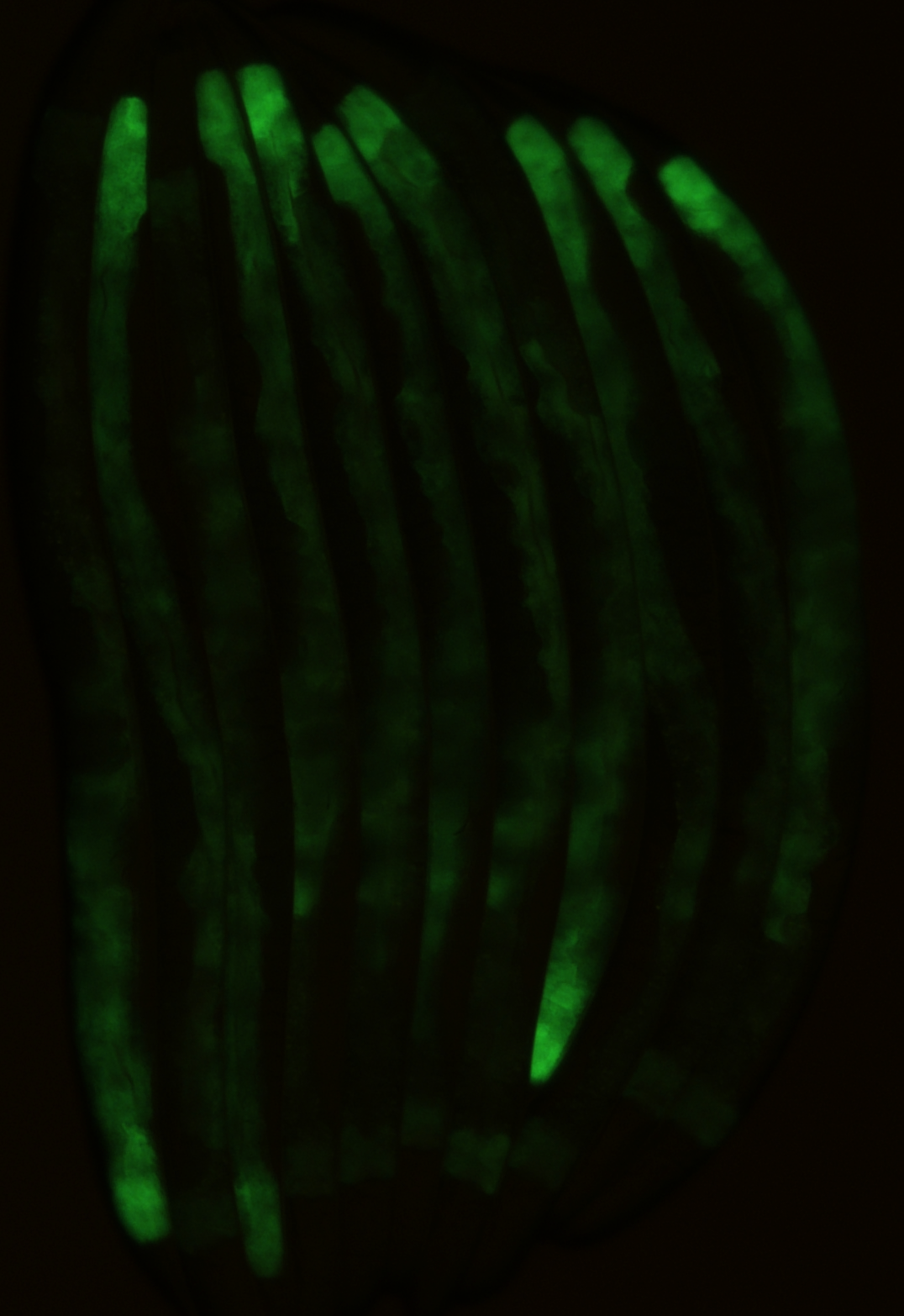

Supplement: Supplementary file 11 — Source data Fig. 5 [file 44318_2025_634_MOESM11_ESM.zip › Figure 5/Source data_Figure 5B/BW25113.tif]

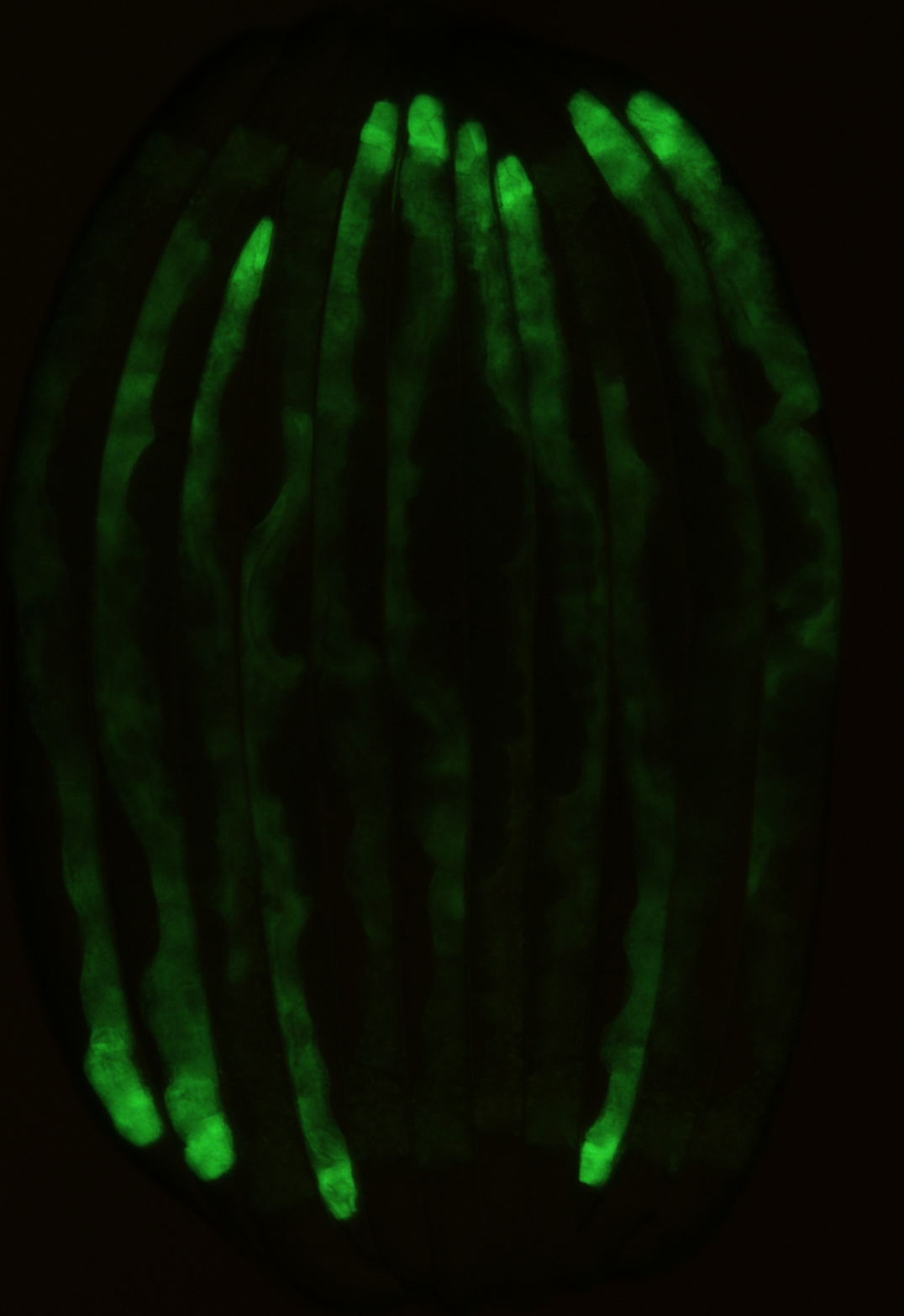

Supplement: Supplementary file 11 — Source data Fig. 5 [file 44318_2025_634_MOESM11_ESM.zip › Figure 5/Source data_Figure 5B/NAC BW25113.tif]

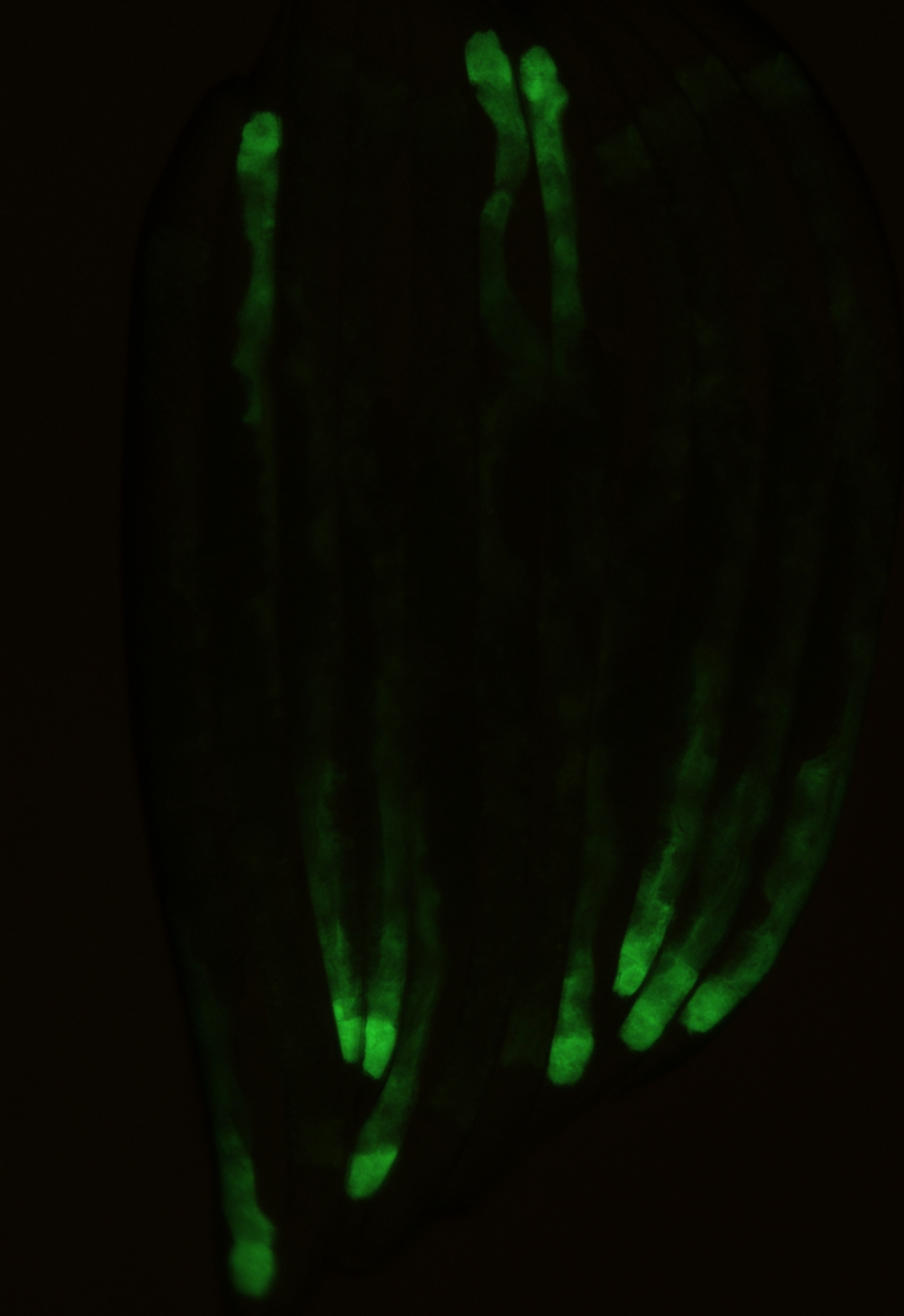

Supplement: Supplementary file 11 — Source data Fig. 5 [file 44318_2025_634_MOESM11_ESM.zip › Figure 5/Source data_Figure 5B/NAC ΔallD.tif]

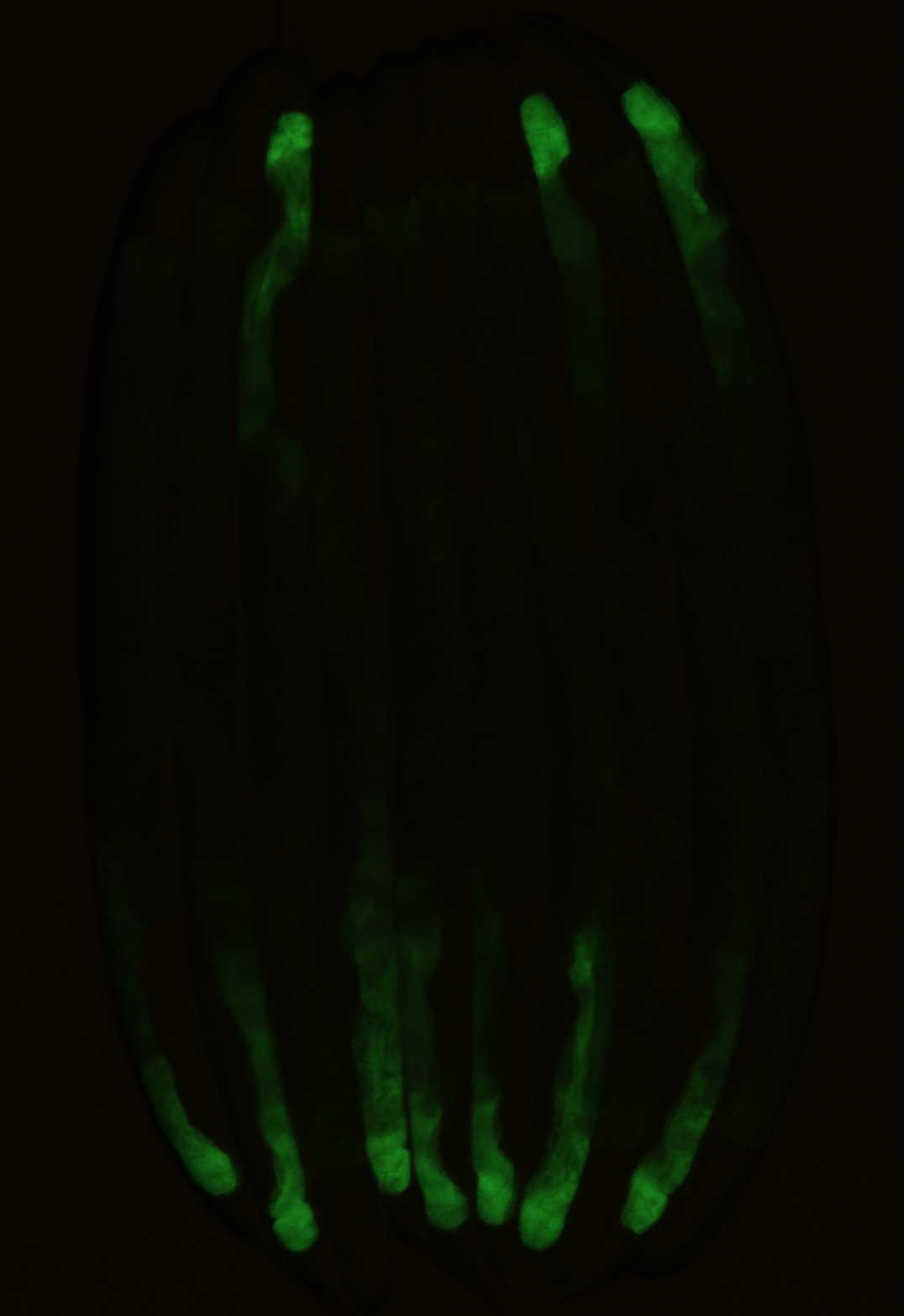

Supplement: Supplementary file 11 — Source data Fig. 5 [file 44318_2025_634_MOESM11_ESM.zip › Figure 5/Source data_Figure 5B/NAC ΔpdeI.tif]

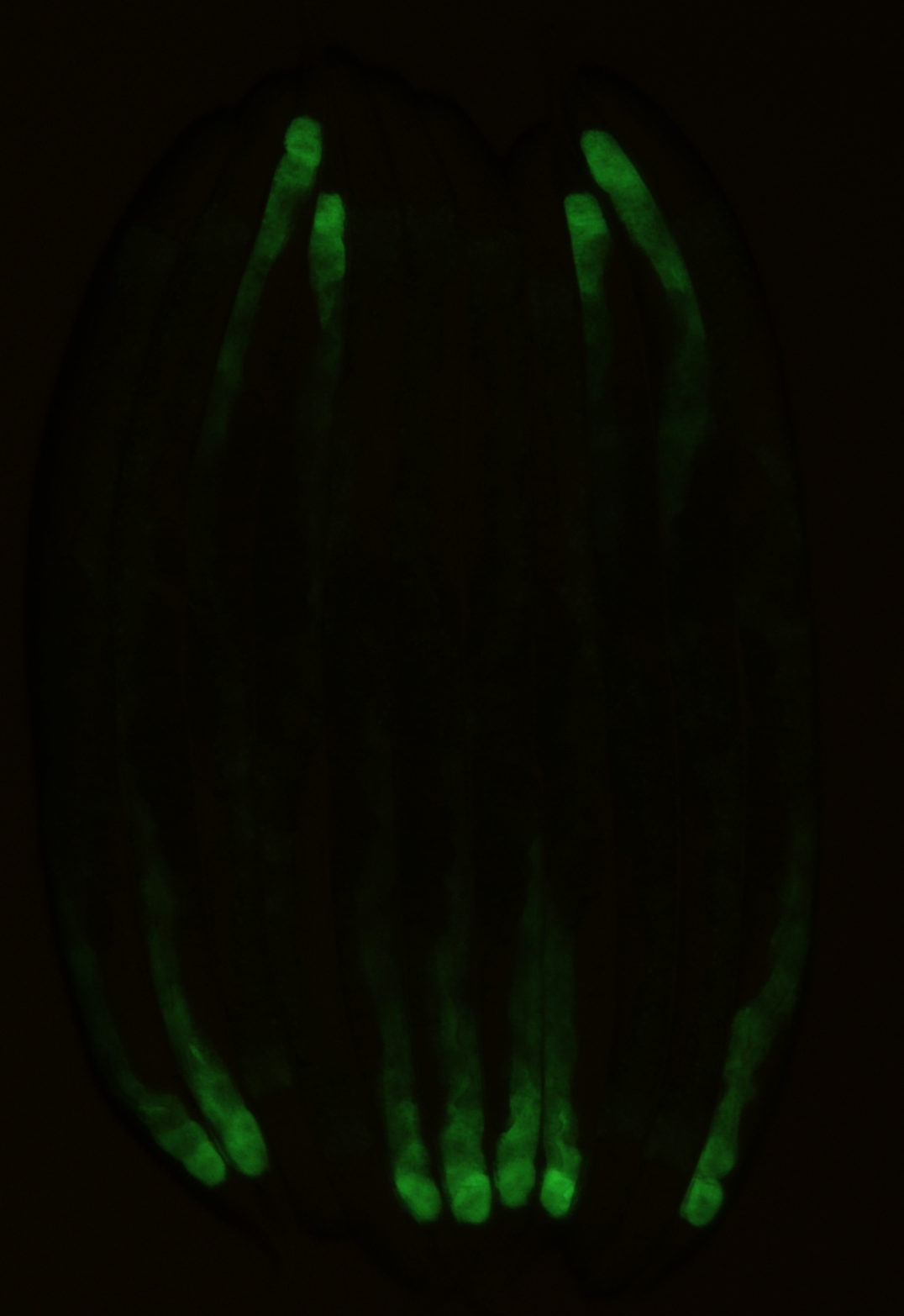

Supplement: Supplementary file 11 — Source data Fig. 5 [file 44318_2025_634_MOESM11_ESM.zip › Figure 5/Source data_Figure 5B/NAC ΔtktA.tif]

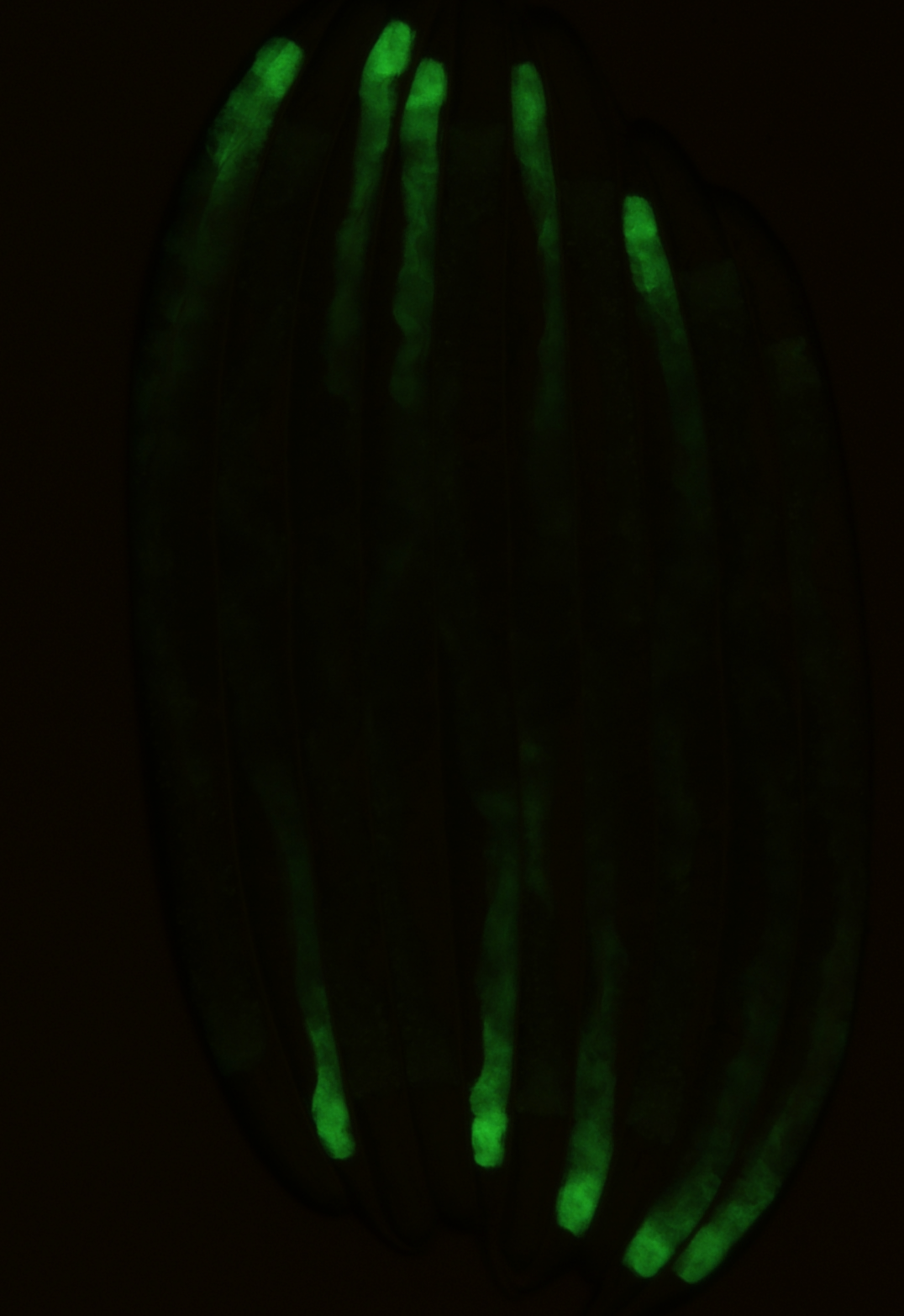

Supplement: Supplementary file 11 — Source data Fig. 5 [file 44318_2025_634_MOESM11_ESM.zip › Figure 5/Source data_Figure 5B/NAC ΔyciA.tif]

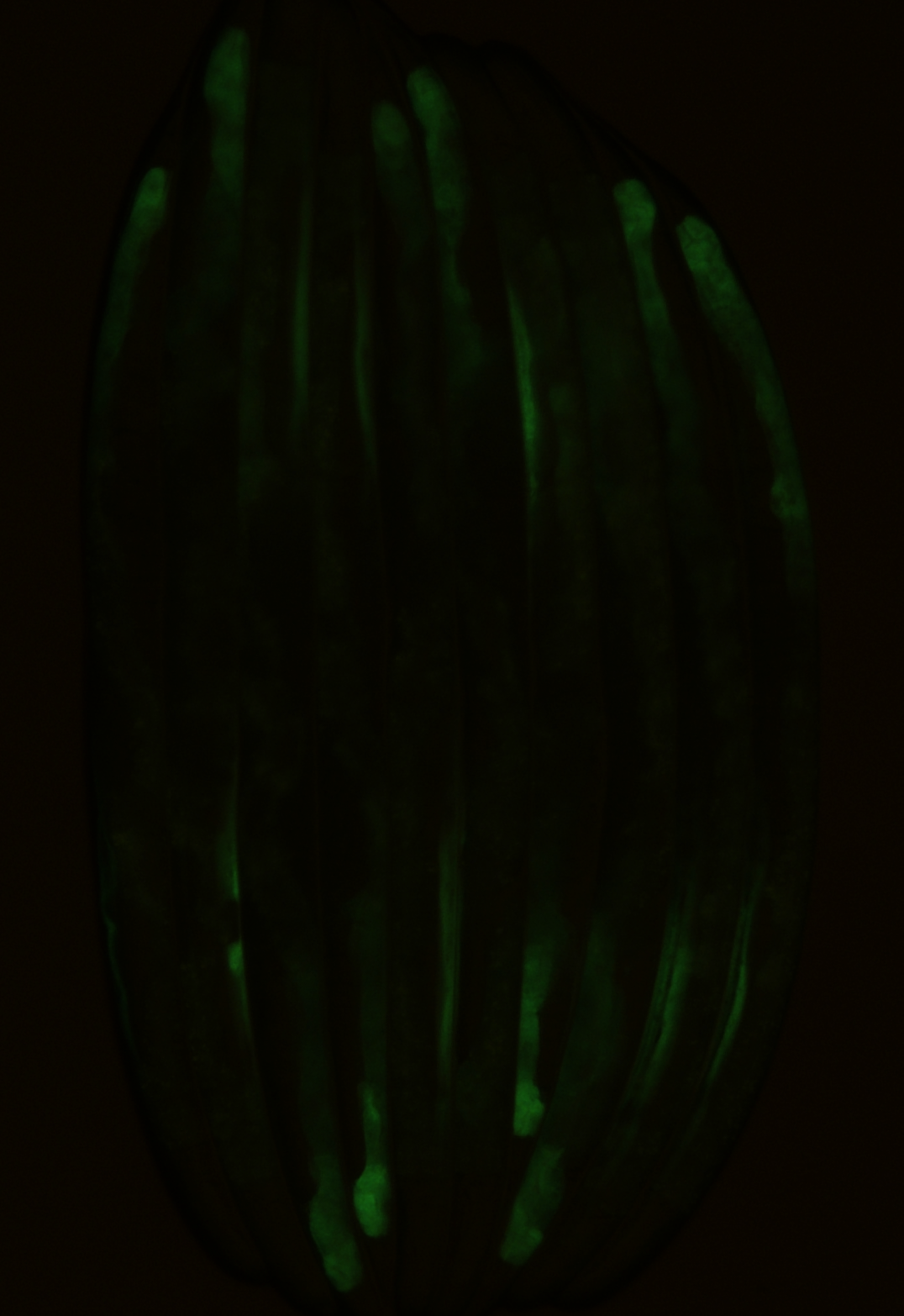

Supplement: Supplementary file 11 — Source data Fig. 5 [file 44318_2025_634_MOESM11_ESM.zip › Figure 5/Source data_Figure 5B/ΔallD.tif]

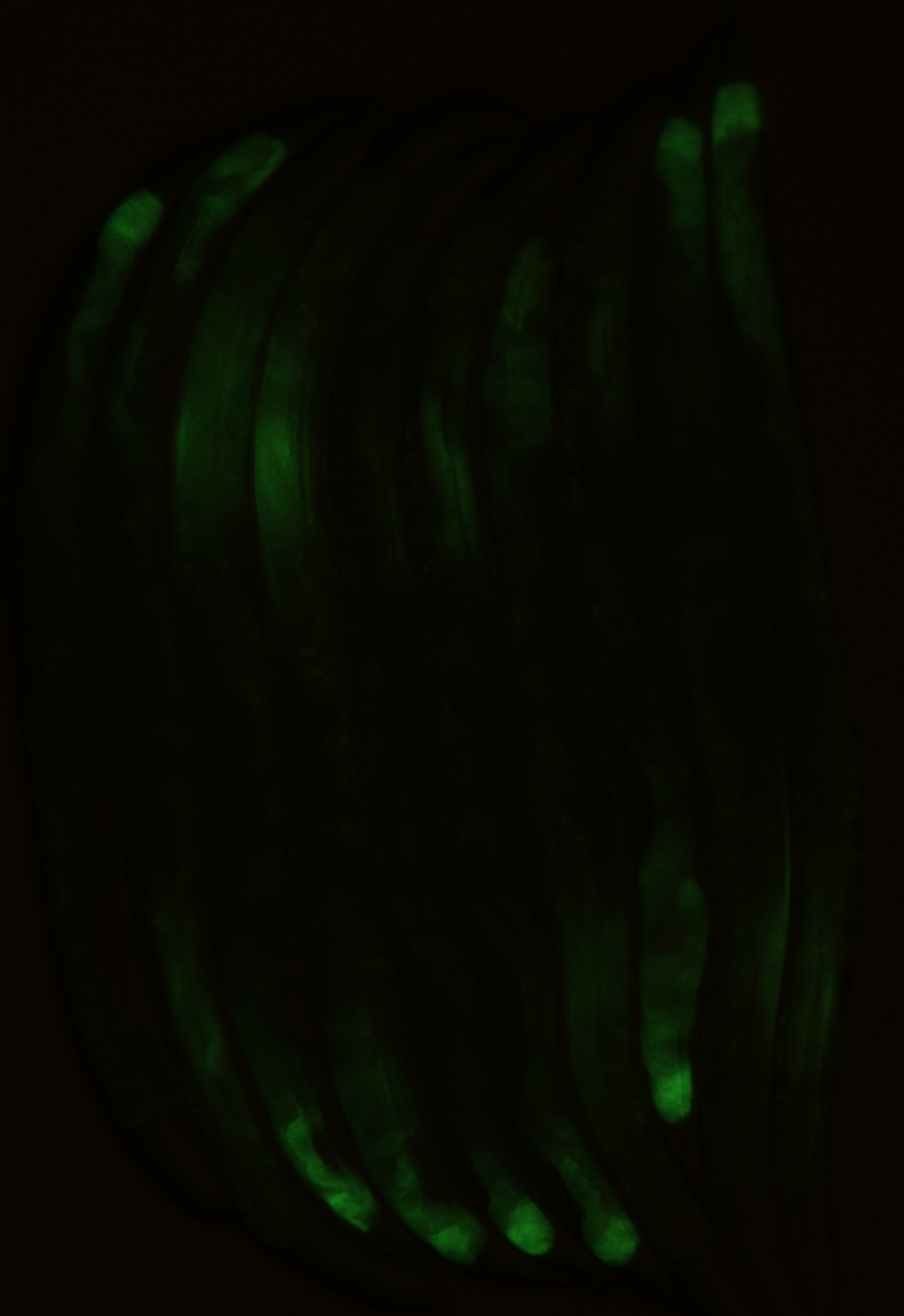

Supplement: Supplementary file 11 — Source data Fig. 5 [file 44318_2025_634_MOESM11_ESM.zip › Figure 5/Source data_Figure 5B/ΔpdeI.tif]

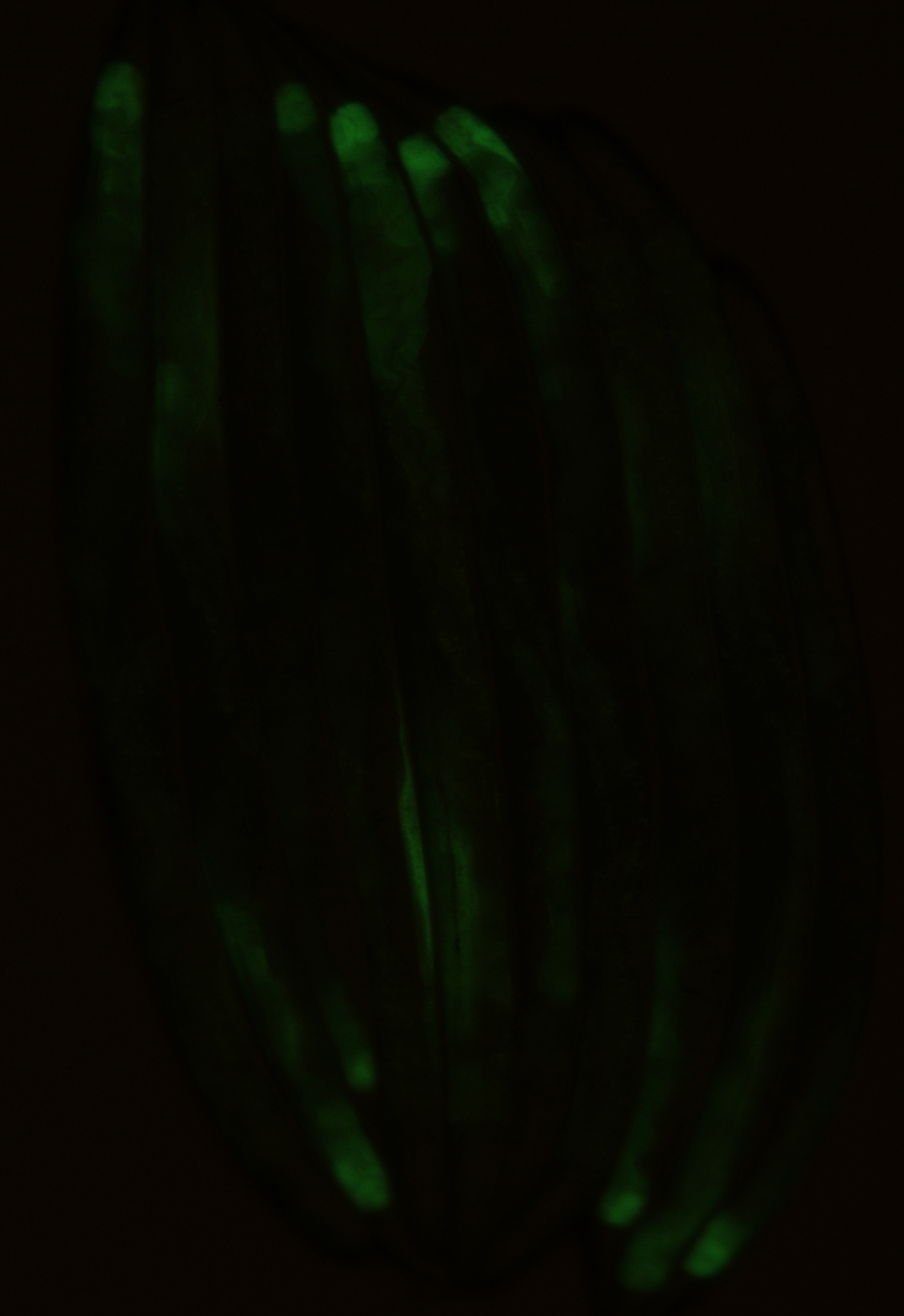

Supplement: Supplementary file 11 — Source data Fig. 5 [file 44318_2025_634_MOESM11_ESM.zip › Figure 5/Source data_Figure 5B/ΔtktA.tif]

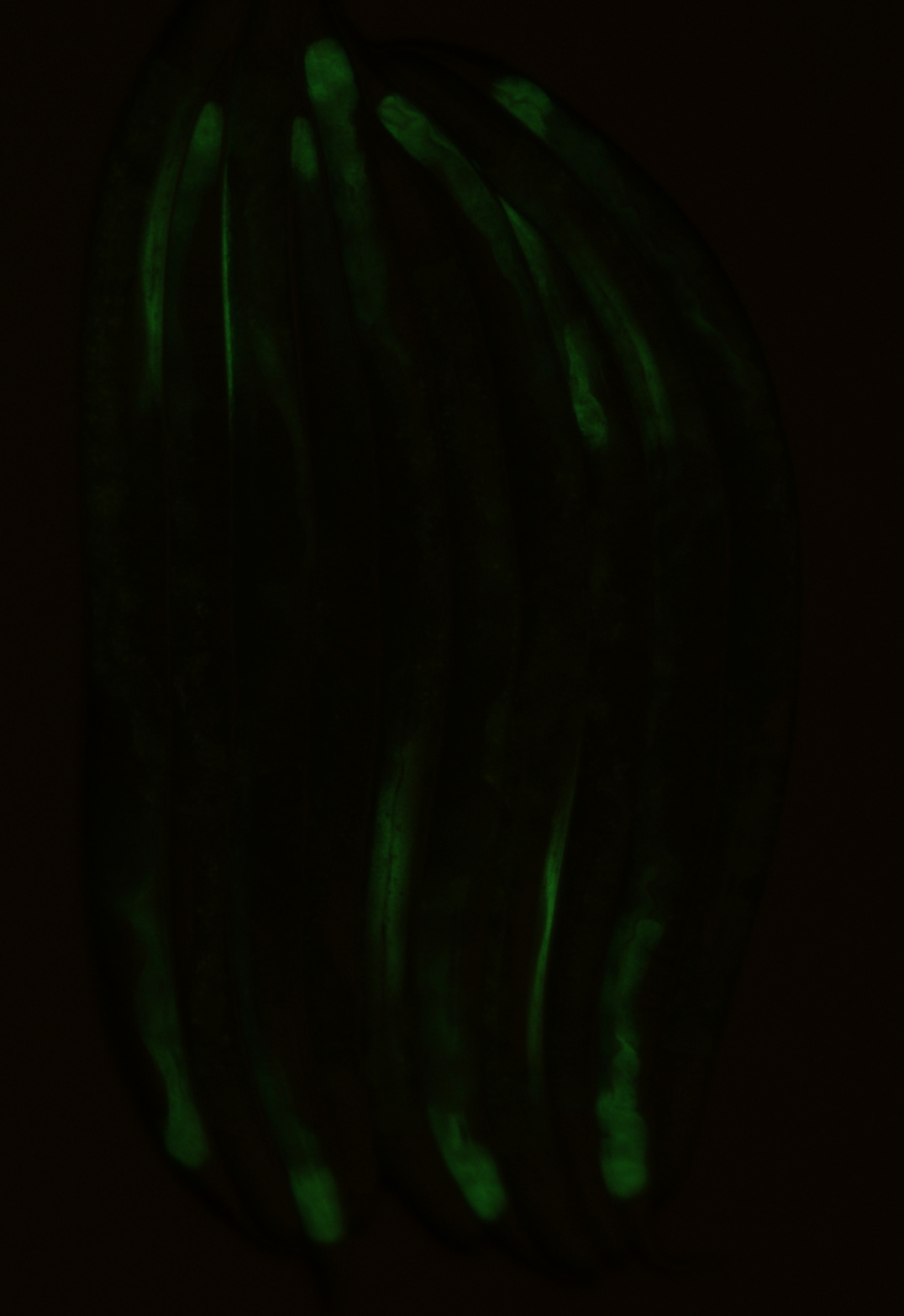

Supplement: Supplementary file 11 — Source data Fig. 5 [file 44318_2025_634_MOESM11_ESM.zip › Figure 5/Source data_Figure 5B/ΔyciA.tif]

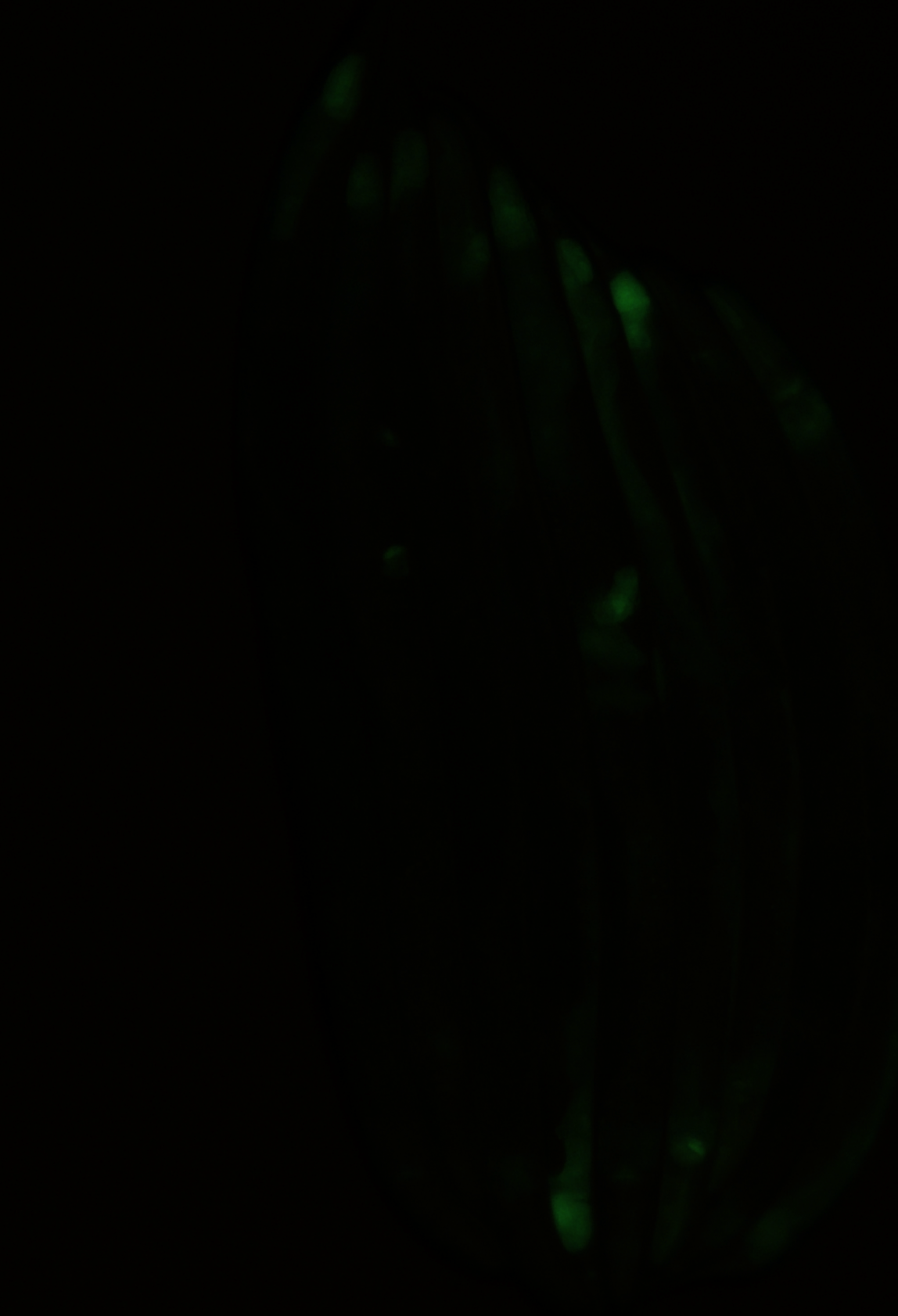

Supplement: Supplementary file 11 — Source data Fig. 5 [file 44318_2025_634_MOESM11_ESM.zip › Figure 5/Source data_Figure 5D/BW25113.tif]

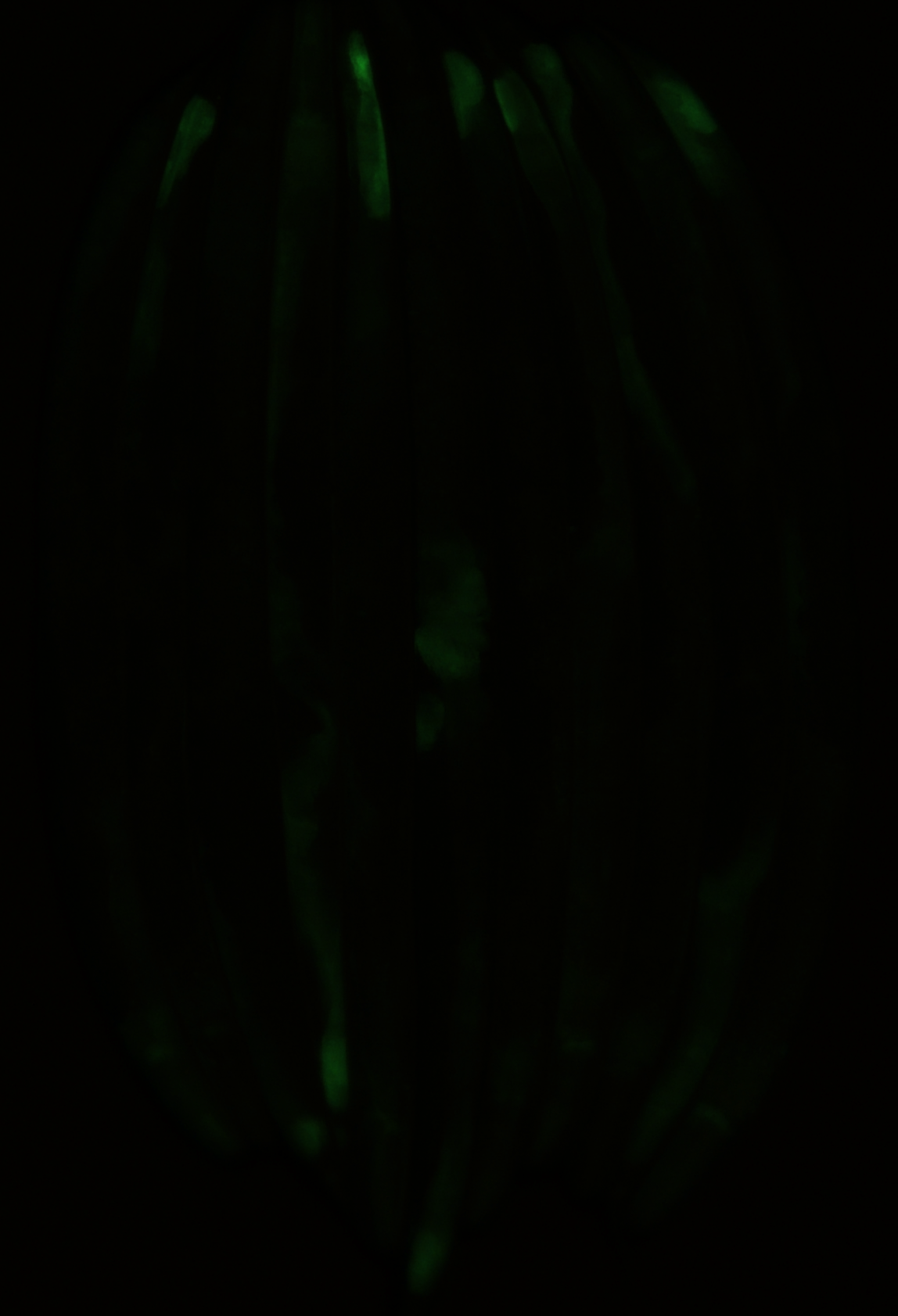

Supplement: Supplementary file 11 — Source data Fig. 5 [file 44318_2025_634_MOESM11_ESM.zip › Figure 5/Source data_Figure 5D/NAC BW25113.tif]

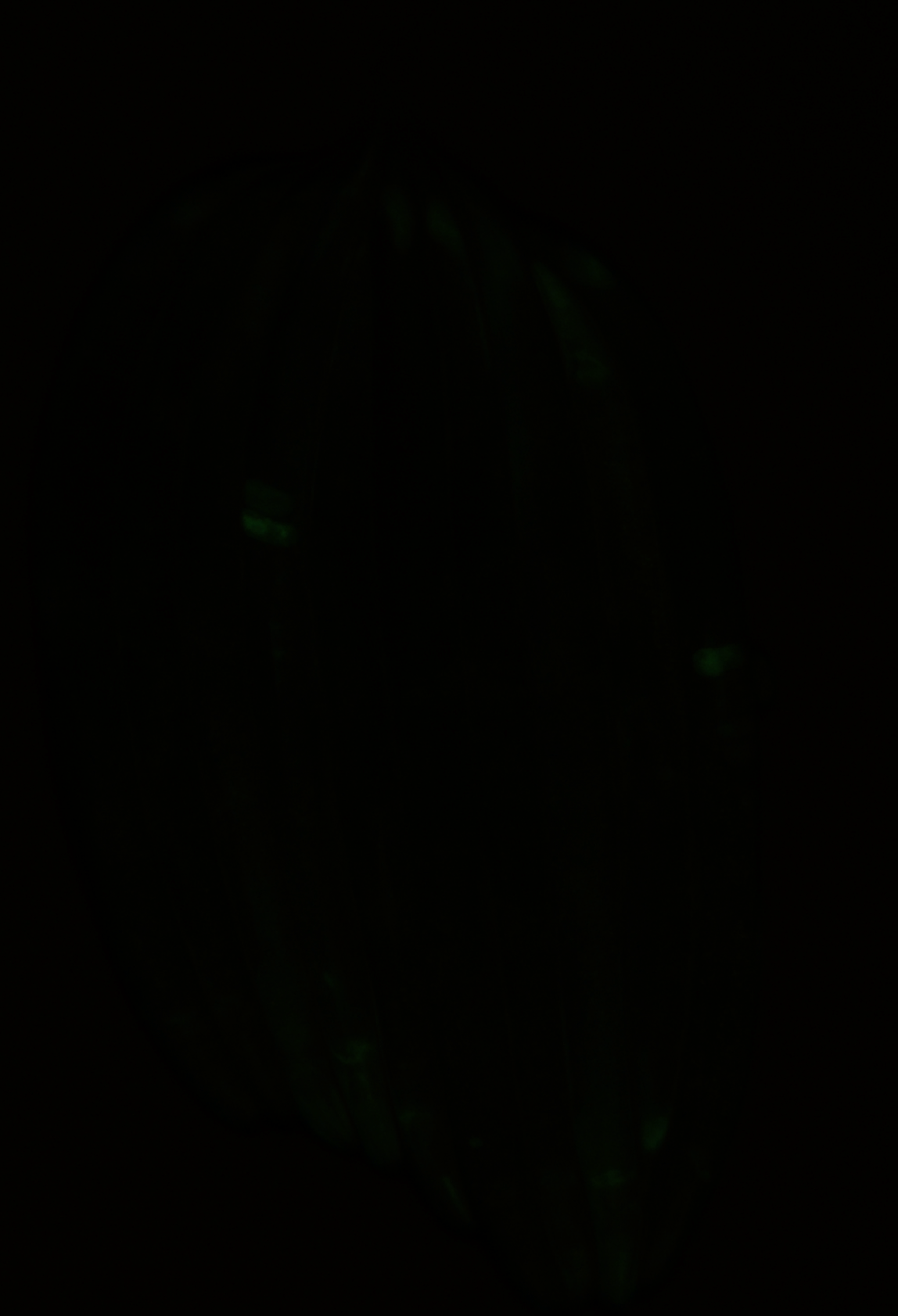

Supplement: Supplementary file 11 — Source data Fig. 5 [file 44318_2025_634_MOESM11_ESM.zip › Figure 5/Source data_Figure 5D/NAC ΔallD.tif]

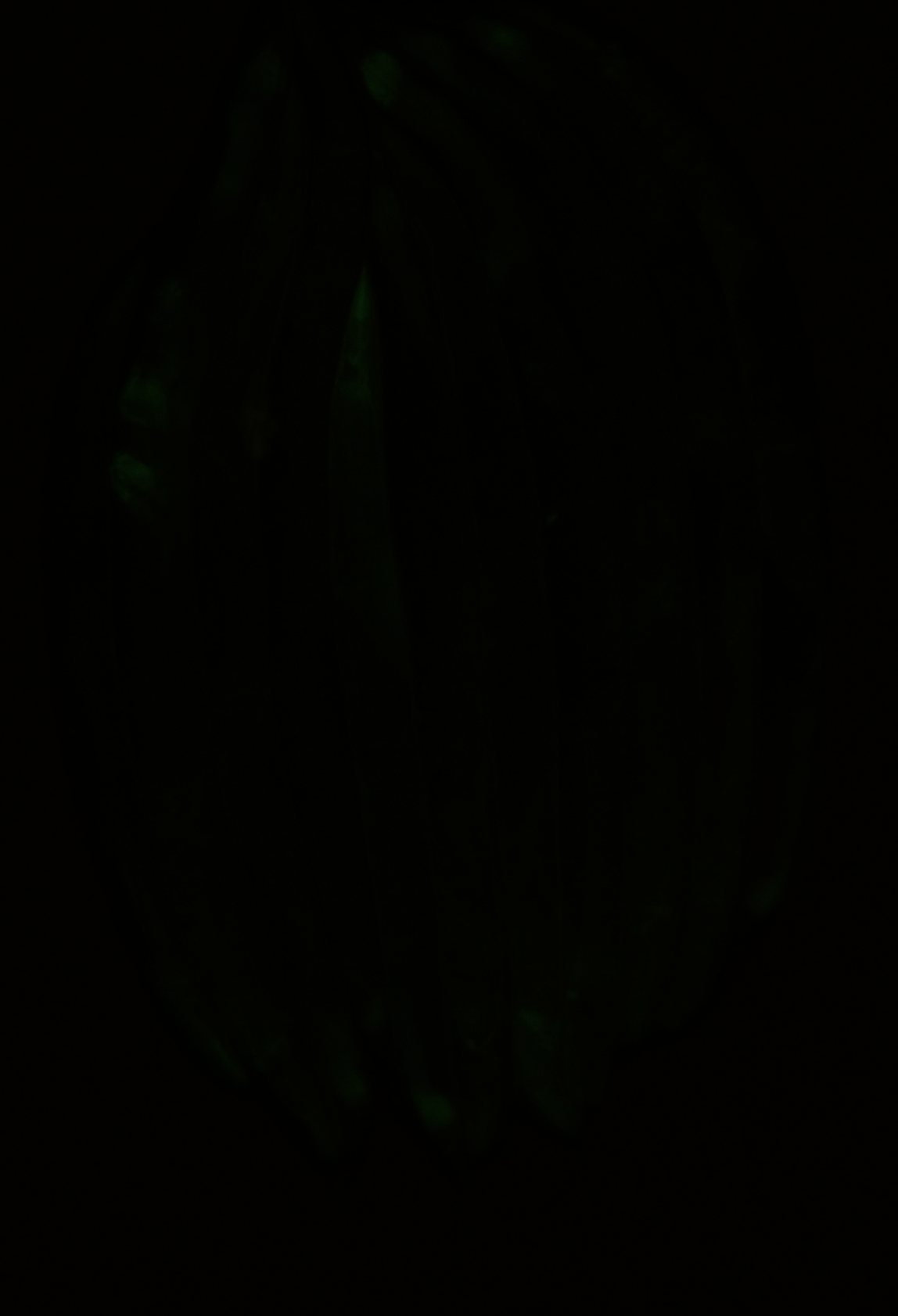

Supplement: Supplementary file 11 — Source data Fig. 5 [file 44318_2025_634_MOESM11_ESM.zip › Figure 5/Source data_Figure 5D/NAC ΔpdeI.tif]

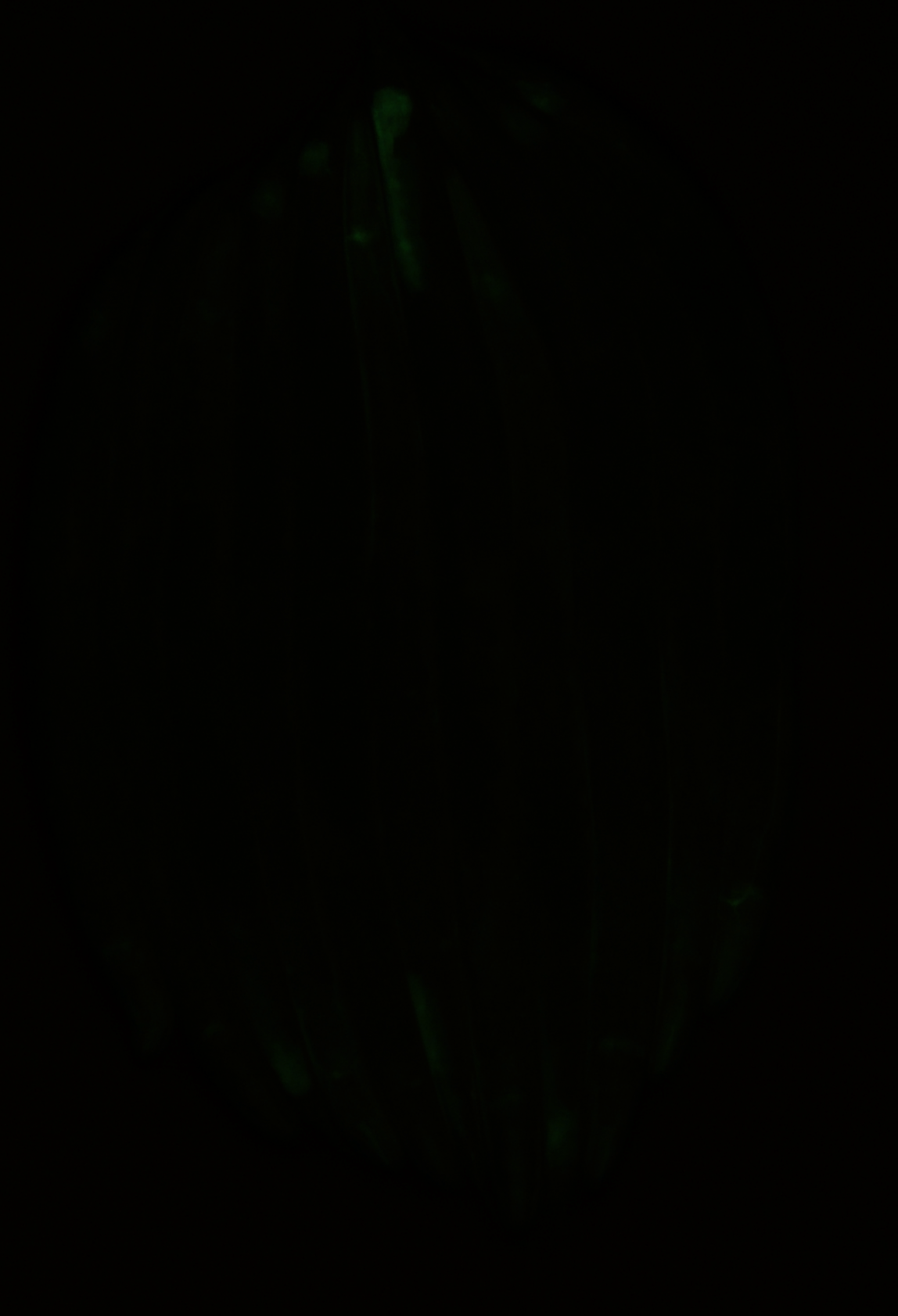

Supplement: Supplementary file 11 — Source data Fig. 5 [file 44318_2025_634_MOESM11_ESM.zip › Figure 5/Source data_Figure 5D/NAC ΔtktA.tif]

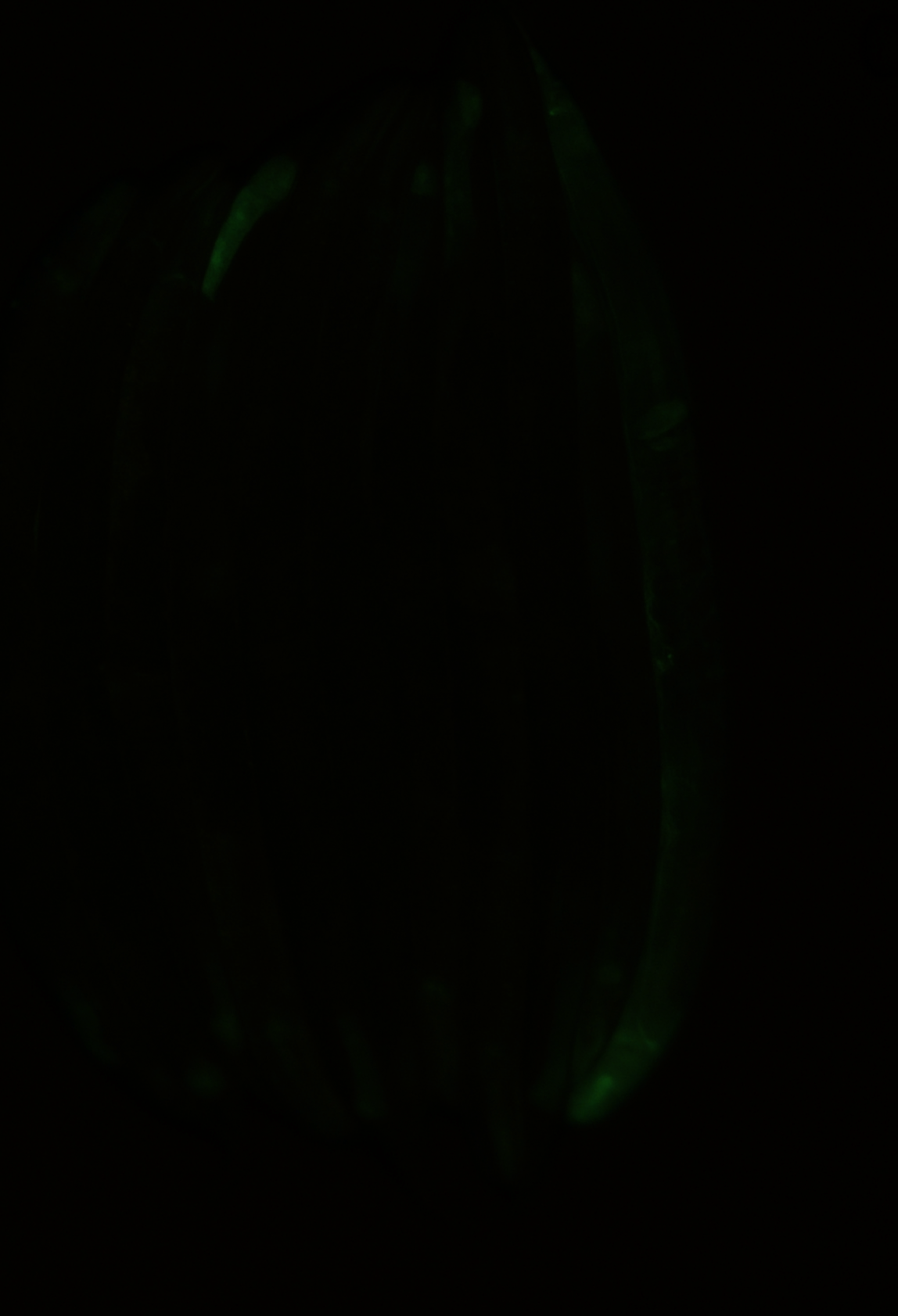

Supplement: Supplementary file 11 — Source data Fig. 5 [file 44318_2025_634_MOESM11_ESM.zip › Figure 5/Source data_Figure 5D/NAC ΔyciA.tif]

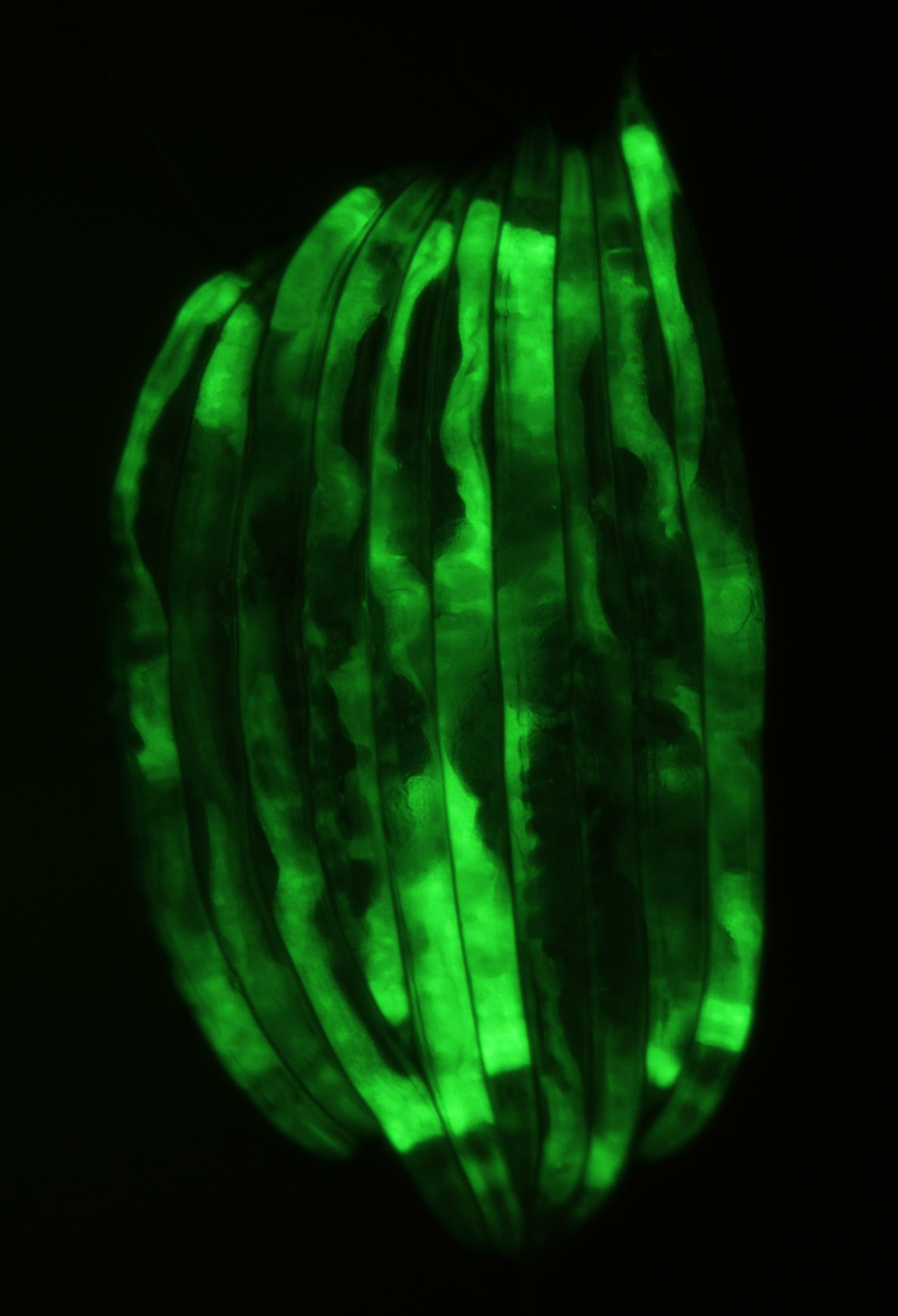

Supplement: Supplementary file 11 — Source data Fig. 5 [file 44318_2025_634_MOESM11_ESM.zip › Figure 5/Source data_Figure 5D/ΔallD.tif]

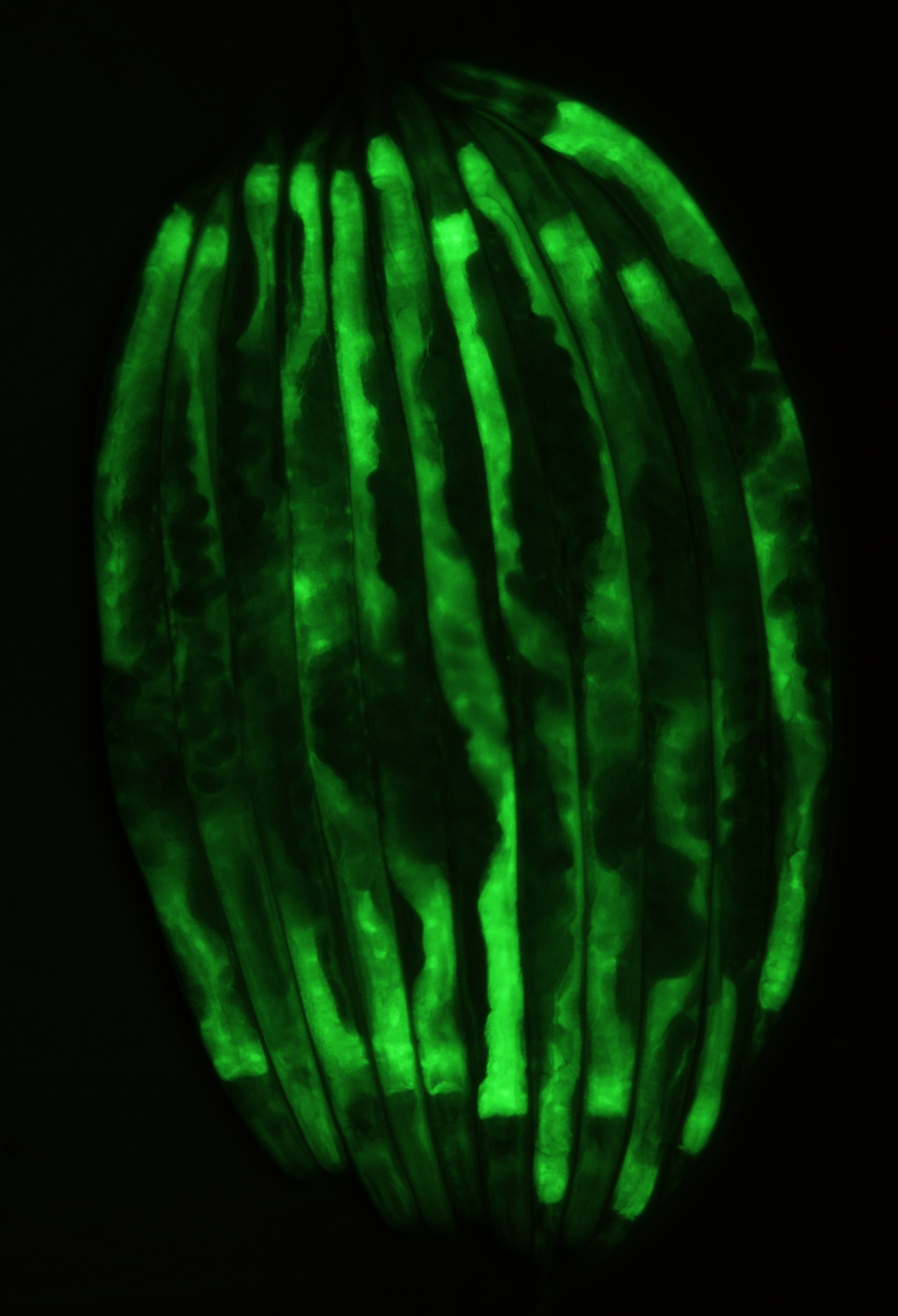

Supplement: Supplementary file 11 — Source data Fig. 5 [file 44318_2025_634_MOESM11_ESM.zip › Figure 5/Source data_Figure 5D/ΔpdeI.tif]

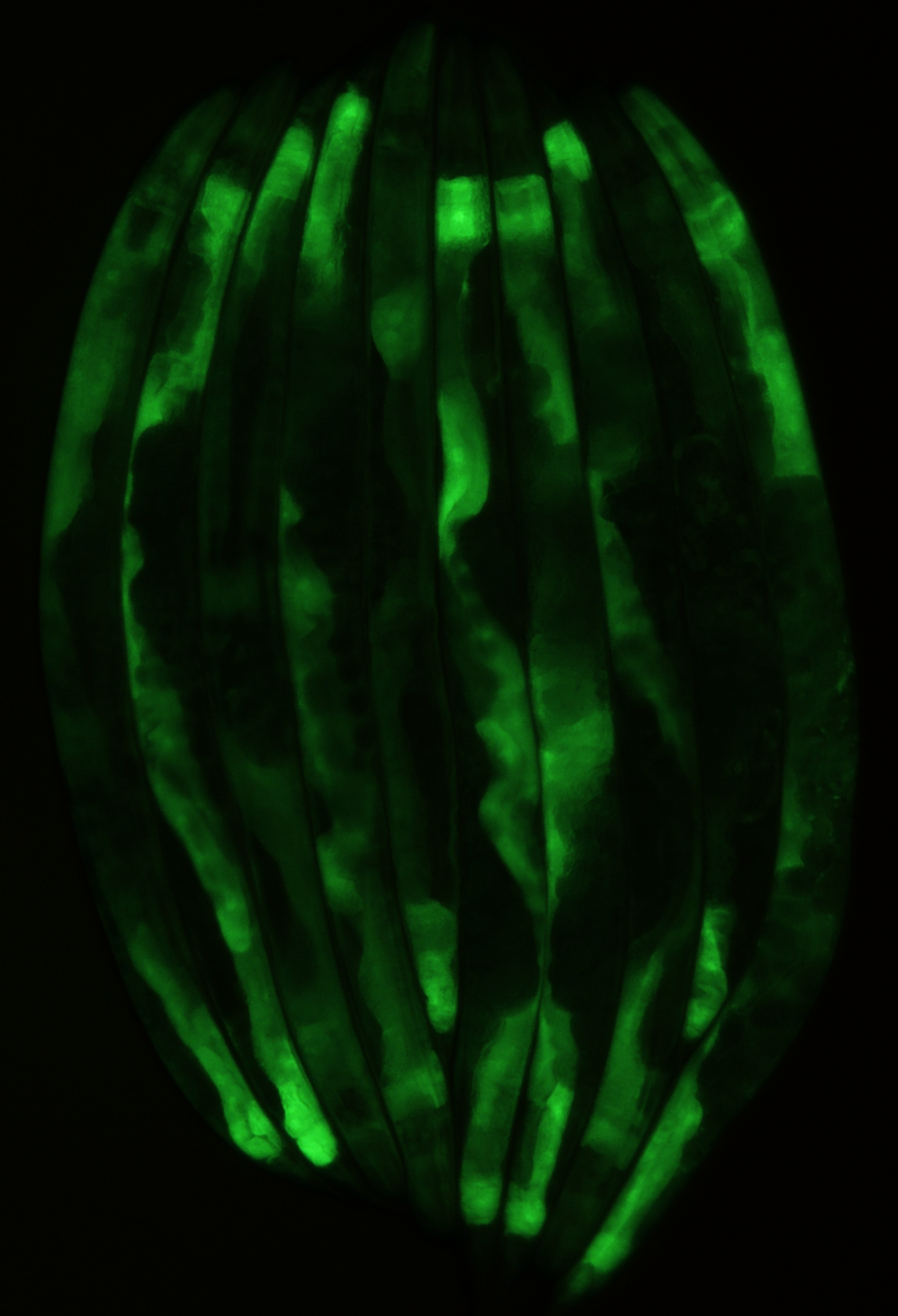

Supplement: Supplementary file 11 — Source data Fig. 5 [file 44318_2025_634_MOESM11_ESM.zip › Figure 5/Source data_Figure 5D/ΔtktA.tif]

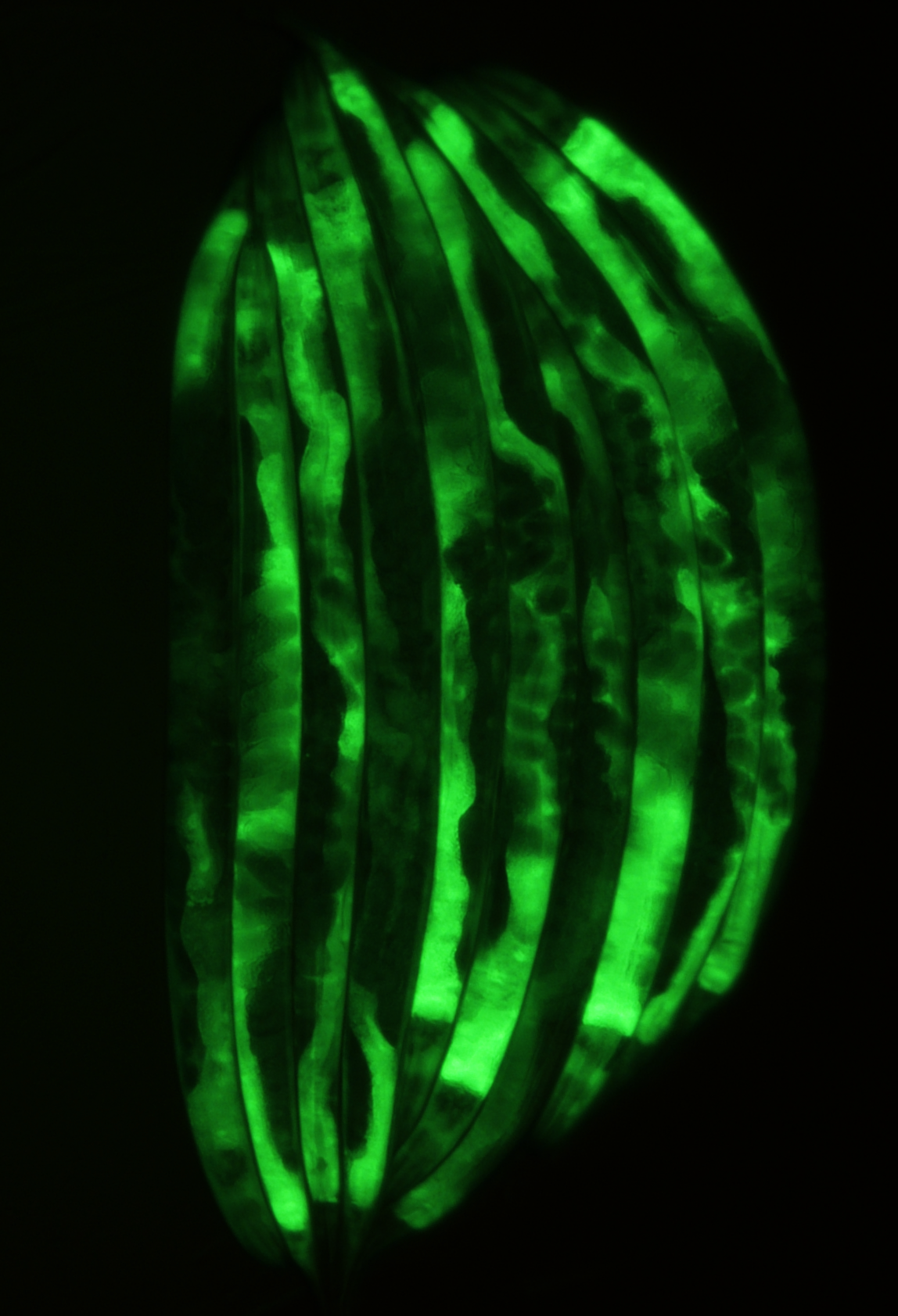

Supplement: Supplementary file 11 — Source data Fig. 5 [file 44318_2025_634_MOESM11_ESM.zip › Figure 5/Source data_Figure 5D/ΔyciA.tif]

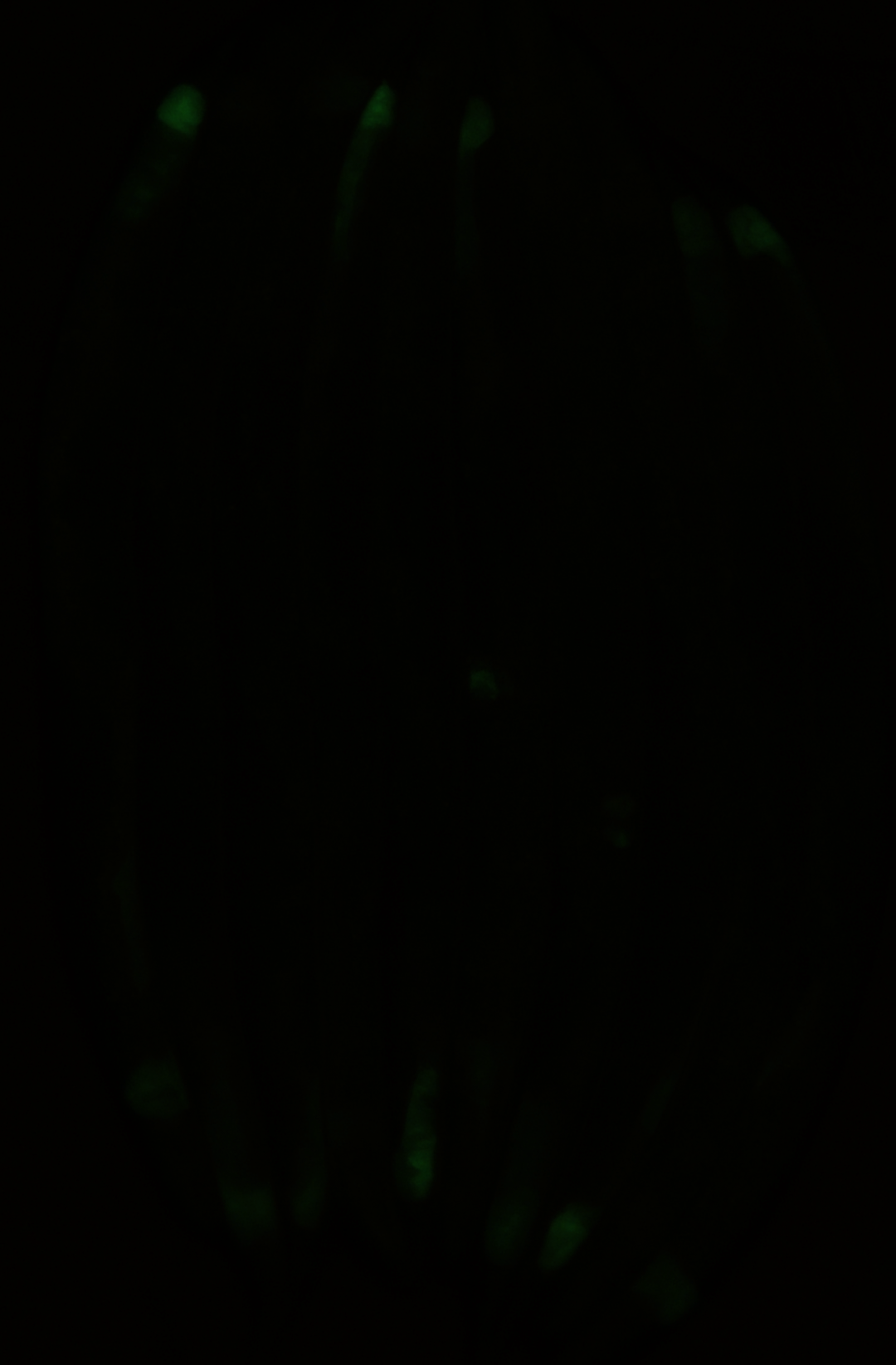

Supplement: Supplementary file 12 — Source data Fig. 6 [file 44318_2025_634_MOESM12_ESM.zip › Figure 6/Source data_Figure 6B/BW25113.tif]

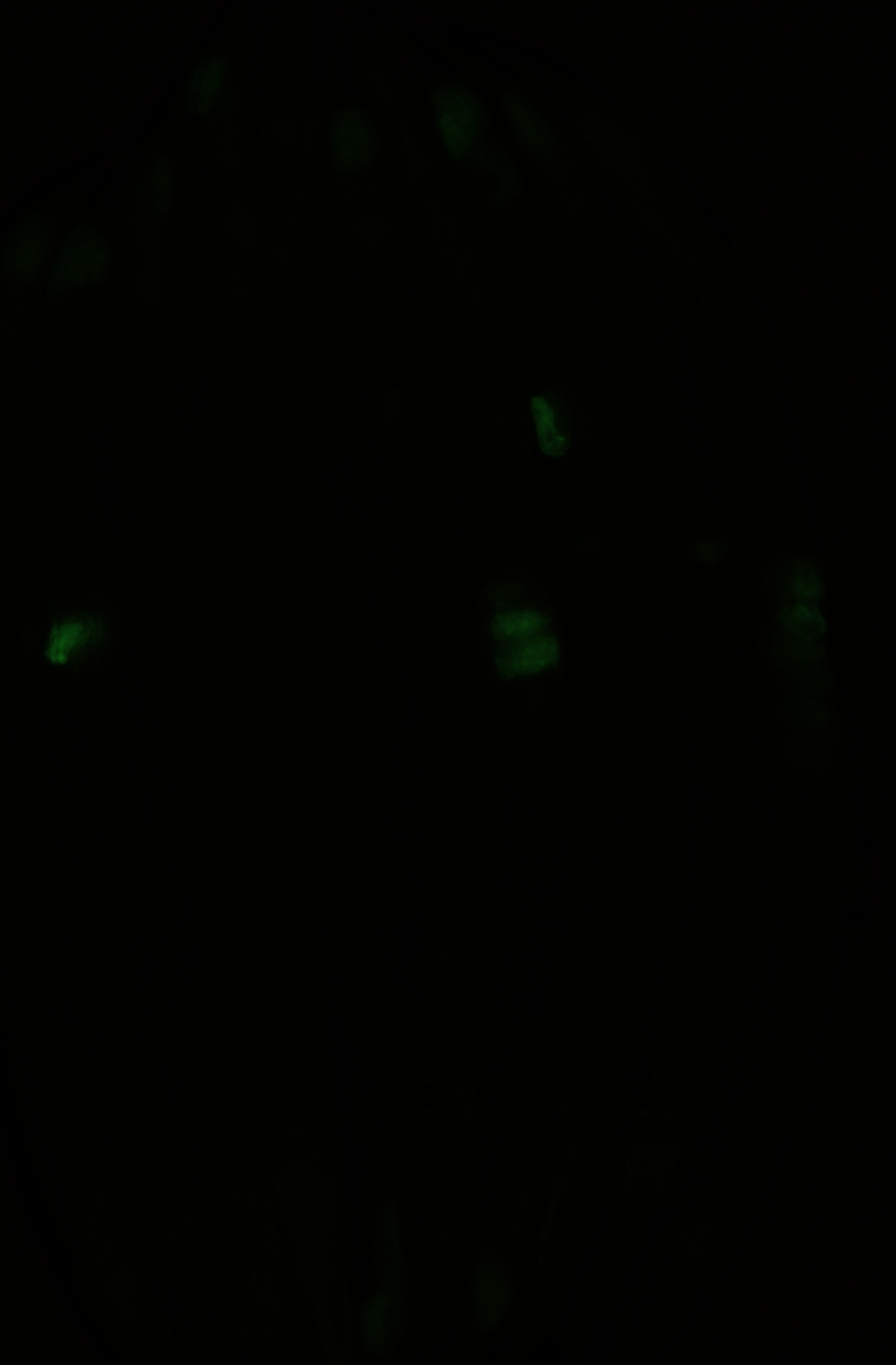

Supplement: Supplementary file 12 — Source data Fig. 6 [file 44318_2025_634_MOESM12_ESM.zip › Figure 6/Source data_Figure 6B/Fecl3 BW25113.tif]

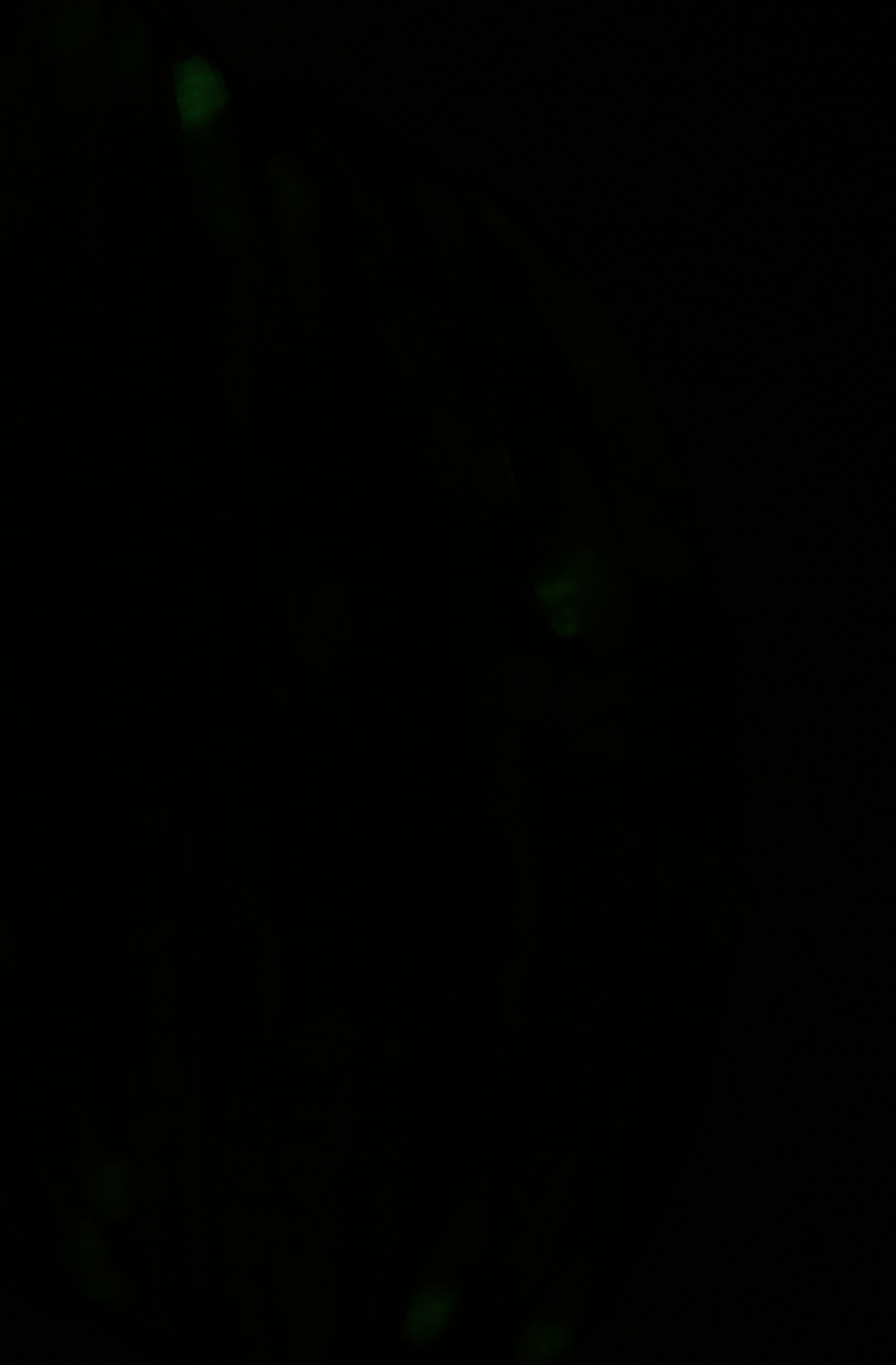

Supplement: Supplementary file 12 — Source data Fig. 6 [file 44318_2025_634_MOESM12_ESM.zip › Figure 6/Source data_Figure 6B/Fecl3 ΔallD.tif]

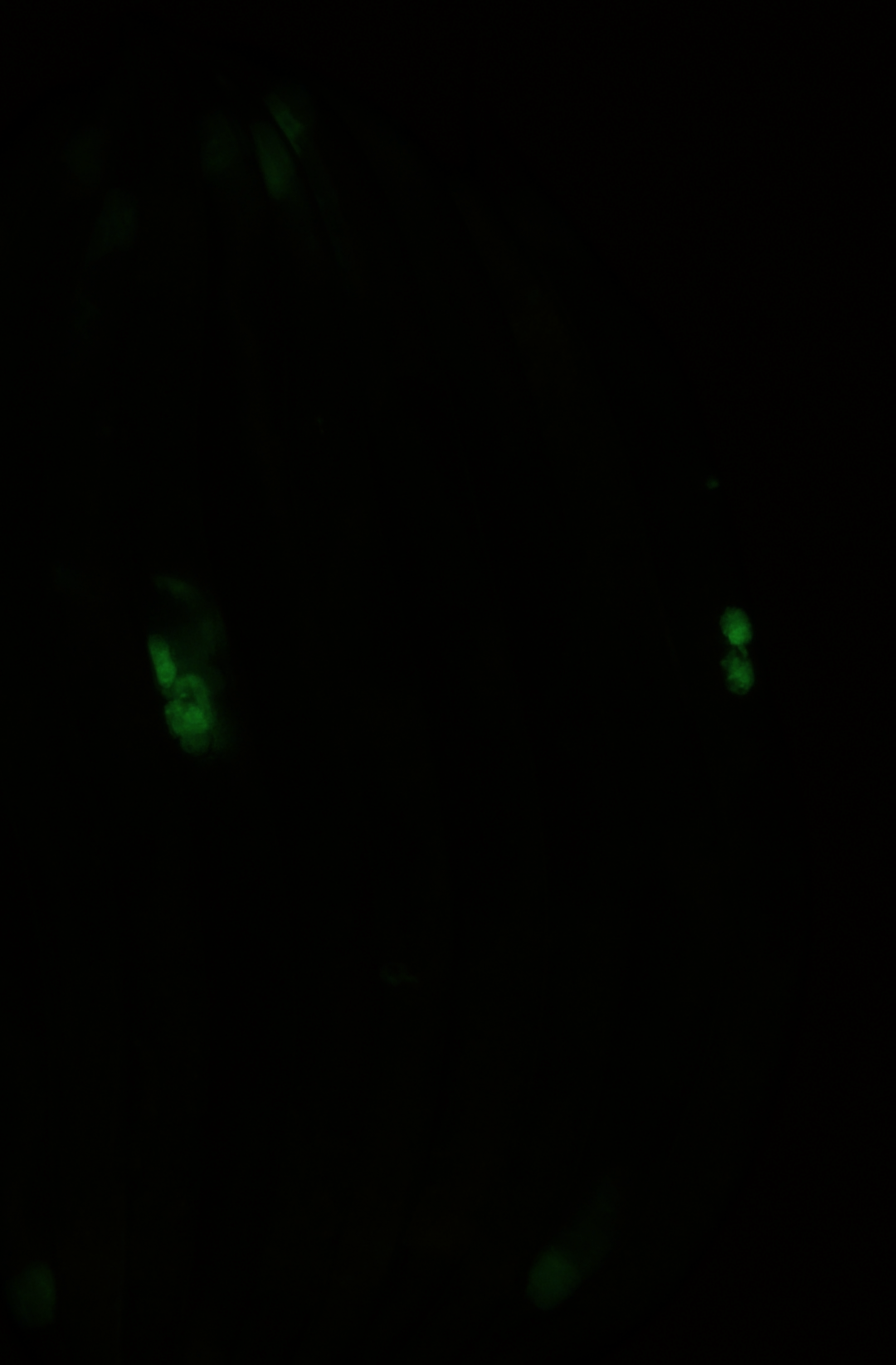

Supplement: Supplementary file 12 — Source data Fig. 6 [file 44318_2025_634_MOESM12_ESM.zip › Figure 6/Source data_Figure 6B/Fecl3 ΔpdeI.tif]

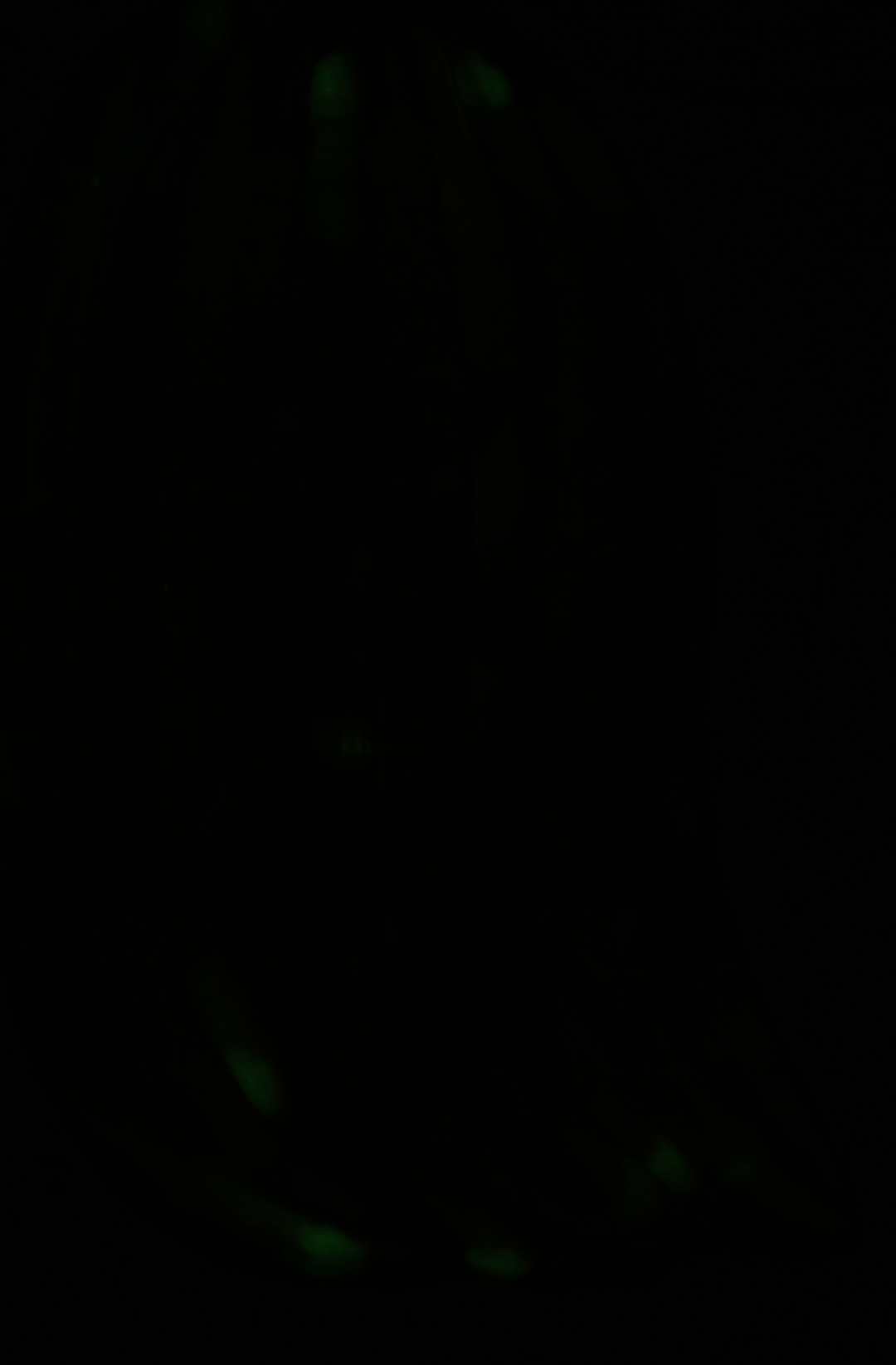

Supplement: Supplementary file 12 — Source data Fig. 6 [file 44318_2025_634_MOESM12_ESM.zip › Figure 6/Source data_Figure 6B/Fecl3 ΔtktA.tif]

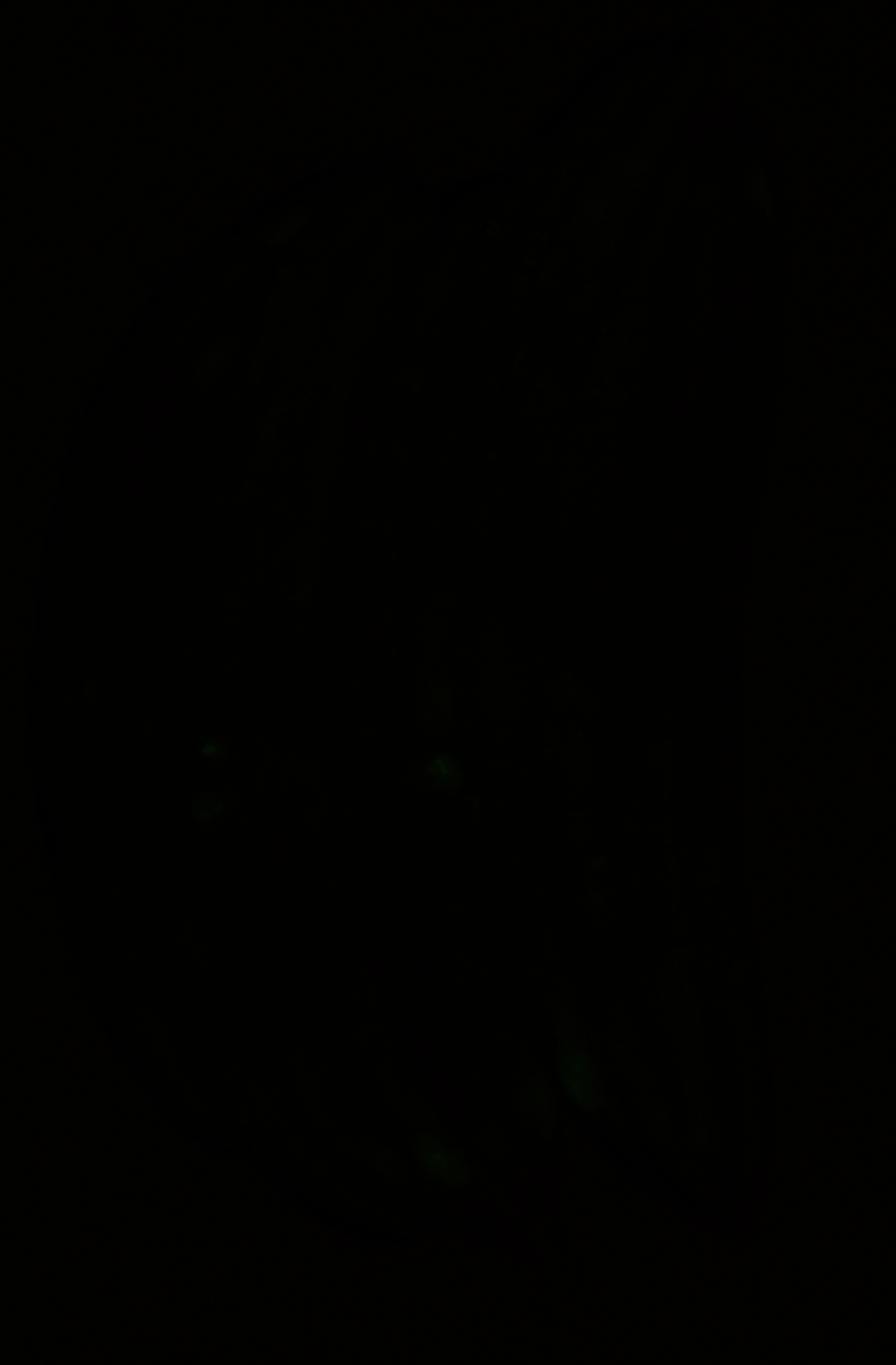

Supplement: Supplementary file 12 — Source data Fig. 6 [file 44318_2025_634_MOESM12_ESM.zip › Figure 6/Source data_Figure 6B/Fecl3 ΔyciA.tif]

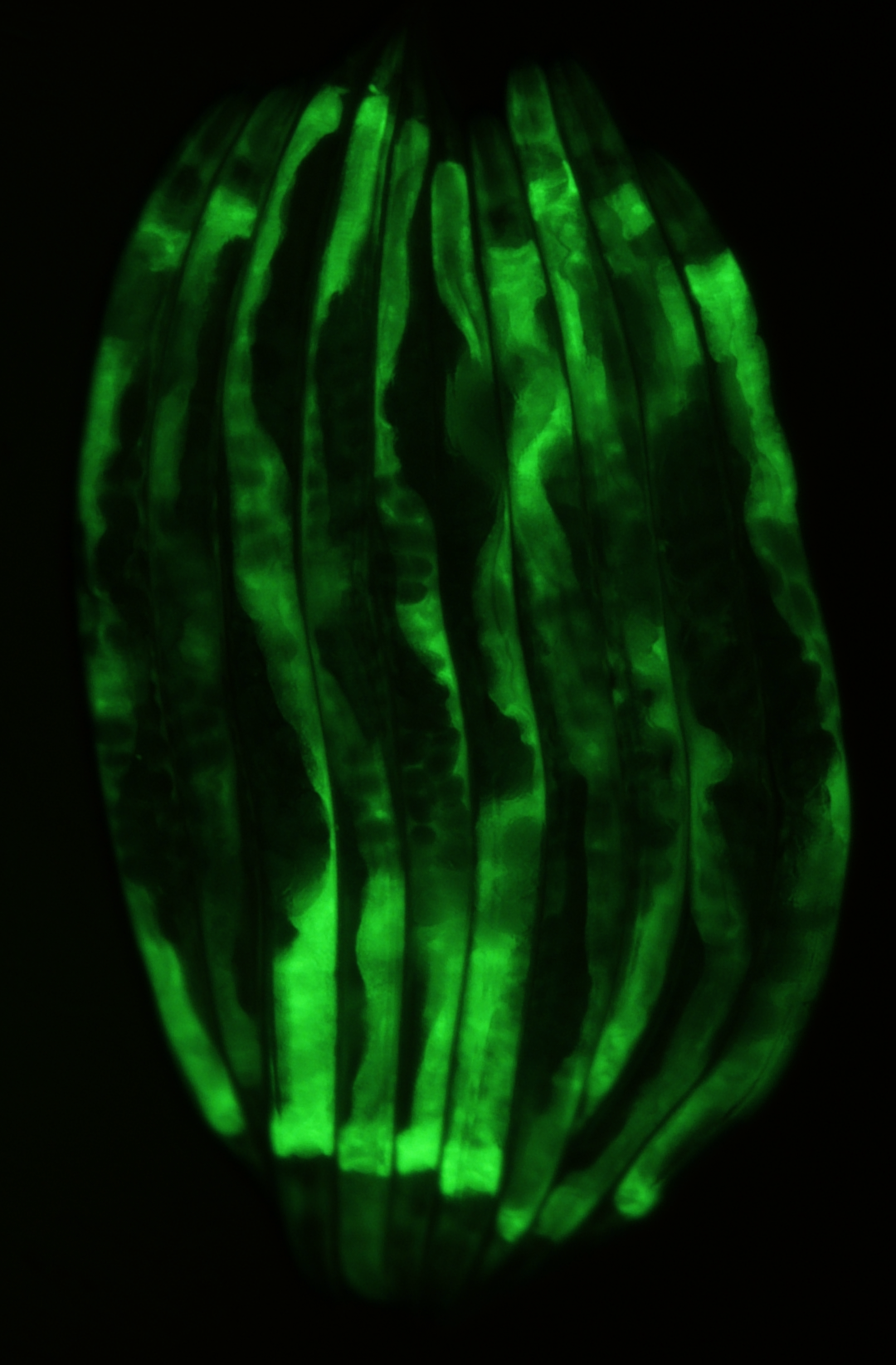

Supplement: Supplementary file 12 — Source data Fig. 6 [file 44318_2025_634_MOESM12_ESM.zip › Figure 6/Source data_Figure 6B/ΔallD.tif]

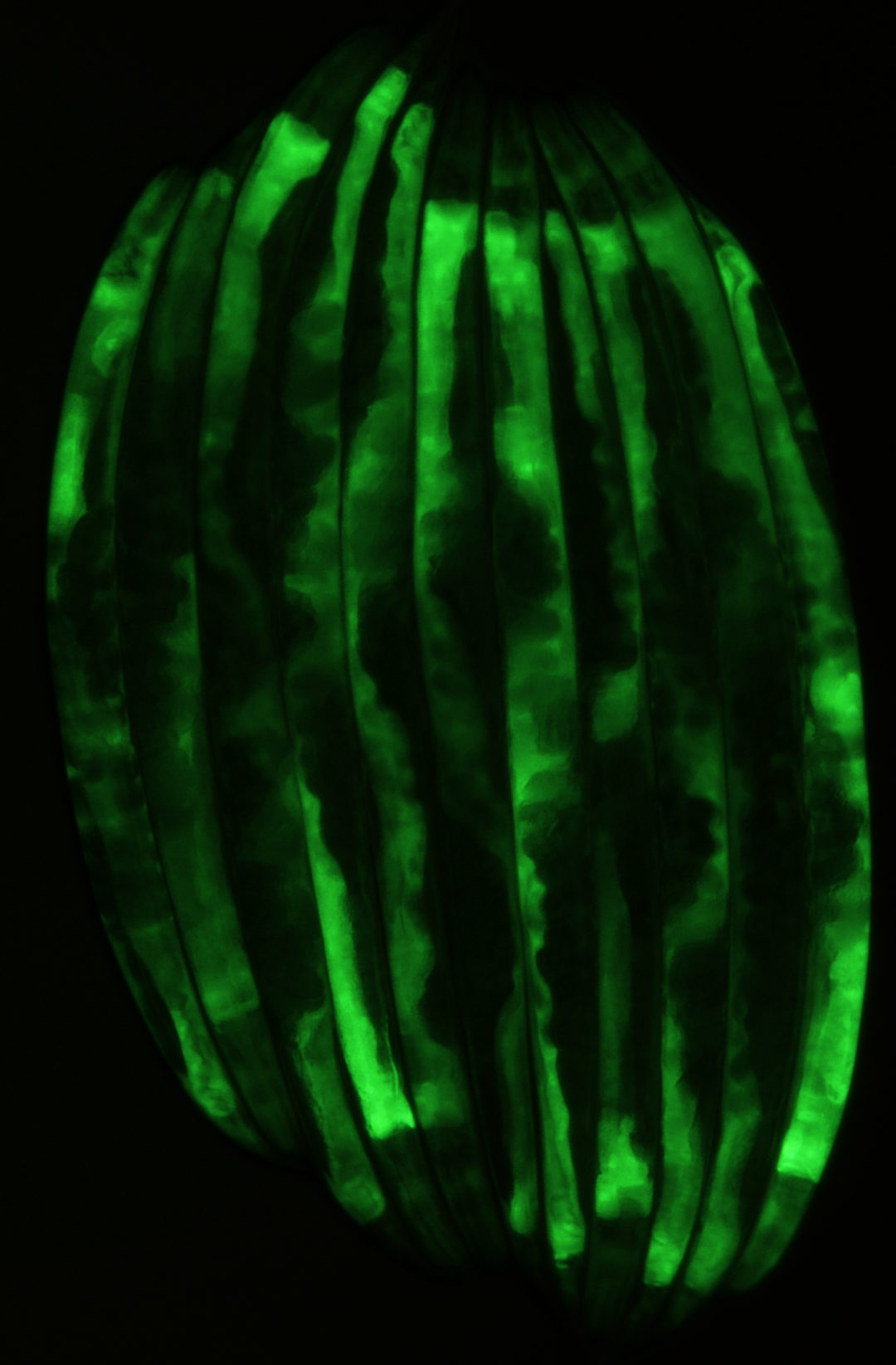

Supplement: Supplementary file 12 — Source data Fig. 6 [file 44318_2025_634_MOESM12_ESM.zip › Figure 6/Source data_Figure 6B/ΔpdeI.tif]

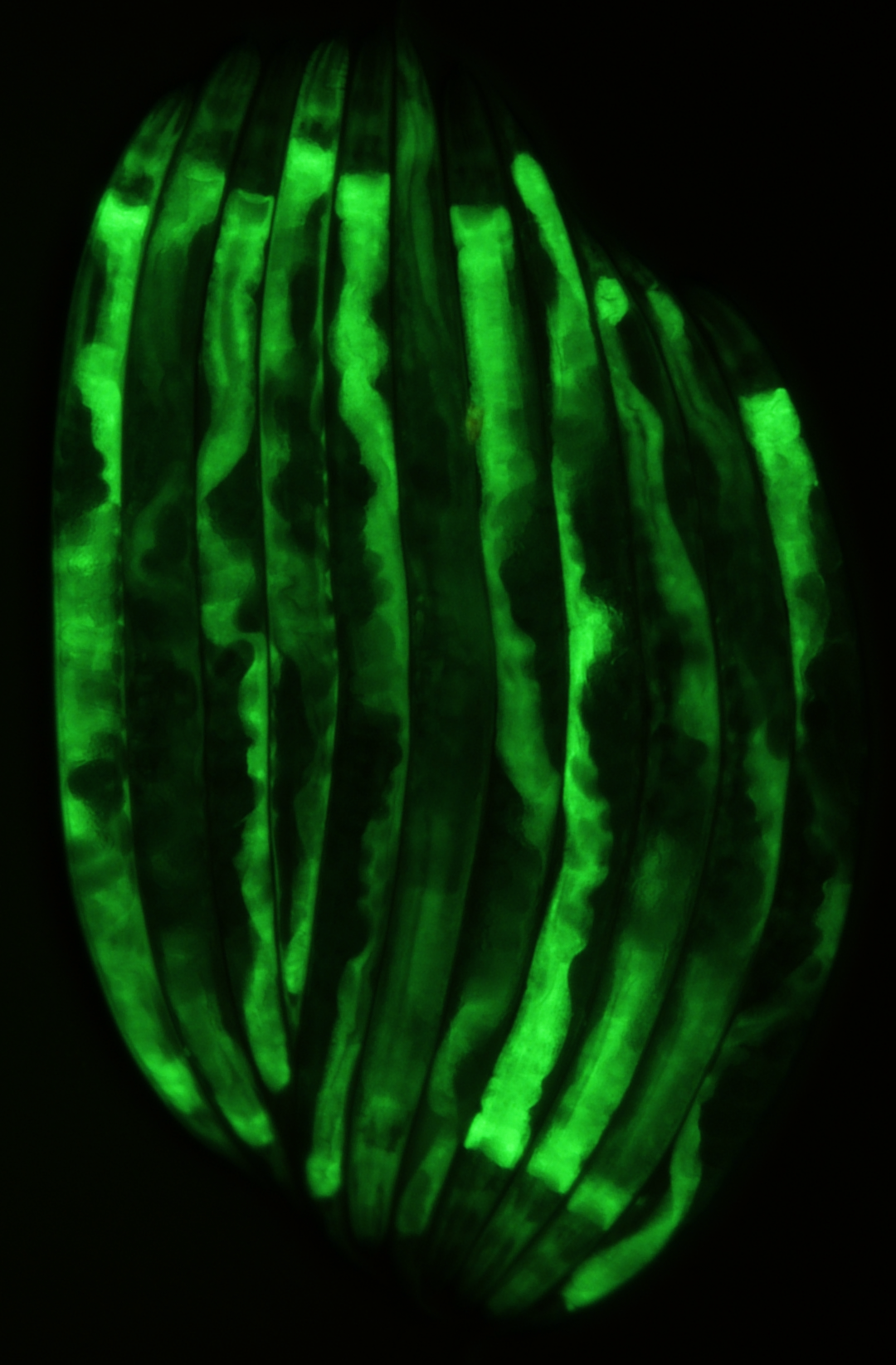

Supplement: Supplementary file 12 — Source data Fig. 6 [file 44318_2025_634_MOESM12_ESM.zip › Figure 6/Source data_Figure 6B/ΔtktA.tif]

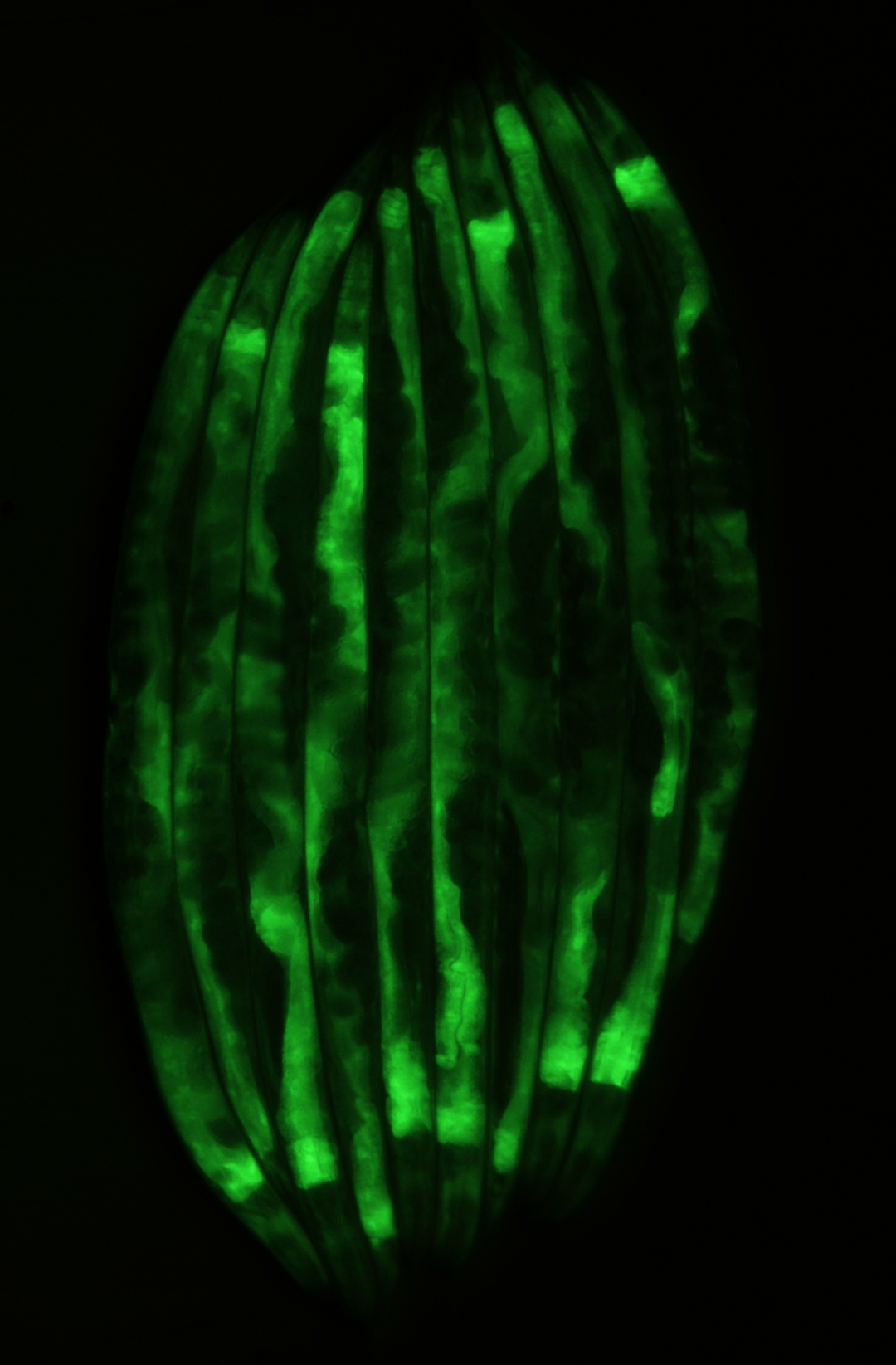

Supplement: Supplementary file 12 — Source data Fig. 6 [file 44318_2025_634_MOESM12_ESM.zip › Figure 6/Source data_Figure 6B/ΔyciA.tif]

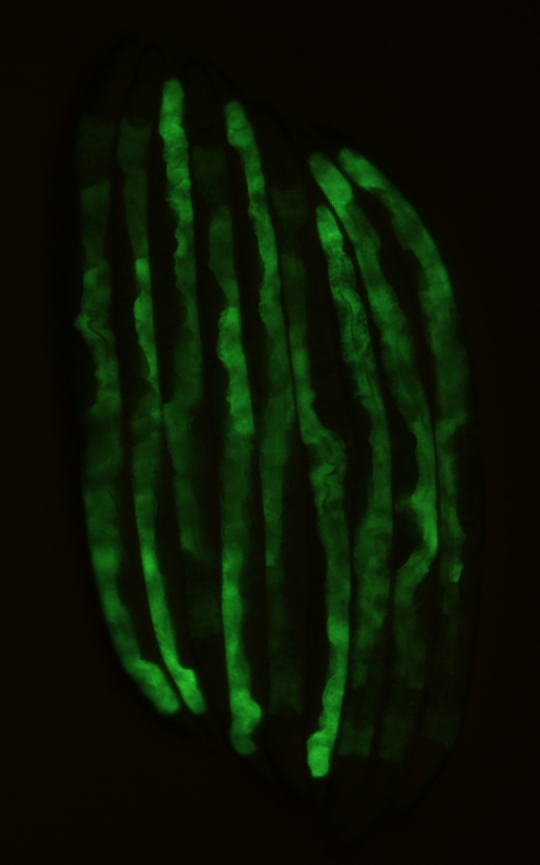

Supplement: Supplementary file 12 — Source data Fig. 6 [file 44318_2025_634_MOESM12_ESM.zip › Figure 6/Source data_Figure 6D/BW25113.tif]

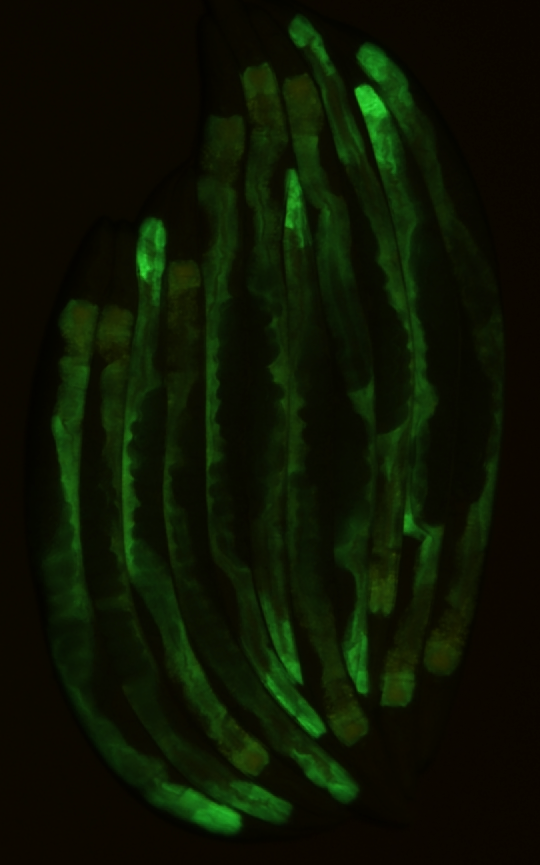

Supplement: Supplementary file 12 — Source data Fig. 6 [file 44318_2025_634_MOESM12_ESM.zip › Figure 6/Source data_Figure 6D/Fecl3 BW25113.tif]

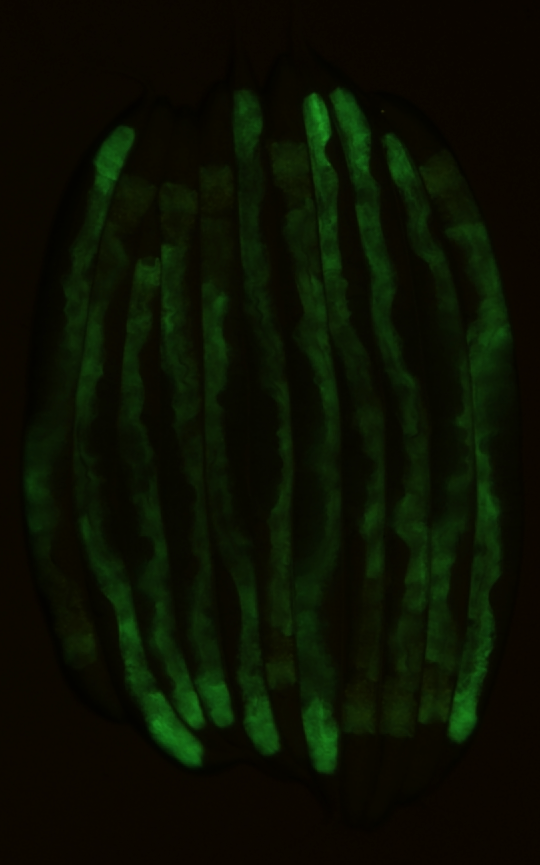

Supplement: Supplementary file 12 — Source data Fig. 6 [file 44318_2025_634_MOESM12_ESM.zip › Figure 6/Source data_Figure 6D/Fecl3 ΔallD.tif]

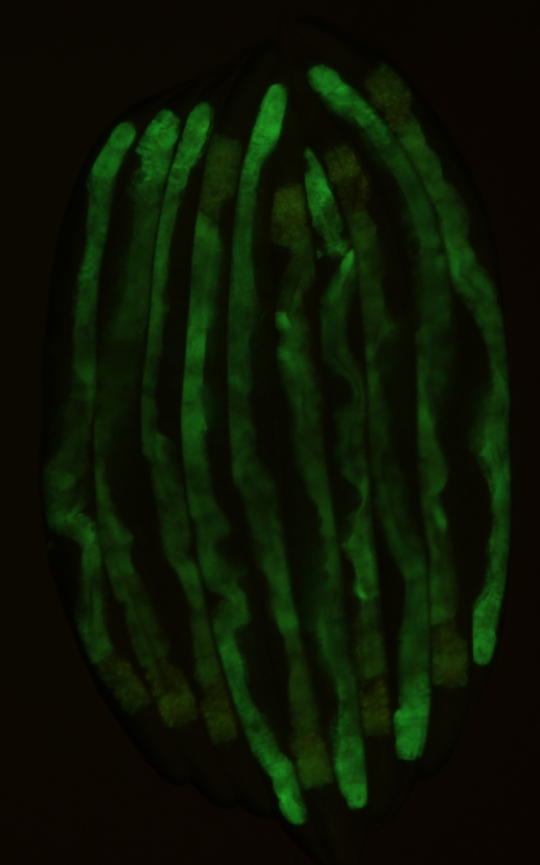

Supplement: Supplementary file 12 — Source data Fig. 6 [file 44318_2025_634_MOESM12_ESM.zip › Figure 6/Source data_Figure 6D/Fecl3 ΔpdeI.tif]

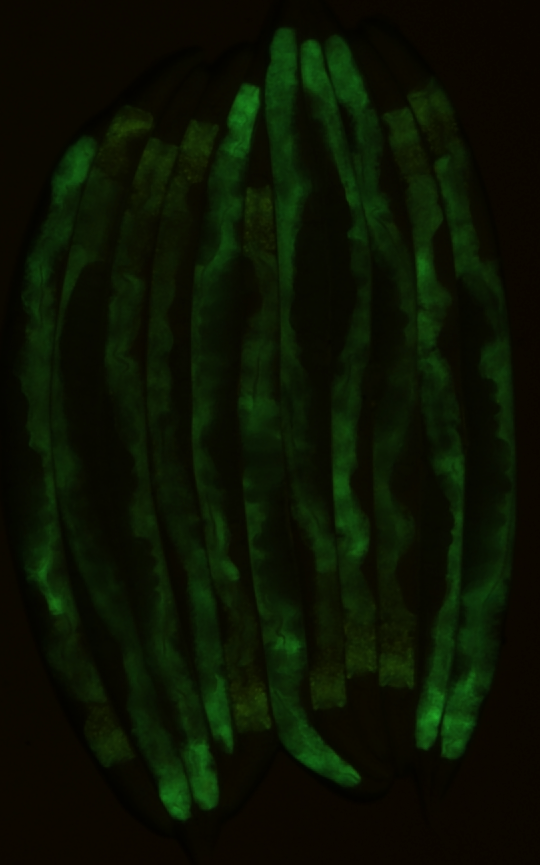

Supplement: Supplementary file 12 — Source data Fig. 6 [file 44318_2025_634_MOESM12_ESM.zip › Figure 6/Source data_Figure 6D/Fecl3 ΔtktA.tif]

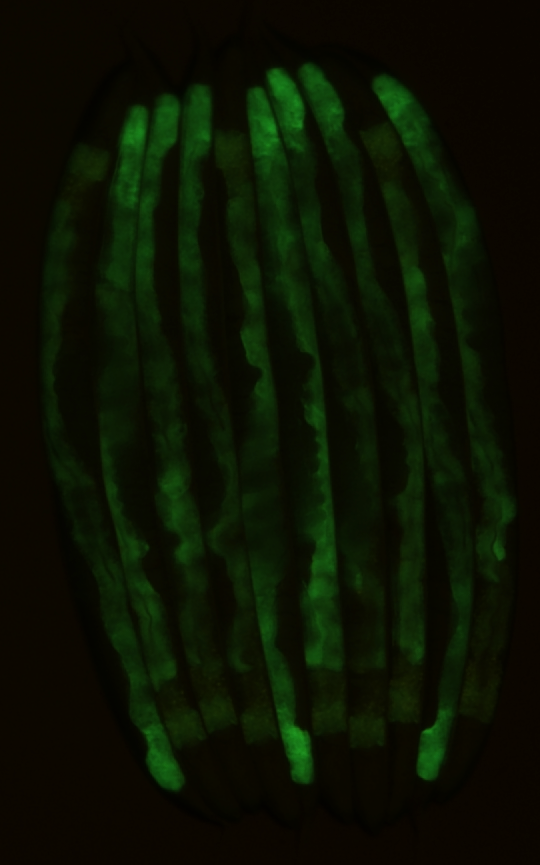

Supplement: Supplementary file 12 — Source data Fig. 6 [file 44318_2025_634_MOESM12_ESM.zip › Figure 6/Source data_Figure 6D/Fecl3 ΔyciA.tif]

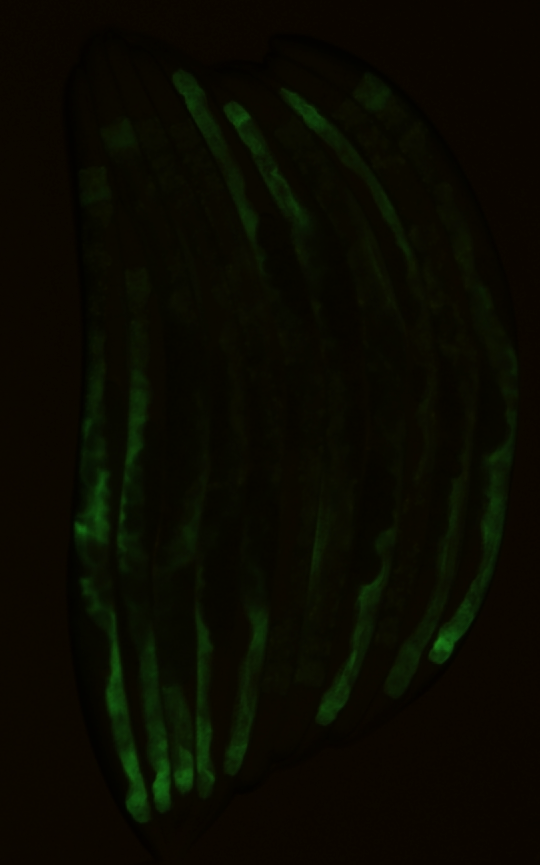

Supplement: Supplementary file 12 — Source data Fig. 6 [file 44318_2025_634_MOESM12_ESM.zip › Figure 6/Source data_Figure 6D/ΔallD.tif]

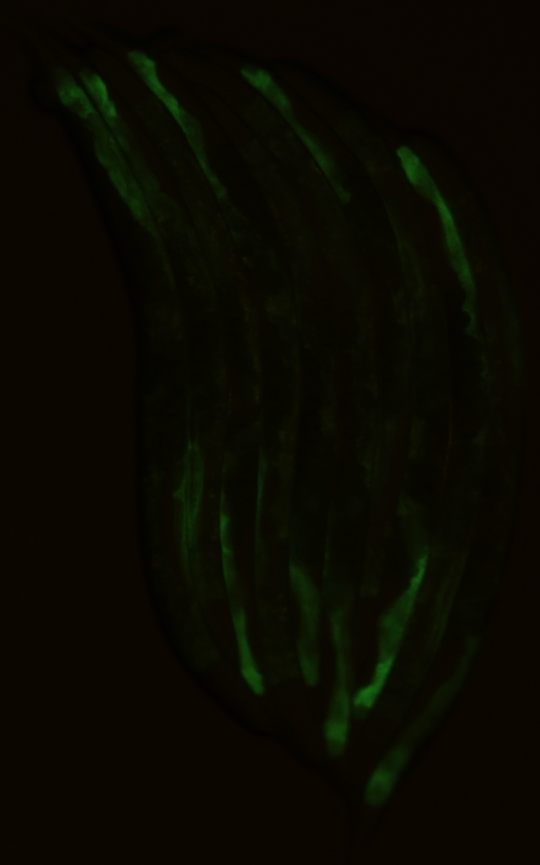

Supplement: Supplementary file 12 — Source data Fig. 6 [file 44318_2025_634_MOESM12_ESM.zip › Figure 6/Source data_Figure 6D/ΔpdeI.tif]

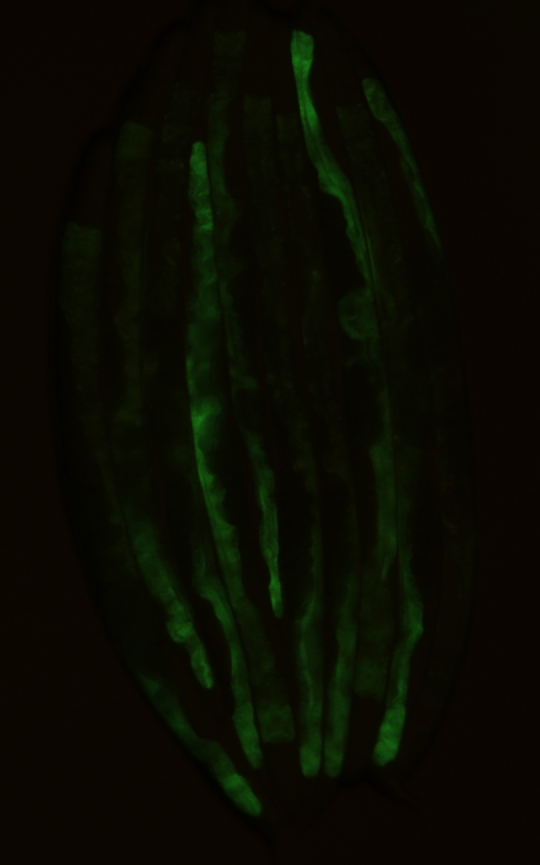

Supplement: Supplementary file 12 — Source data Fig. 6 [file 44318_2025_634_MOESM12_ESM.zip › Figure 6/Source data_Figure 6D/ΔtktA.tif]

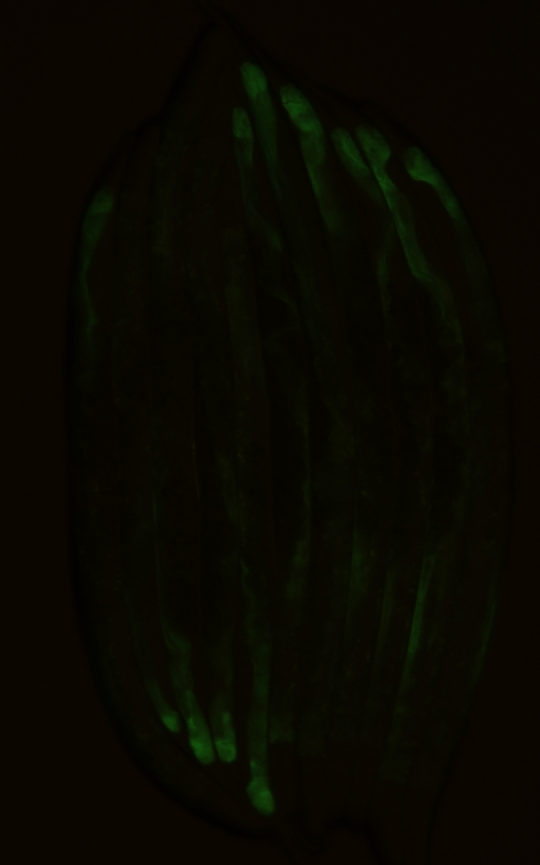

Supplement: Supplementary file 12 — Source data Fig. 6 [file 44318_2025_634_MOESM12_ESM.zip › Figure 6/Source data_Figure 6D/ΔyciA.tif]

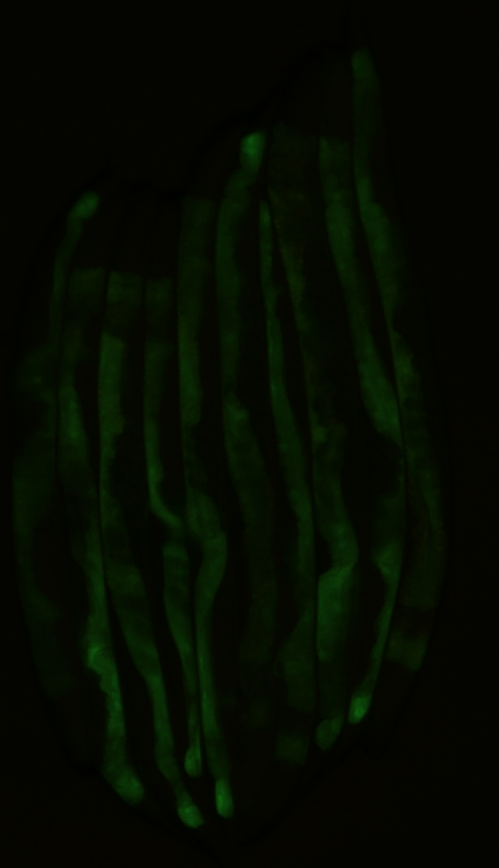

Supplement: Supplementary file 13 — Source data Fig. 7 [file 44318_2025_634_MOESM13_ESM.zip › Figure 7/Source data_Figure 7B/BP.tif]

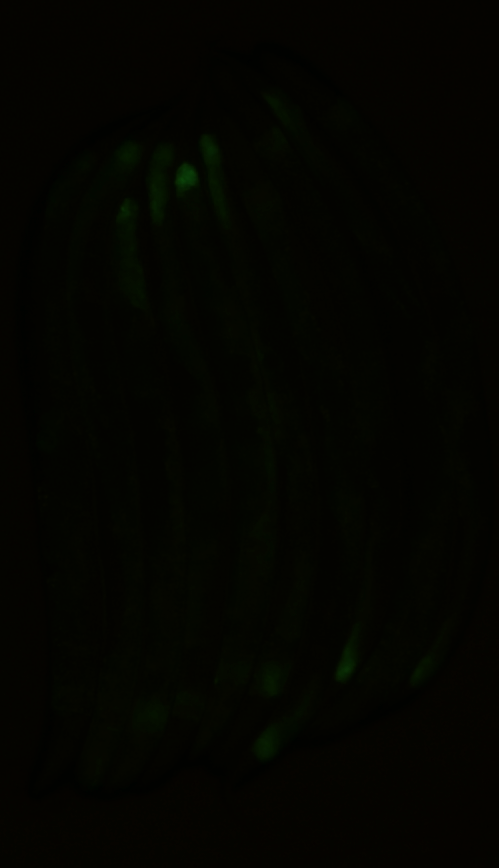

Supplement: Supplementary file 13 — Source data Fig. 7 [file 44318_2025_634_MOESM13_ESM.zip › Figure 7/Source data_Figure 7B/Control.tif]

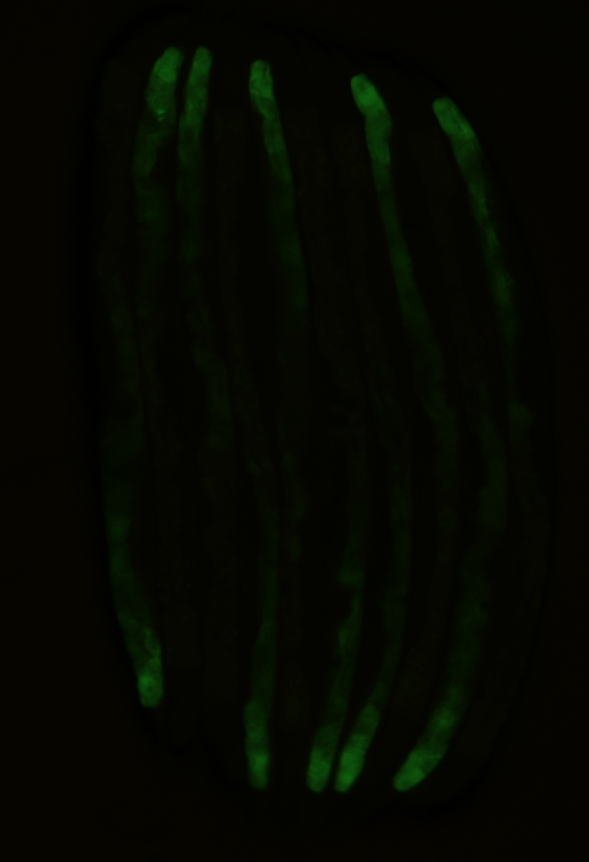

Supplement: Supplementary file 13 — Source data Fig. 7 [file 44318_2025_634_MOESM13_ESM.zip › Figure 7/Source data_Figure 7D/BP.tif]

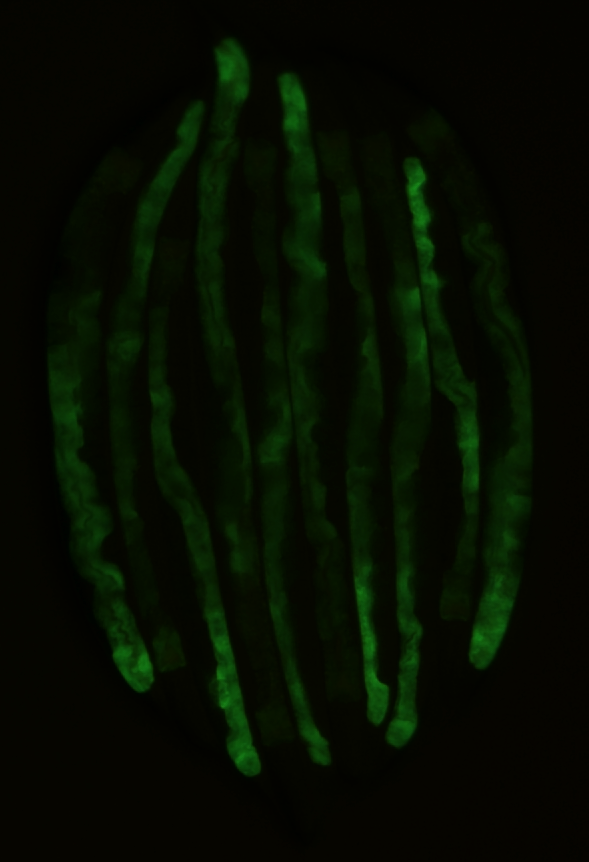

Supplement: Supplementary file 13 — Source data Fig. 7 [file 44318_2025_634_MOESM13_ESM.zip › Figure 7/Source data_Figure 7D/Control.tif]

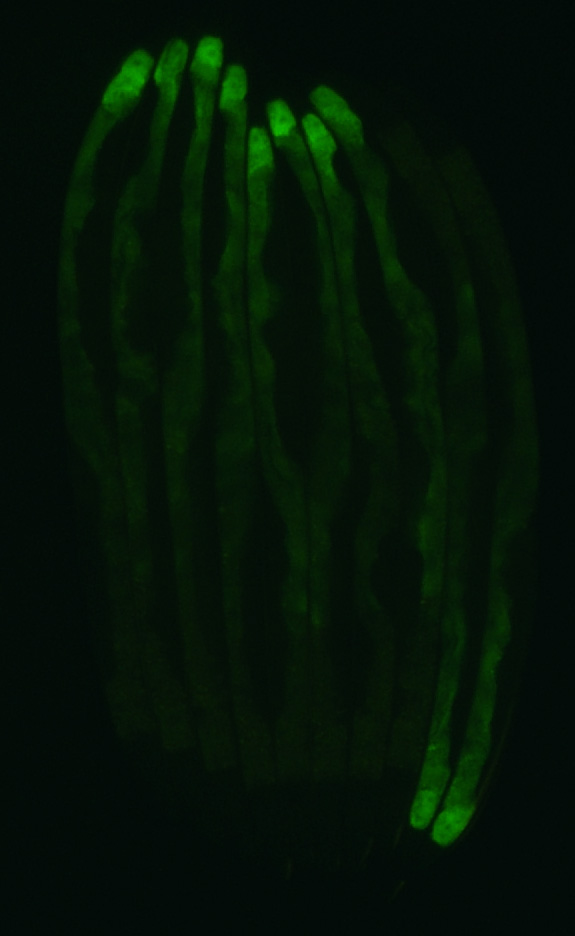

Supplement: Supplementary file 15 — Figure Source Data EV figures [file 44318_2025_634_MOESM15_ESM.zip › EMBOJ-2025-121287-T_SourceDataEVFigures/Figure EV1/Source data_Figure EV1B/BW25113.jpg]

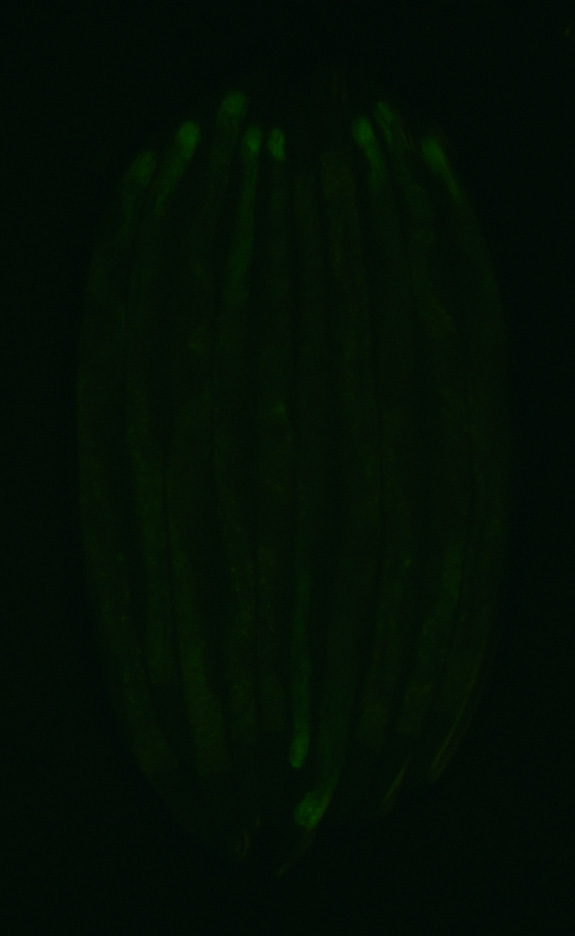

Supplement: Supplementary file 15 — Figure Source Data EV figures [file 44318_2025_634_MOESM15_ESM.zip › EMBOJ-2025-121287-T_SourceDataEVFigures/Figure EV1/Source data_Figure EV1B/ΔallD.jpg]

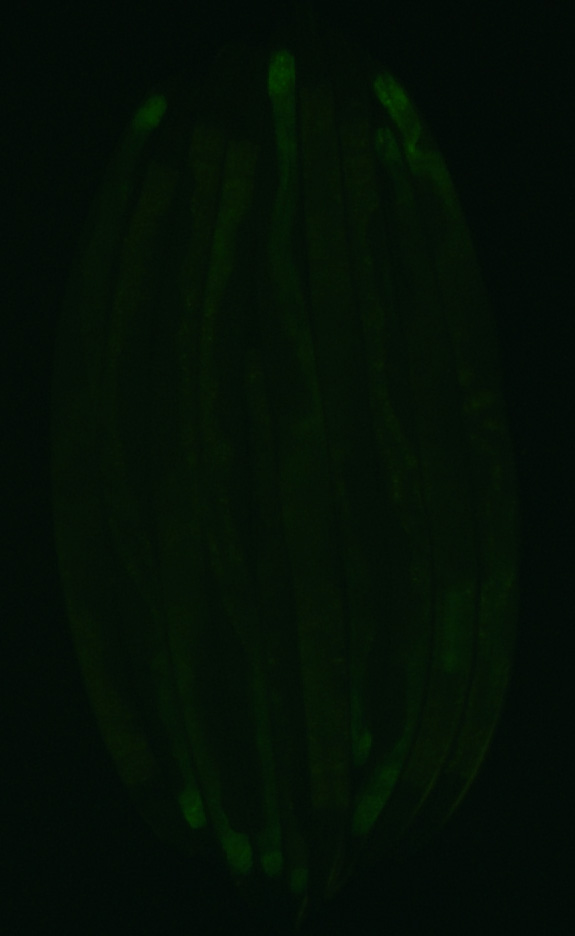

Supplement: Supplementary file 15 — Figure Source Data EV figures [file 44318_2025_634_MOESM15_ESM.zip › EMBOJ-2025-121287-T_SourceDataEVFigures/Figure EV1/Source data_Figure EV1B/ΔcpxR.jpg]

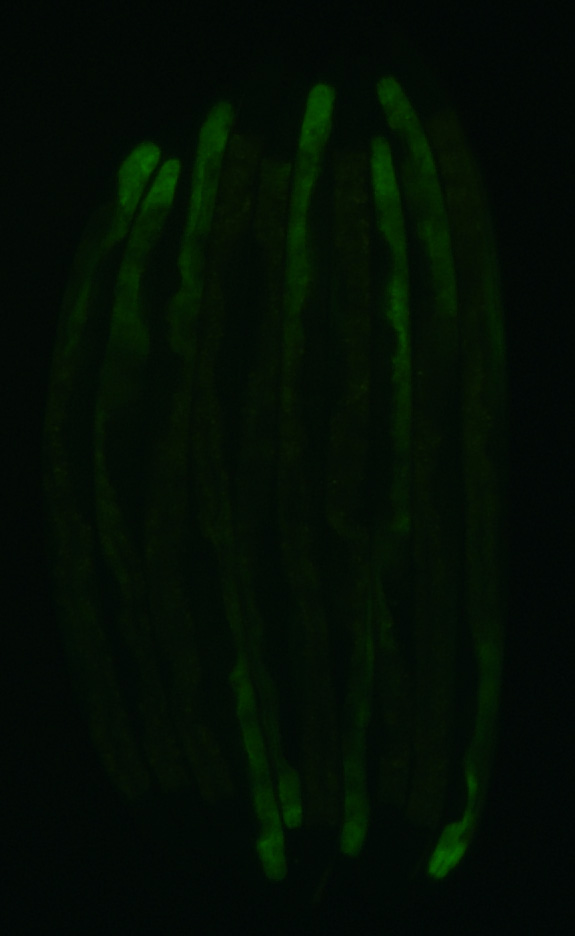

Supplement: Supplementary file 15 — Figure Source Data EV figures [file 44318_2025_634_MOESM15_ESM.zip › EMBOJ-2025-121287-T_SourceDataEVFigures/Figure EV1/Source data_Figure EV1B/ΔcutC.jpg]

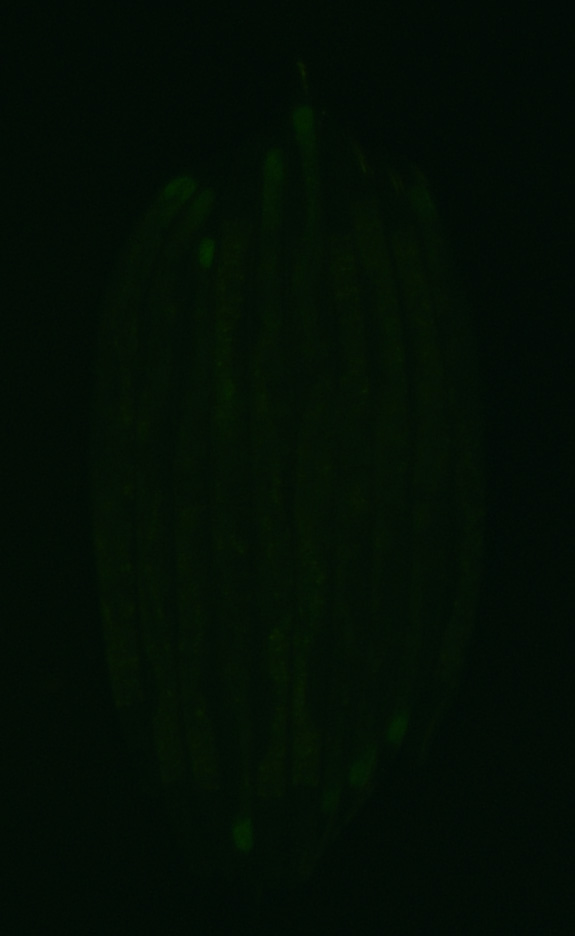

Supplement: Supplementary file 15 — Figure Source Data EV figures [file 44318_2025_634_MOESM15_ESM.zip › EMBOJ-2025-121287-T_SourceDataEVFigures/Figure EV1/Source data_Figure EV1B/ΔcyoA.jpg]

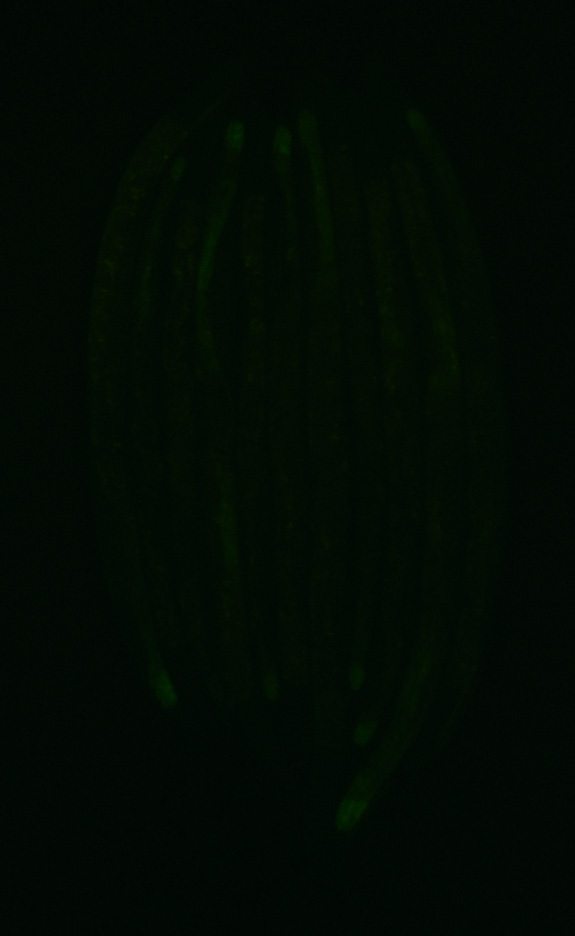

Supplement: Supplementary file 15 — Figure Source Data EV figures [file 44318_2025_634_MOESM15_ESM.zip › EMBOJ-2025-121287-T_SourceDataEVFigures/Figure EV1/Source data_Figure EV1B/ΔcyoB .jpg]

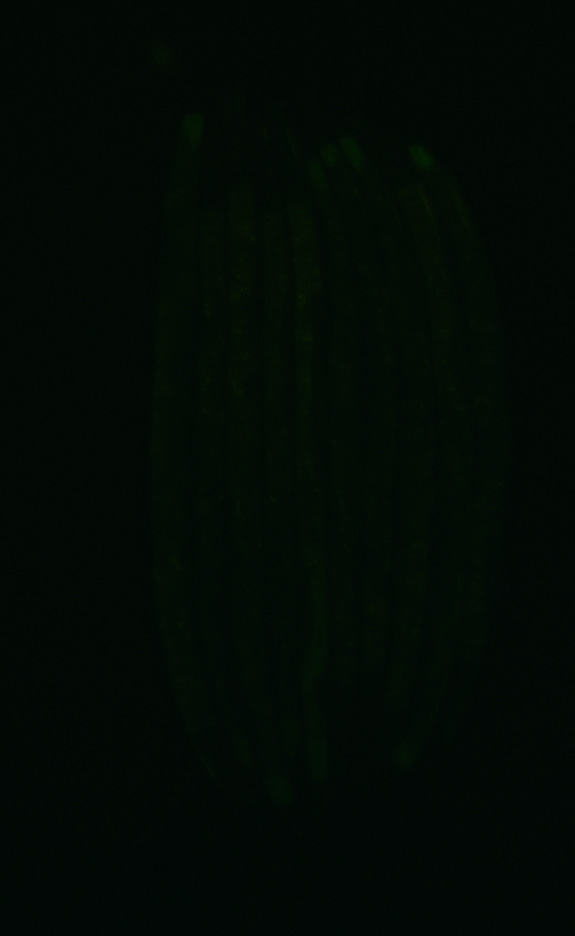

Supplement: Supplementary file 15 — Figure Source Data EV figures [file 44318_2025_634_MOESM15_ESM.zip › EMBOJ-2025-121287-T_SourceDataEVFigures/Figure EV1/Source data_Figure EV1B/ΔcyoC.jpg]

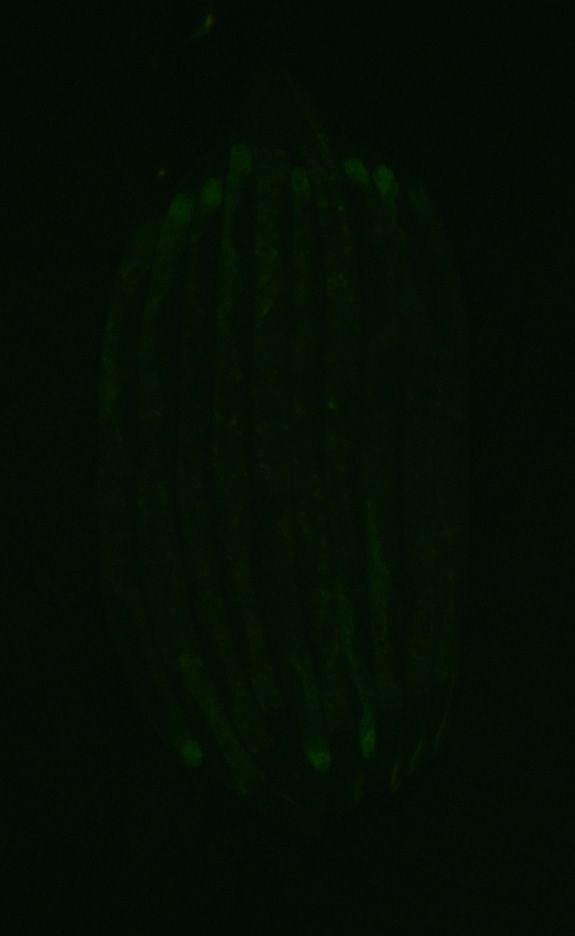

Supplement: Supplementary file 15 — Figure Source Data EV figures [file 44318_2025_634_MOESM15_ESM.zip › EMBOJ-2025-121287-T_SourceDataEVFigures/Figure EV1/Source data_Figure EV1B/ΔcyoD.jpg]

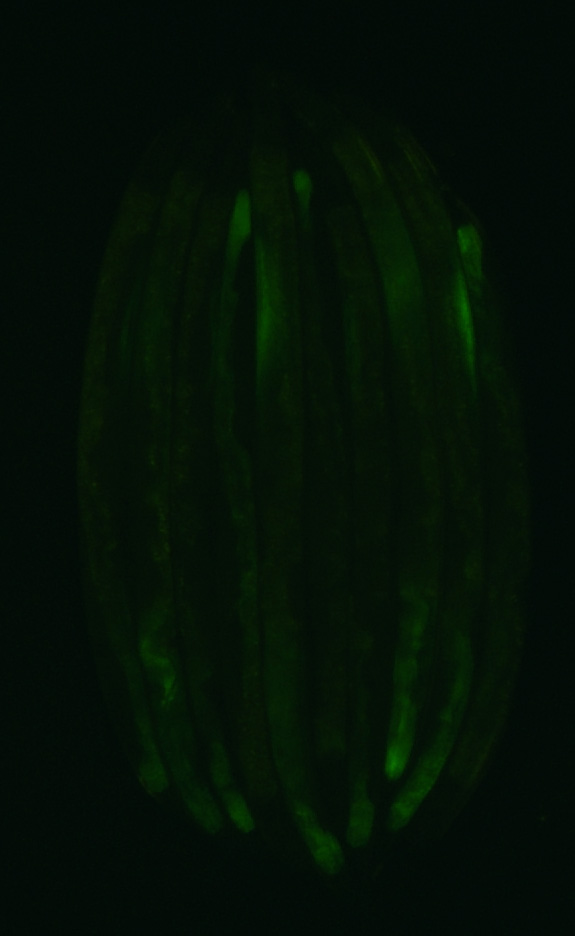

Supplement: Supplementary file 15 — Figure Source Data EV figures [file 44318_2025_634_MOESM15_ESM.zip › EMBOJ-2025-121287-T_SourceDataEVFigures/Figure EV1/Source data_Figure EV1B/ΔdmsA.jpg]

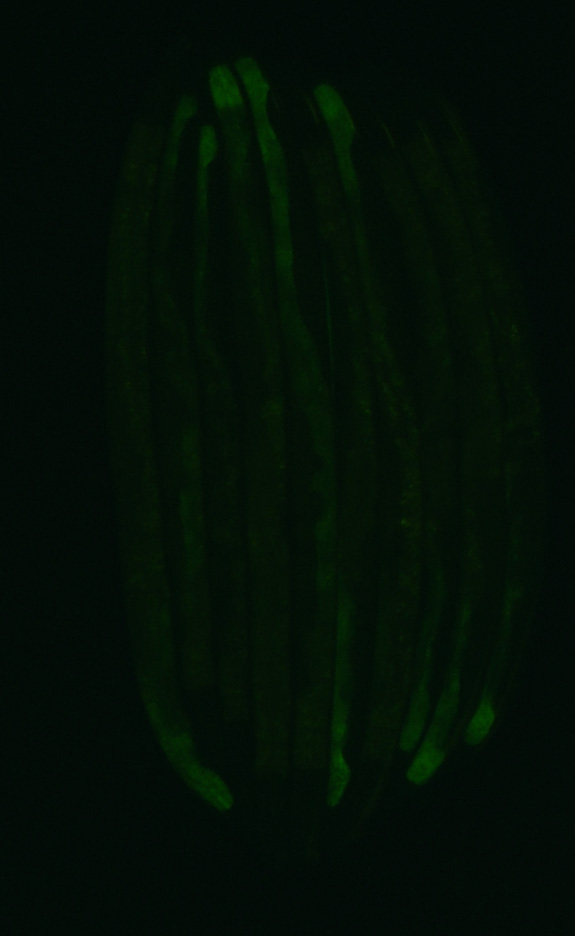

Supplement: Supplementary file 15 — Figure Source Data EV figures [file 44318_2025_634_MOESM15_ESM.zip › EMBOJ-2025-121287-T_SourceDataEVFigures/Figure EV1/Source data_Figure EV1B/ΔfdrA .jpg]

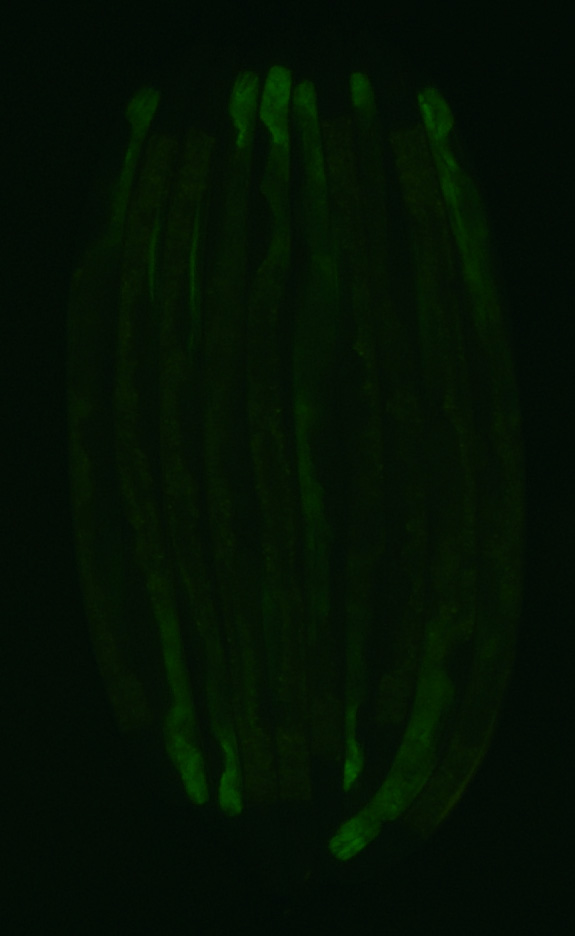

Supplement: Supplementary file 15 — Figure Source Data EV figures [file 44318_2025_634_MOESM15_ESM.zip › EMBOJ-2025-121287-T_SourceDataEVFigures/Figure EV1/Source data_Figure EV1B/ΔnarY.jpg]

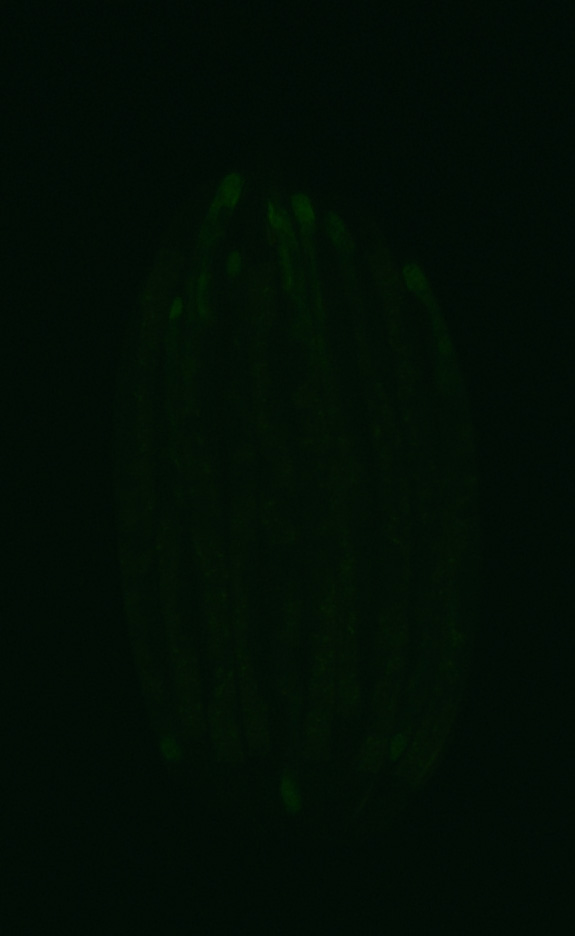

Supplement: Supplementary file 15 — Figure Source Data EV figures [file 44318_2025_634_MOESM15_ESM.zip › EMBOJ-2025-121287-T_SourceDataEVFigures/Figure EV1/Source data_Figure EV1B/ΔpdeI.jpg]

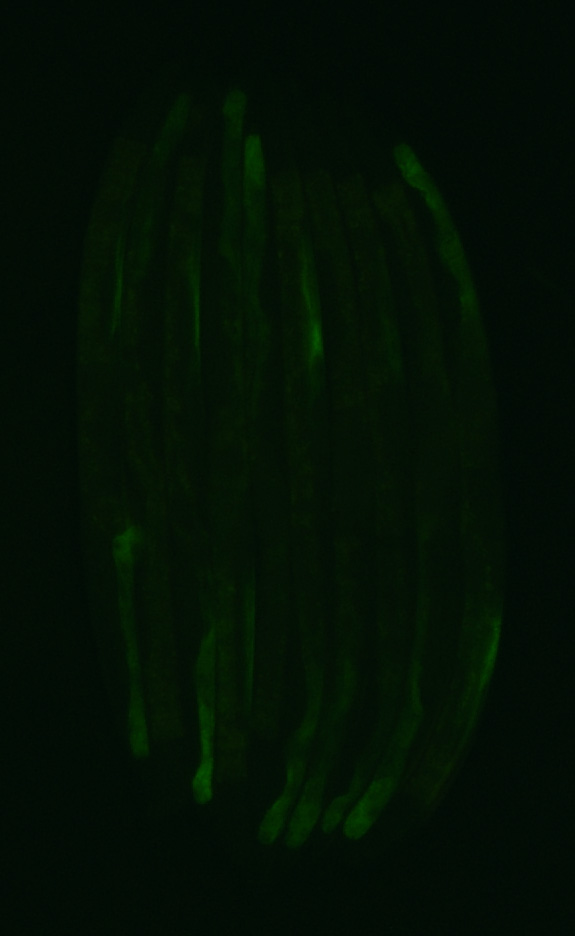

Supplement: Supplementary file 15 — Figure Source Data EV figures [file 44318_2025_634_MOESM15_ESM.zip › EMBOJ-2025-121287-T_SourceDataEVFigures/Figure EV1/Source data_Figure EV1B/ΔpliG .jpg]

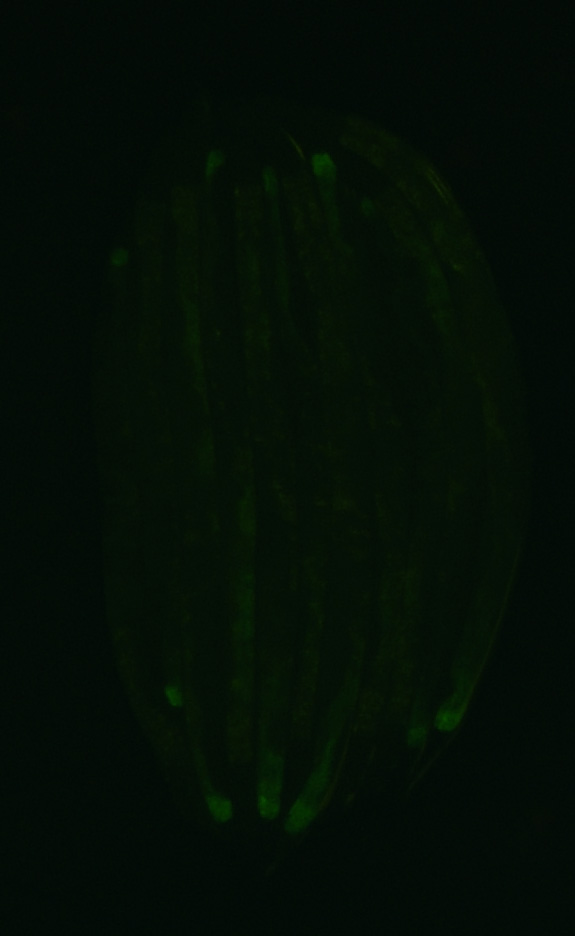

Supplement: Supplementary file 15 — Figure Source Data EV figures [file 44318_2025_634_MOESM15_ESM.zip › EMBOJ-2025-121287-T_SourceDataEVFigures/Figure EV1/Source data_Figure EV1B/ΔrlmL.jpg]

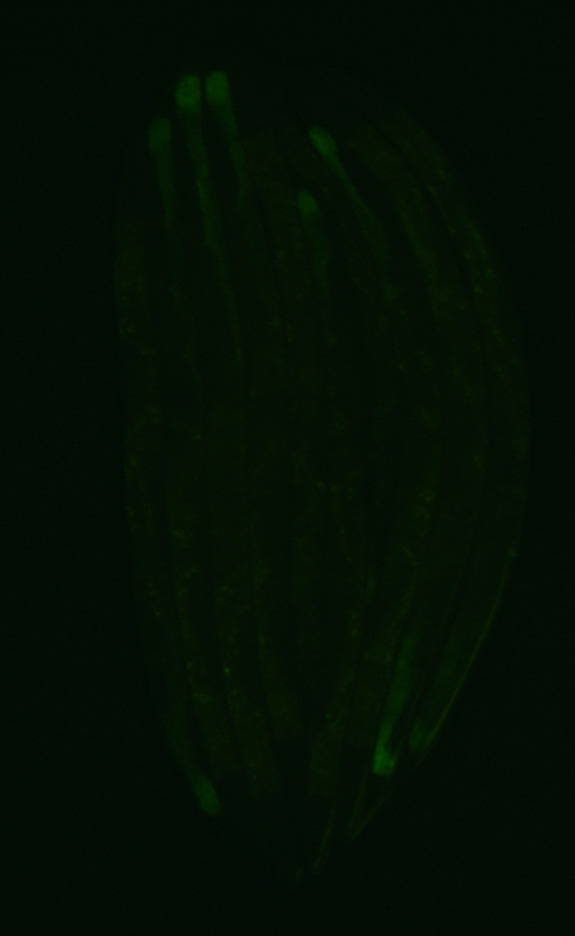

Supplement: Supplementary file 15 — Figure Source Data EV figures [file 44318_2025_634_MOESM15_ESM.zip › EMBOJ-2025-121287-T_SourceDataEVFigures/Figure EV1/Source data_Figure EV1B/ΔroxA.jpg]

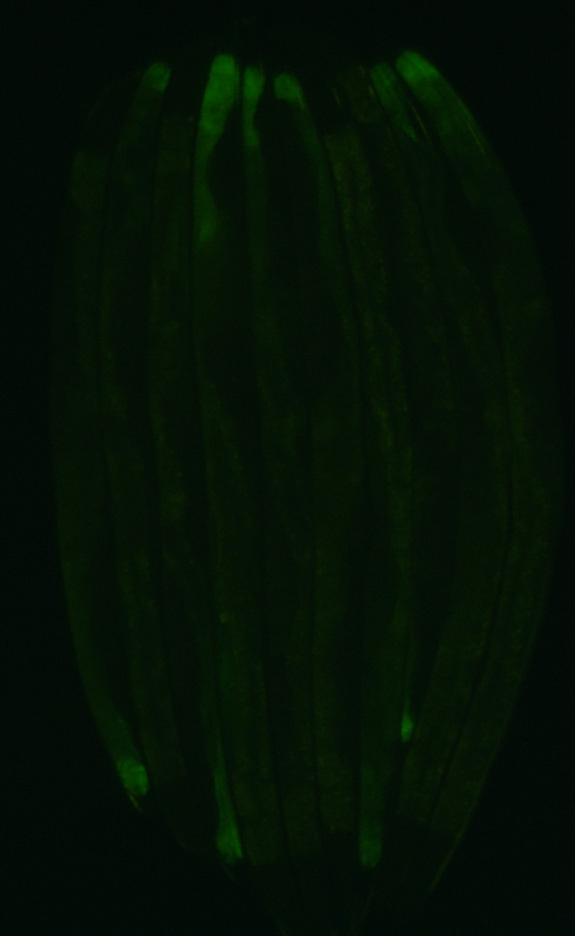

Supplement: Supplementary file 15 — Figure Source Data EV figures [file 44318_2025_634_MOESM15_ESM.zip › EMBOJ-2025-121287-T_SourceDataEVFigures/Figure EV1/Source data_Figure EV1B/ΔsdhB.jpg]

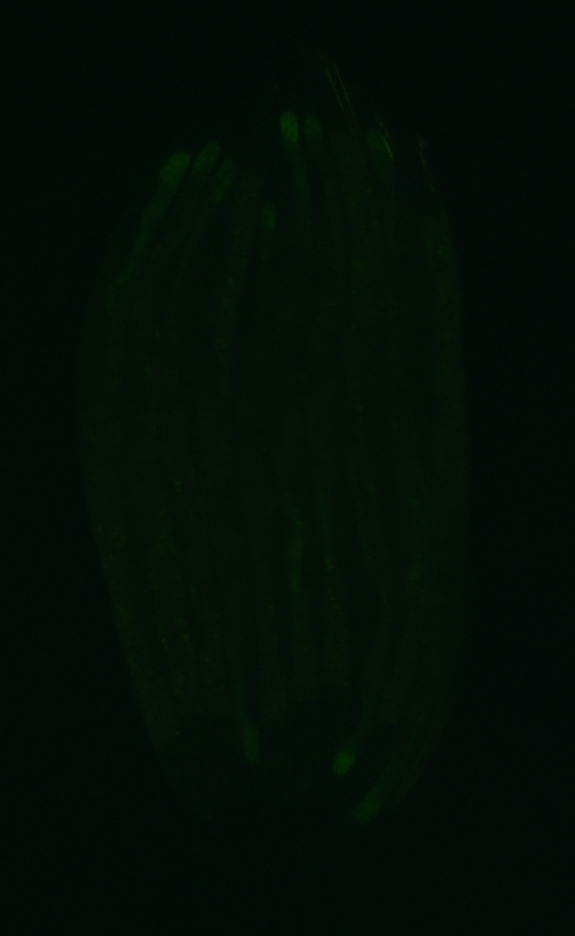

Supplement: Supplementary file 15 — Figure Source Data EV figures [file 44318_2025_634_MOESM15_ESM.zip › EMBOJ-2025-121287-T_SourceDataEVFigures/Figure EV1/Source data_Figure EV1B/ΔstfP.jpg]

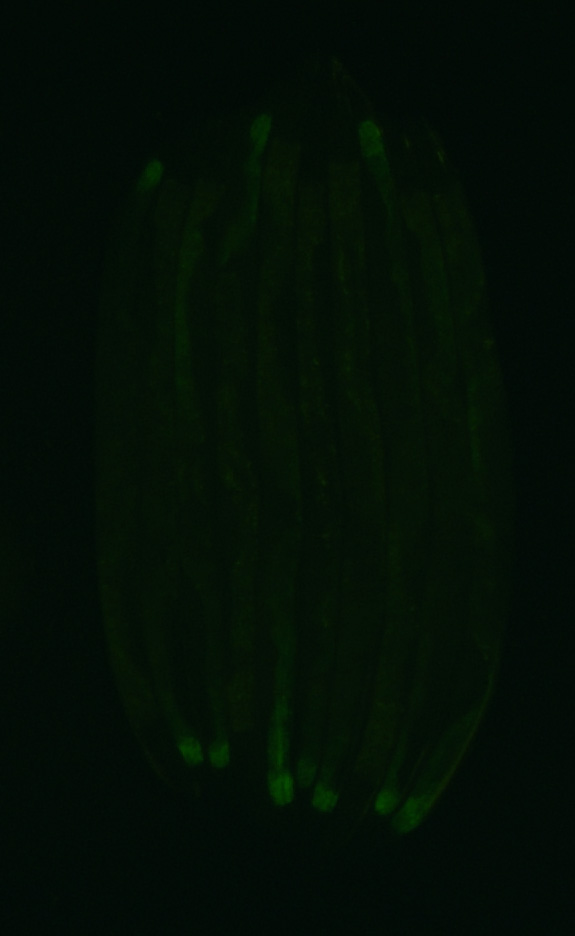

Supplement: Supplementary file 15 — Figure Source Data EV figures [file 44318_2025_634_MOESM15_ESM.zip › EMBOJ-2025-121287-T_SourceDataEVFigures/Figure EV1/Source data_Figure EV1B/ΔtktA.jpg]

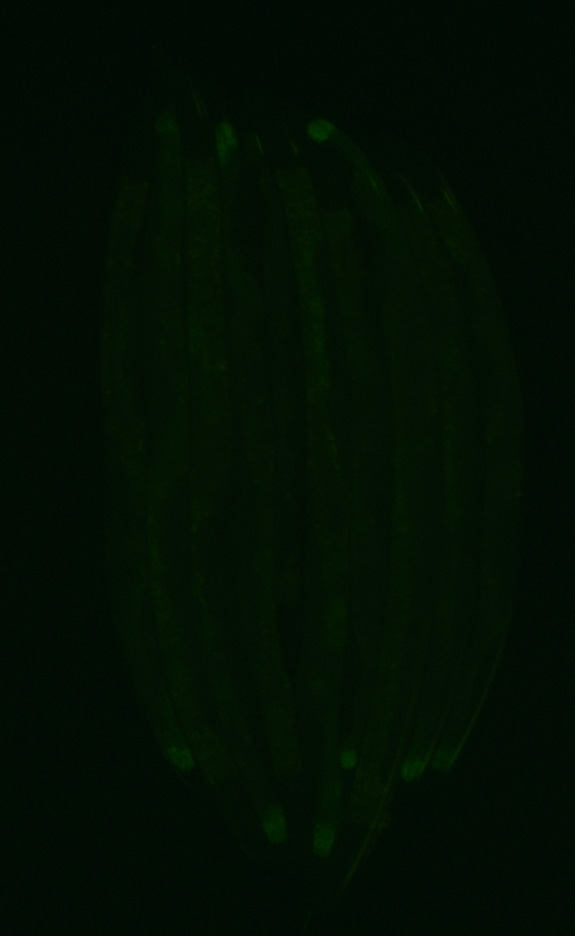

Supplement: Supplementary file 15 — Figure Source Data EV figures [file 44318_2025_634_MOESM15_ESM.zip › EMBOJ-2025-121287-T_SourceDataEVFigures/Figure EV1/Source data_Figure EV1B/ΔtqsA.jpg]

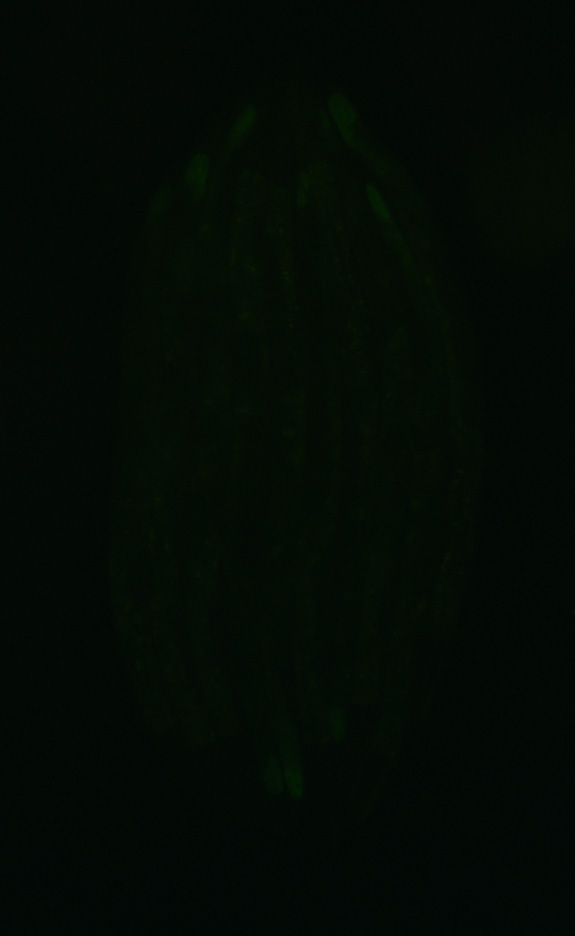

Supplement: Supplementary file 15 — Figure Source Data EV figures [file 44318_2025_634_MOESM15_ESM.zip › EMBOJ-2025-121287-T_SourceDataEVFigures/Figure EV1/Source data_Figure EV1B/ΔybaN.jpg]

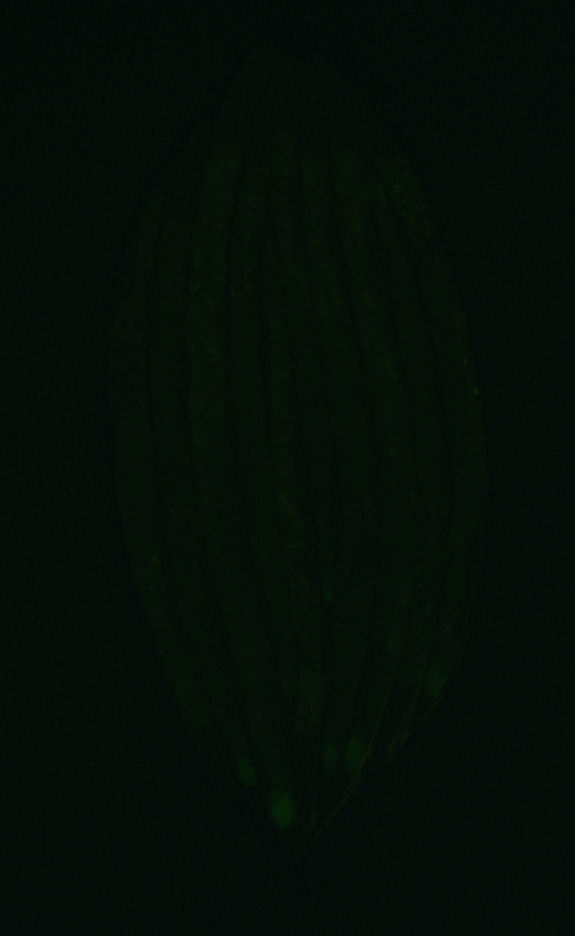

Supplement: Supplementary file 15 — Figure Source Data EV figures [file 44318_2025_634_MOESM15_ESM.zip › EMBOJ-2025-121287-T_SourceDataEVFigures/Figure EV1/Source data_Figure EV1B/ΔybfB.jpg]

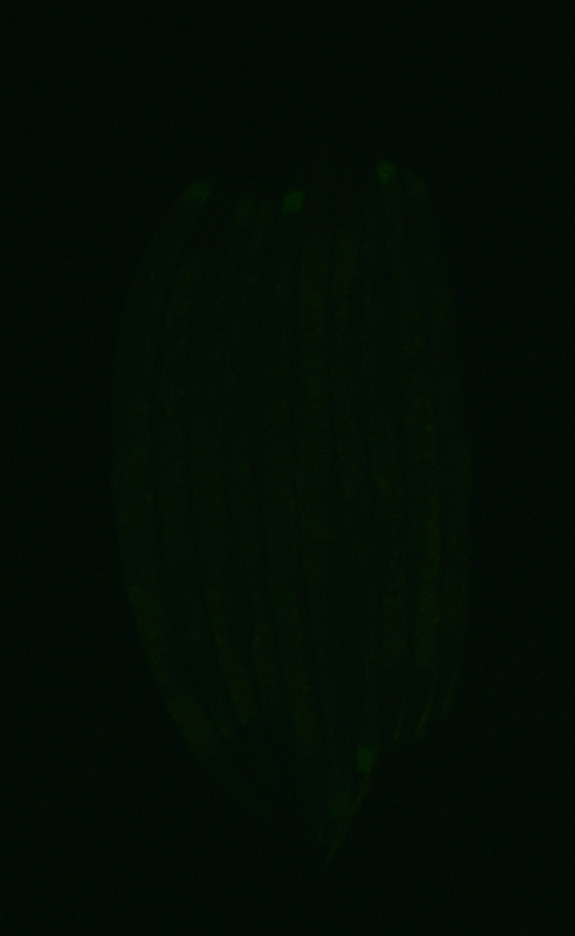

Supplement: Supplementary file 15 — Figure Source Data EV figures [file 44318_2025_634_MOESM15_ESM.zip › EMBOJ-2025-121287-T_SourceDataEVFigures/Figure EV1/Source data_Figure EV1B/ΔycbK.jpg]

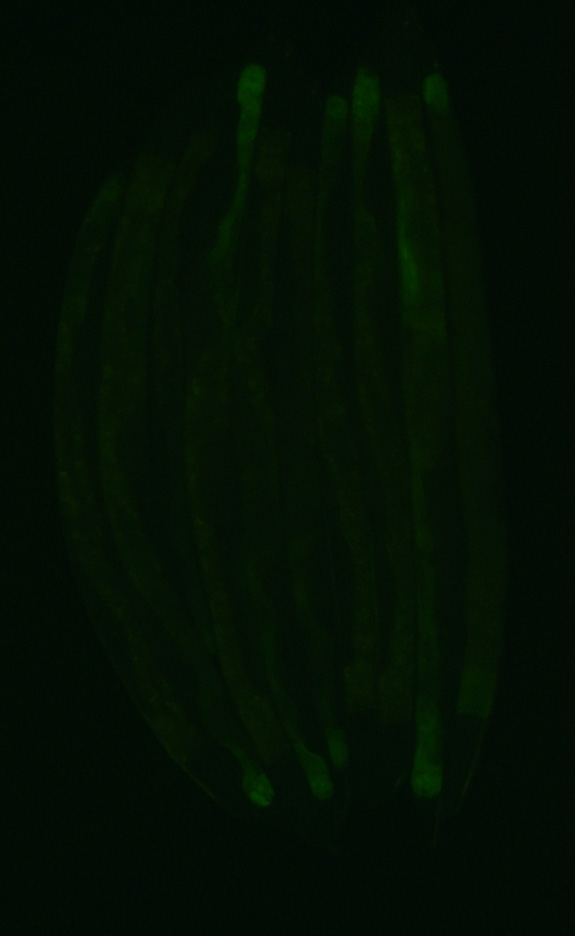

Supplement: Supplementary file 15 — Figure Source Data EV figures [file 44318_2025_634_MOESM15_ESM.zip › EMBOJ-2025-121287-T_SourceDataEVFigures/Figure EV1/Source data_Figure EV1B/ΔyccA.jpg]

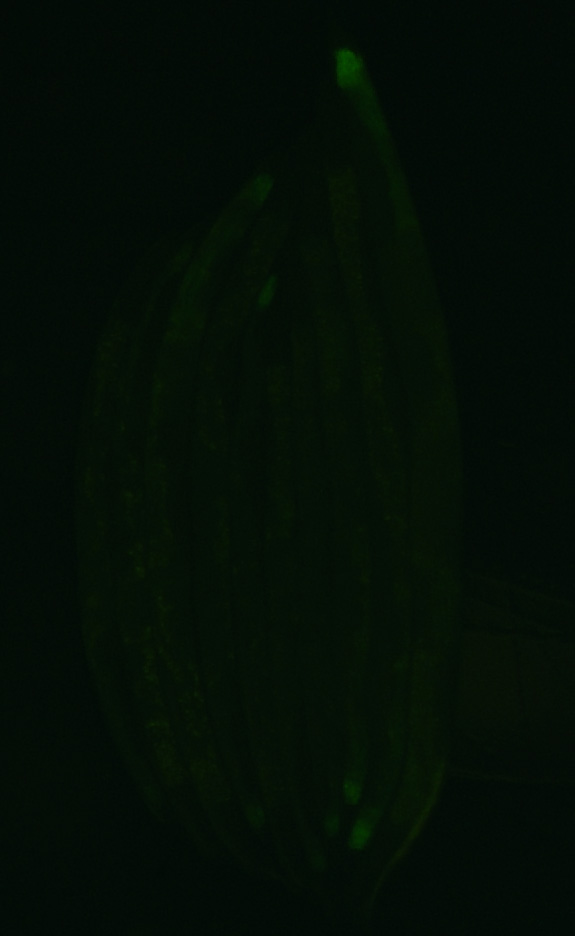

Supplement: Supplementary file 15 — Figure Source Data EV figures [file 44318_2025_634_MOESM15_ESM.zip › EMBOJ-2025-121287-T_SourceDataEVFigures/Figure EV1/Source data_Figure EV1B/ΔyciA.jpg]
