# Supplementary figures and images for: Iron-deplete diet enhances Caenorhabditis elegans lifespan via oxidative stress response pathways (part 2 of 2)
Source: EMBO J. 2025 Nov 10;44(24):7565–89. doi: 10.1038/s44318-025-00634-7 (PMC12706066; doi:10.1038/s44318-025-00634-7)

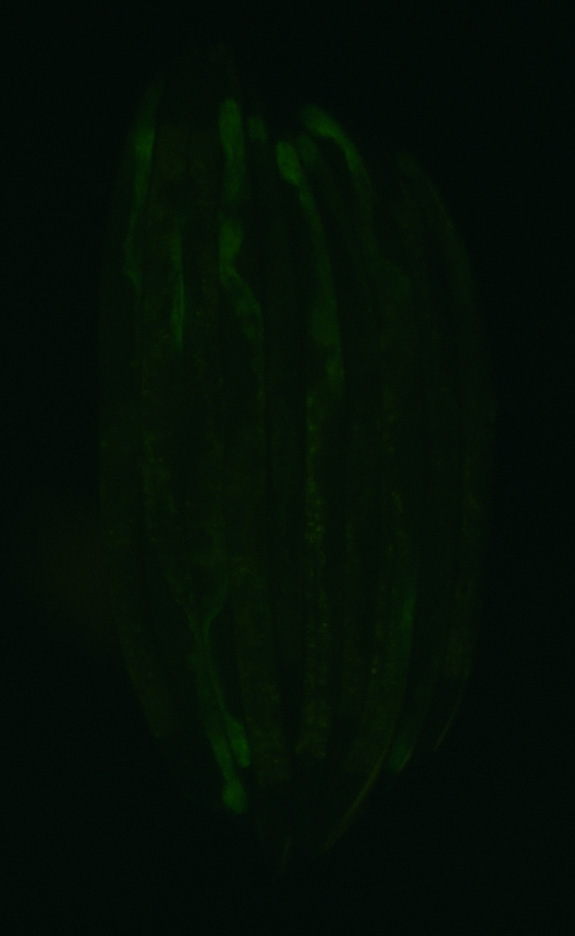

Supplement: Supplementary file 15 — Figure Source Data EV figures [file 44318_2025_634_MOESM15_ESM.zip › EMBOJ-2025-121287-T_SourceDataEVFigures/Figure EV1/Source data_Figure EV1B/ΔyejG.jpg]

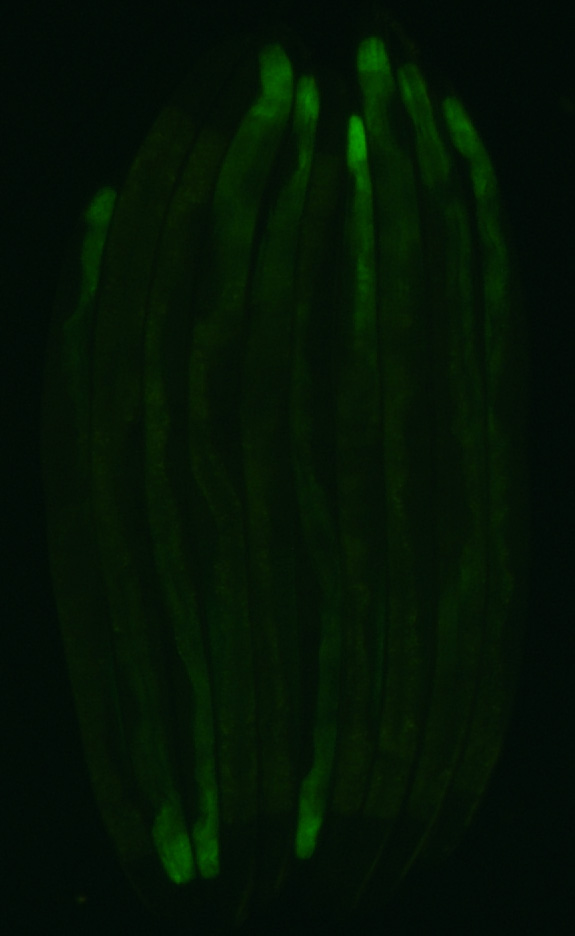

Supplement: Supplementary file 15 — Figure Source Data EV figures [file 44318_2025_634_MOESM15_ESM.zip › EMBOJ-2025-121287-T_SourceDataEVFigures/Figure EV1/Source data_Figure EV1B/ΔyfaT.jpg]

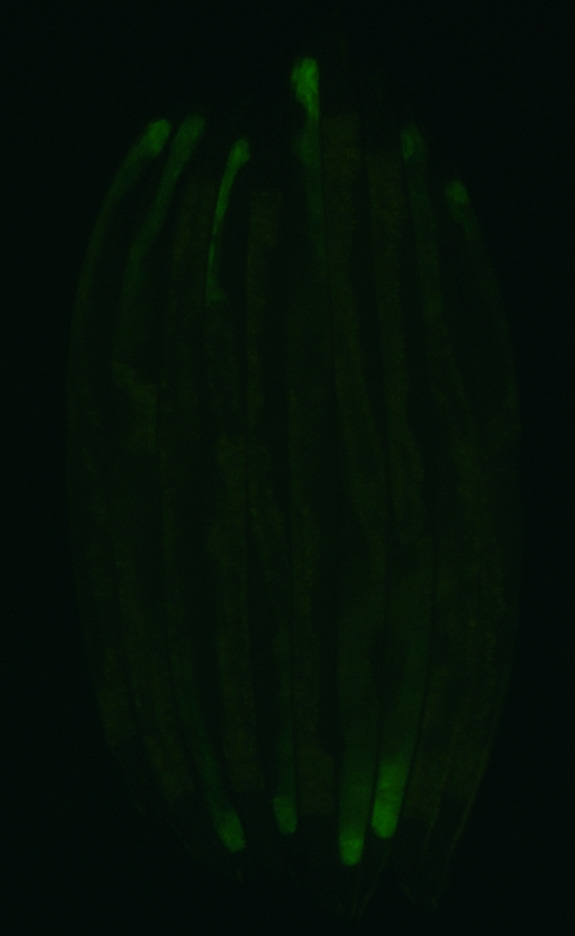

Supplement: Supplementary file 15 — Figure Source Data EV figures [file 44318_2025_634_MOESM15_ESM.zip › EMBOJ-2025-121287-T_SourceDataEVFigures/Figure EV1/Source data_Figure EV1B/Δymfm.jpg]

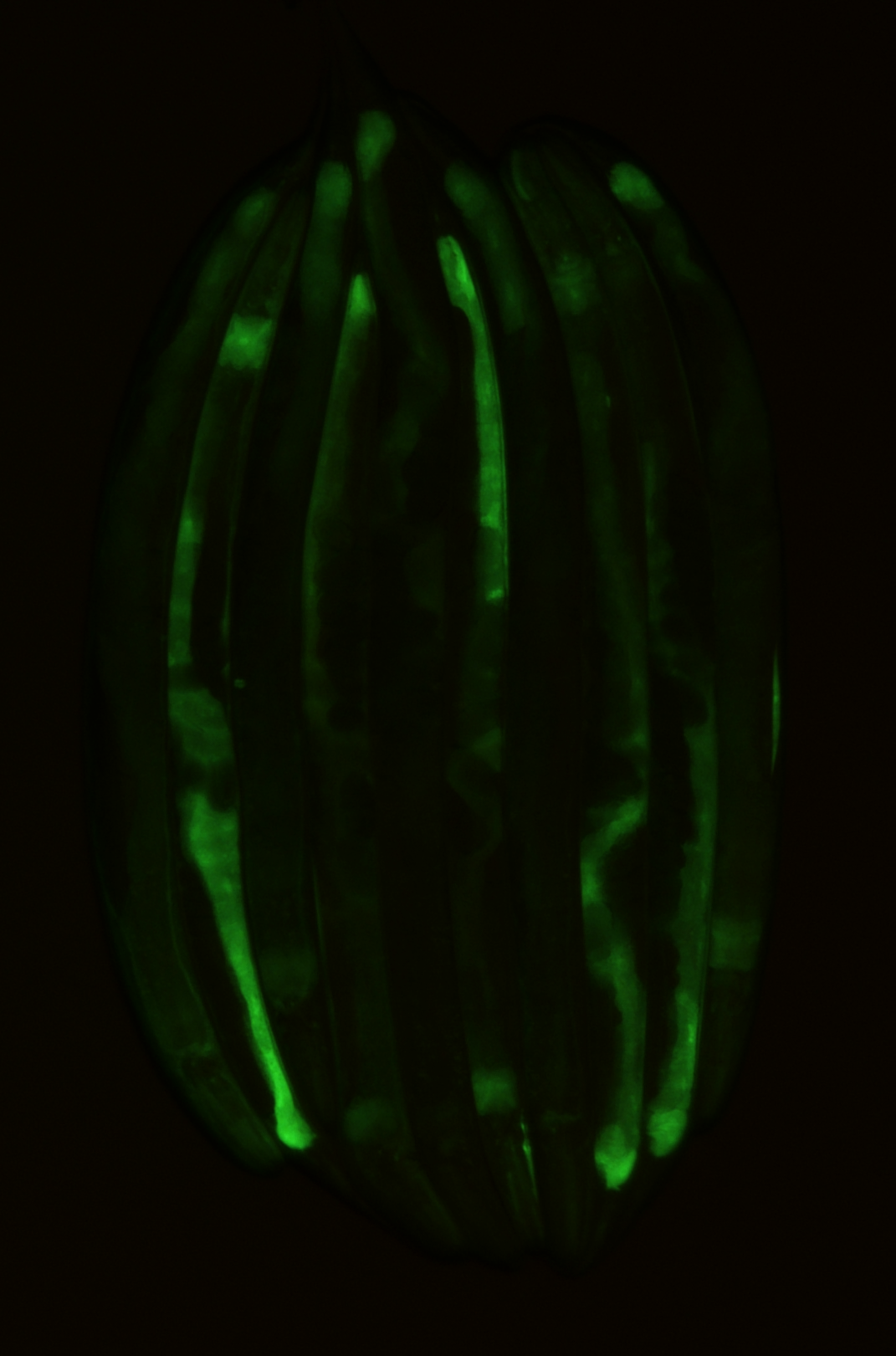

Supplement: Supplementary file 15 — Figure Source Data EV figures [file 44318_2025_634_MOESM15_ESM.zip › EMBOJ-2025-121287-T_SourceDataEVFigures/Figure EV4/Source data_Figure EV4A/5 mM PQ BW25113.tif]

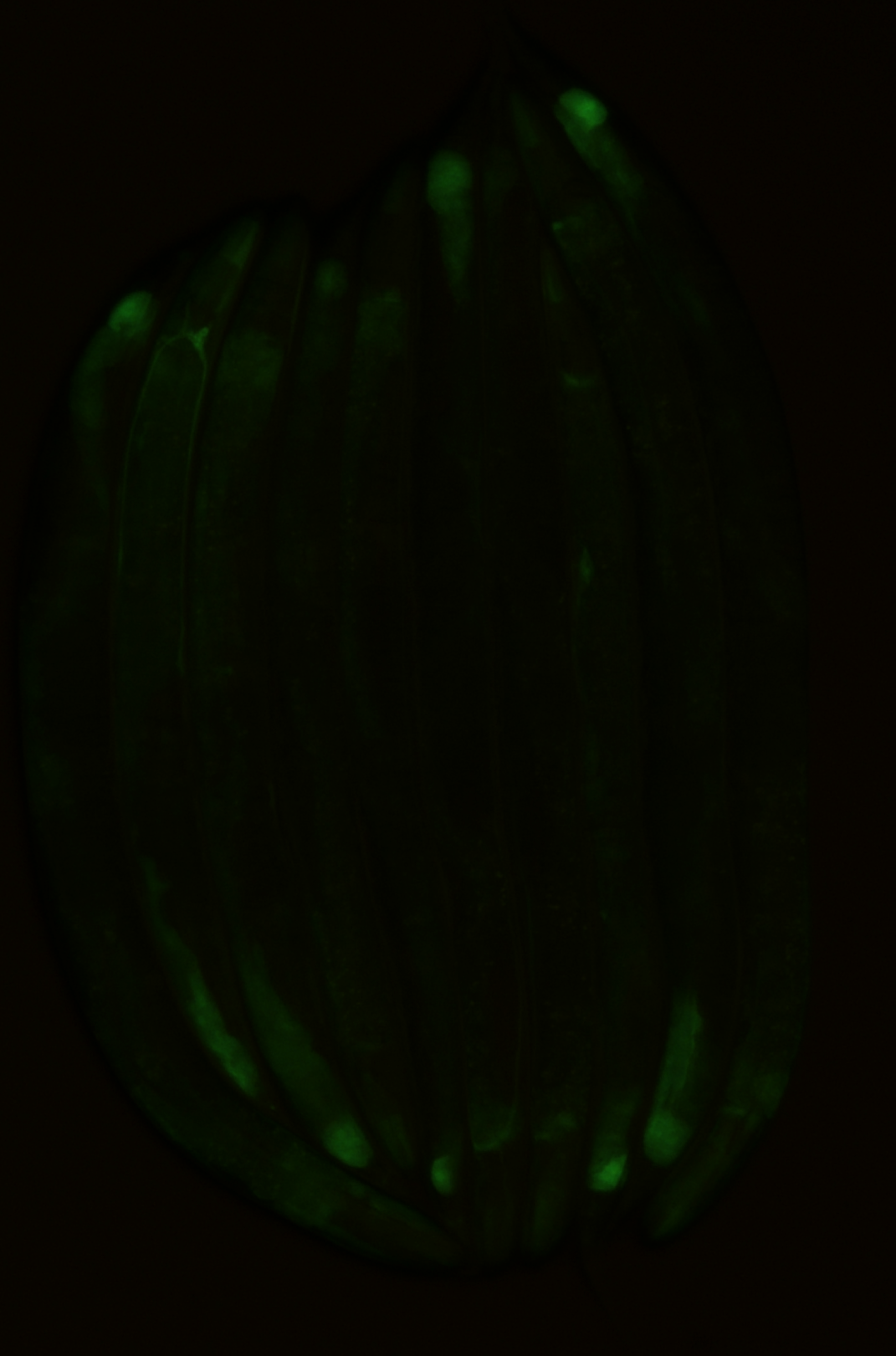

Supplement: Supplementary file 15 — Figure Source Data EV figures [file 44318_2025_634_MOESM15_ESM.zip › EMBOJ-2025-121287-T_SourceDataEVFigures/Figure EV4/Source data_Figure EV4A/BW25113.tif]

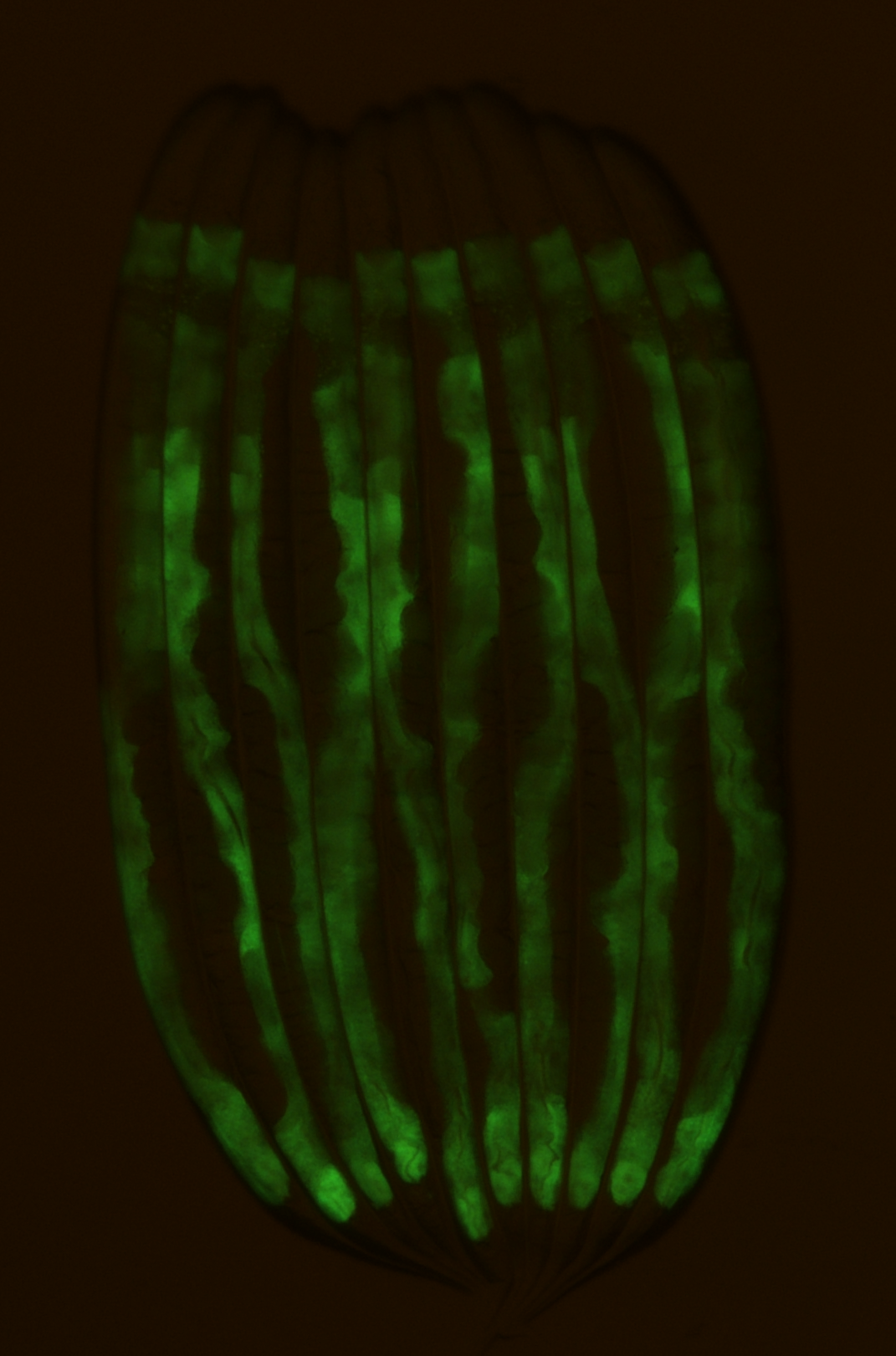

Supplement: Supplementary file 15 — Figure Source Data EV figures [file 44318_2025_634_MOESM15_ESM.zip › EMBOJ-2025-121287-T_SourceDataEVFigures/Figure EV4/Source data_Figure EV4C/5 mM PQ BW25113.tif]

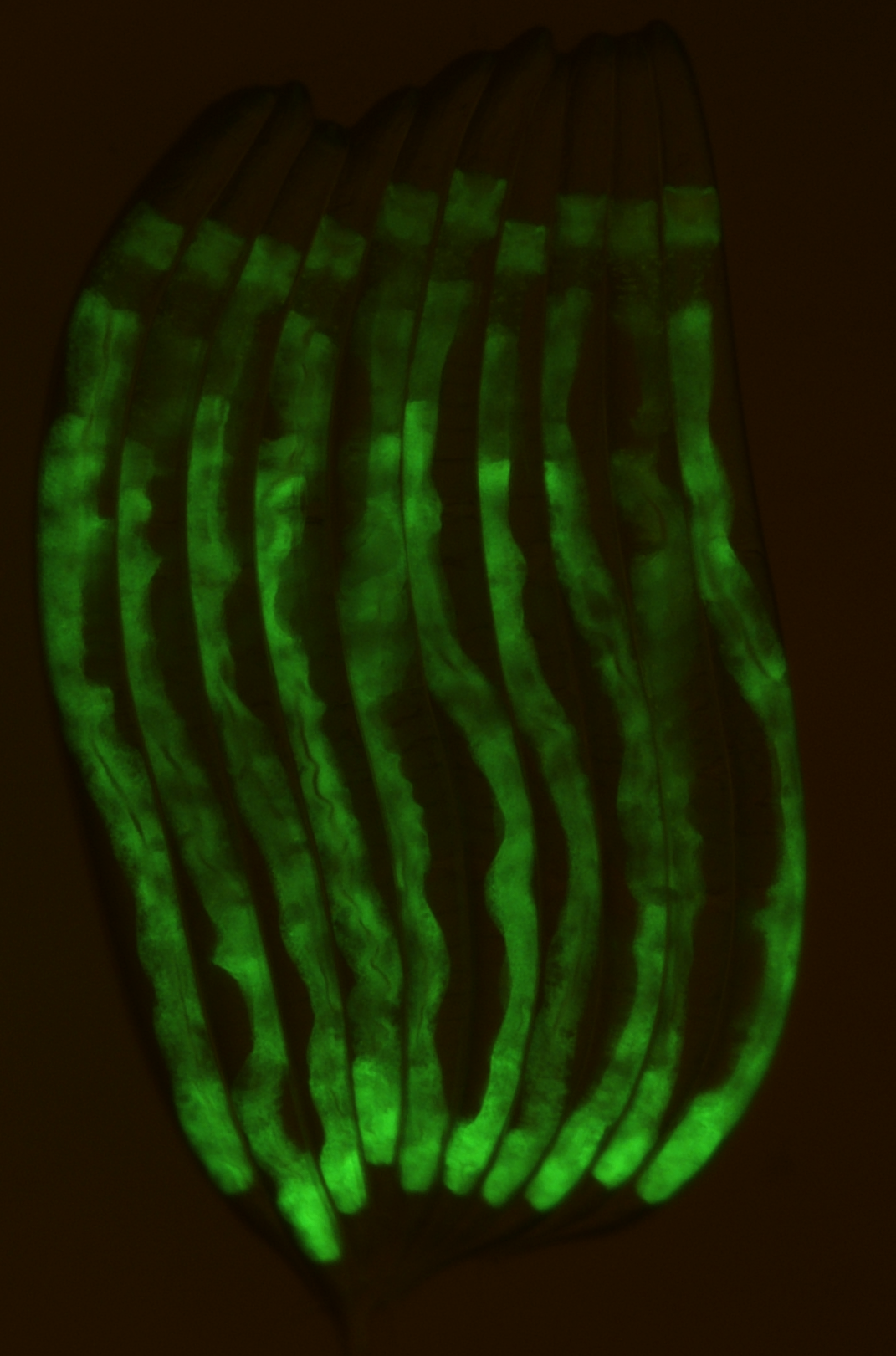

Supplement: Supplementary file 15 — Figure Source Data EV figures [file 44318_2025_634_MOESM15_ESM.zip › EMBOJ-2025-121287-T_SourceDataEVFigures/Figure EV4/Source data_Figure EV4C/BW25113.tif]

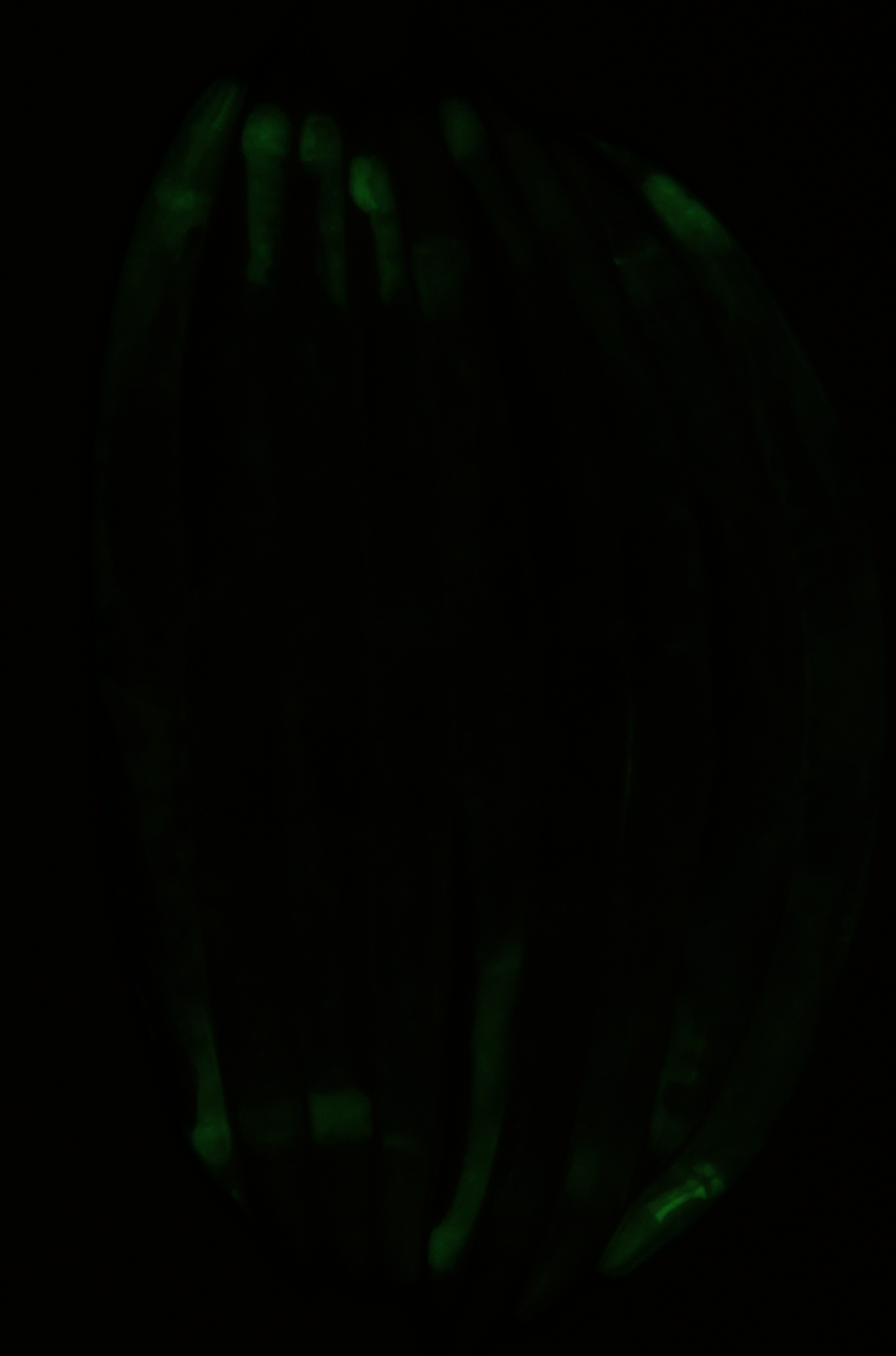

Supplement: Supplementary file 15 — Figure Source Data EV figures [file 44318_2025_634_MOESM15_ESM.zip › EMBOJ-2025-121287-T_SourceDataEVFigures/Figure EV4/Source data_Figure EV4E/EV.tif]

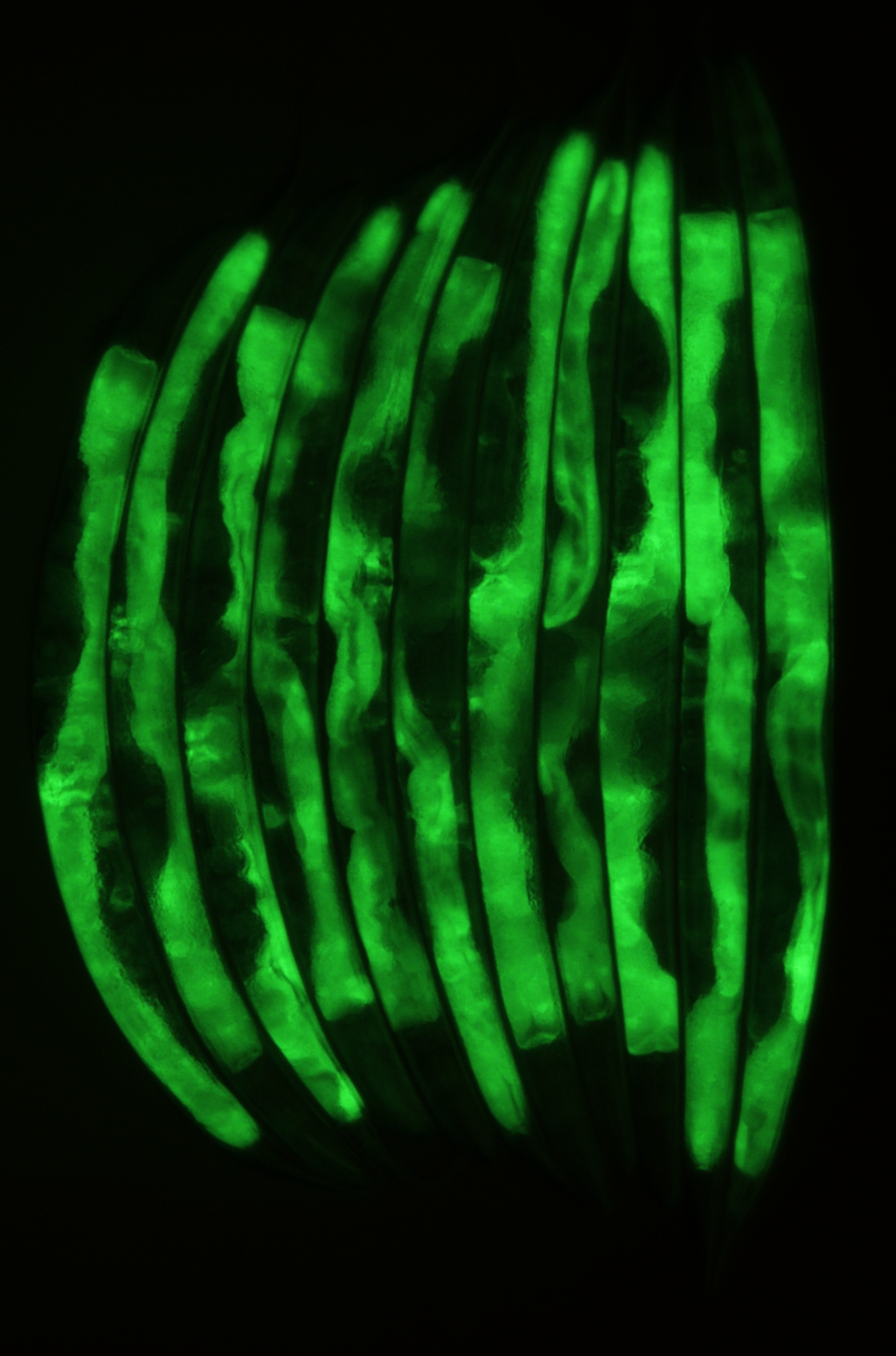

Supplement: Supplementary file 15 — Figure Source Data EV figures [file 44318_2025_634_MOESM15_ESM.zip › EMBOJ-2025-121287-T_SourceDataEVFigures/Figure EV4/Source data_Figure EV4E/tomm-22.tif]

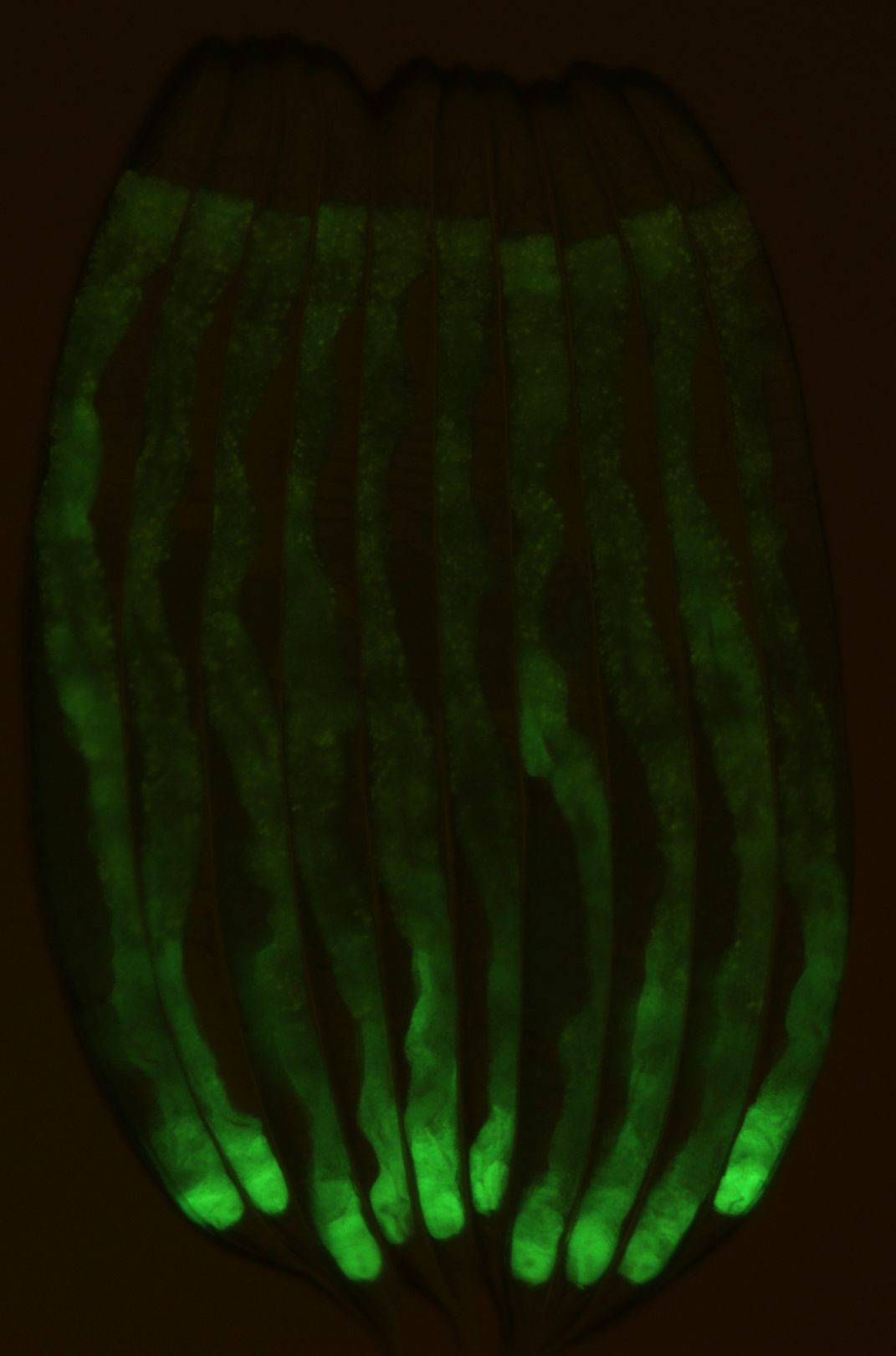

Supplement: Supplementary file 15 — Figure Source Data EV figures [file 44318_2025_634_MOESM15_ESM.zip › EMBOJ-2025-121287-T_SourceDataEVFigures/Figure EV4/Source data_Figure EV4G/EV.tif]

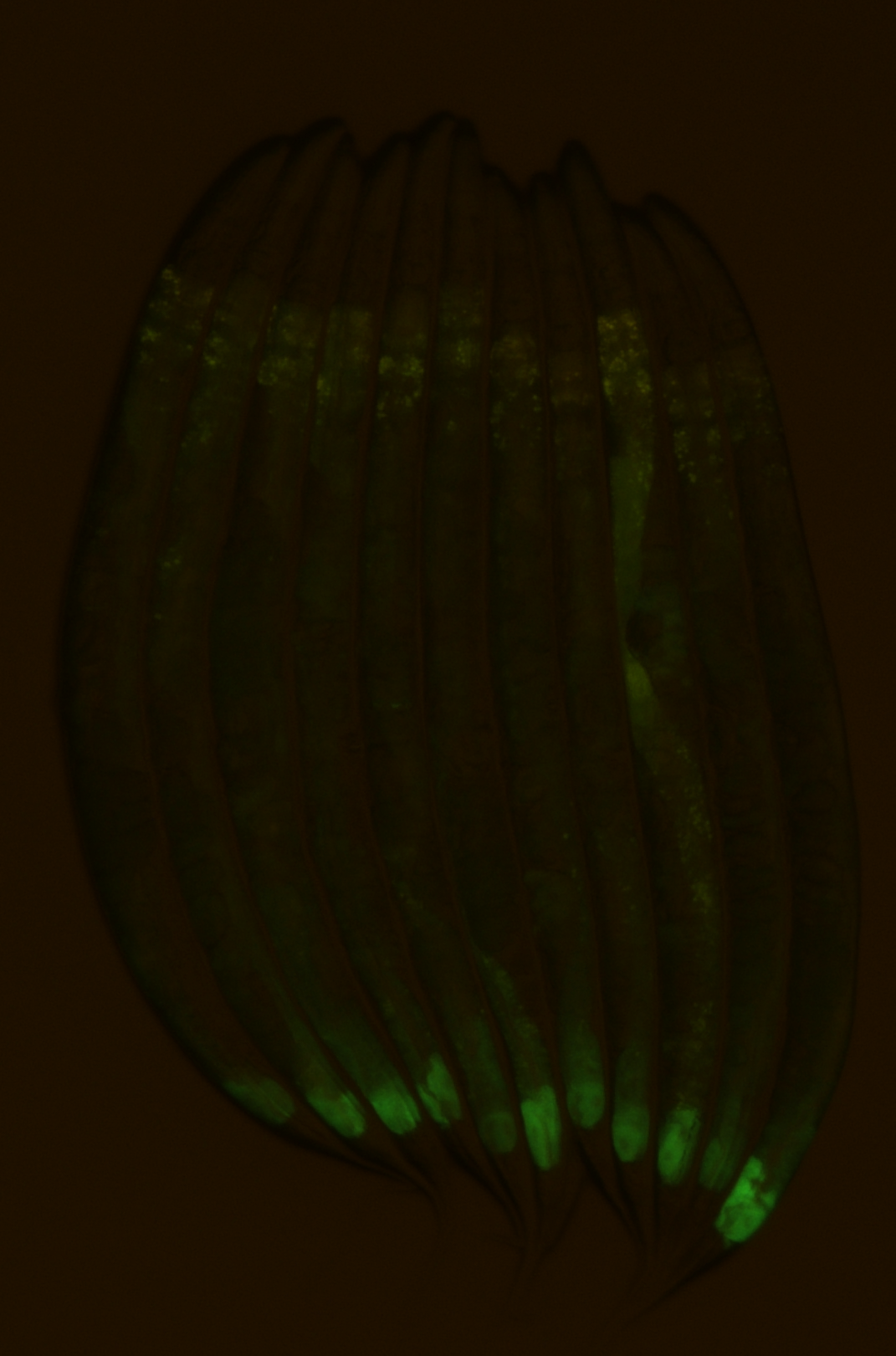

Supplement: Supplementary file 15 — Figure Source Data EV figures [file 44318_2025_634_MOESM15_ESM.zip › EMBOJ-2025-121287-T_SourceDataEVFigures/Figure EV4/Source data_Figure EV4G/tomm-22.tif]

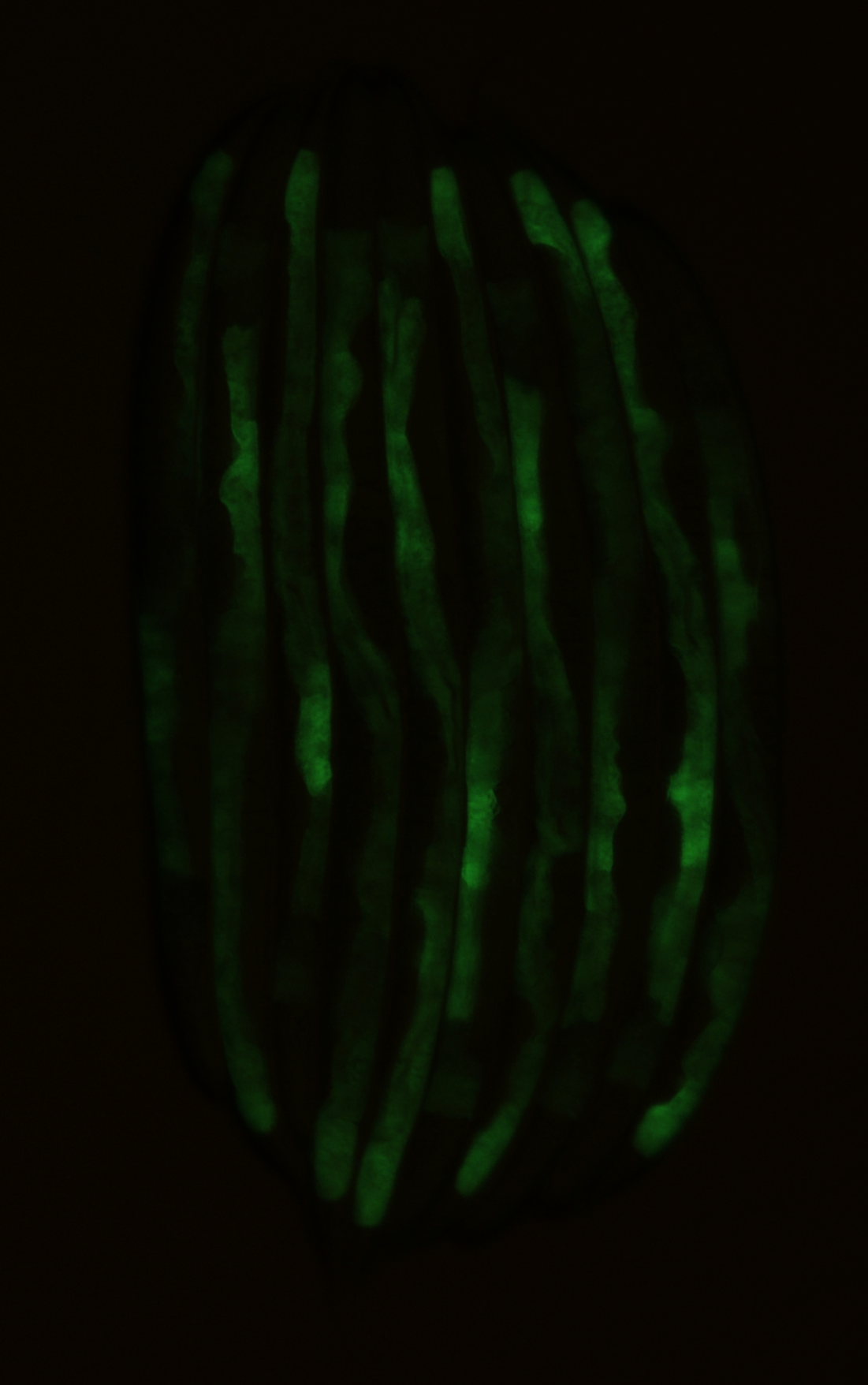

Supplement: Supplementary file 15 — Figure Source Data EV figures [file 44318_2025_634_MOESM15_ESM.zip › EMBOJ-2025-121287-T_SourceDataEVFigures/Figure EV5/Source data_Figure EV5B/eat-2;fat-7 bw.tif]

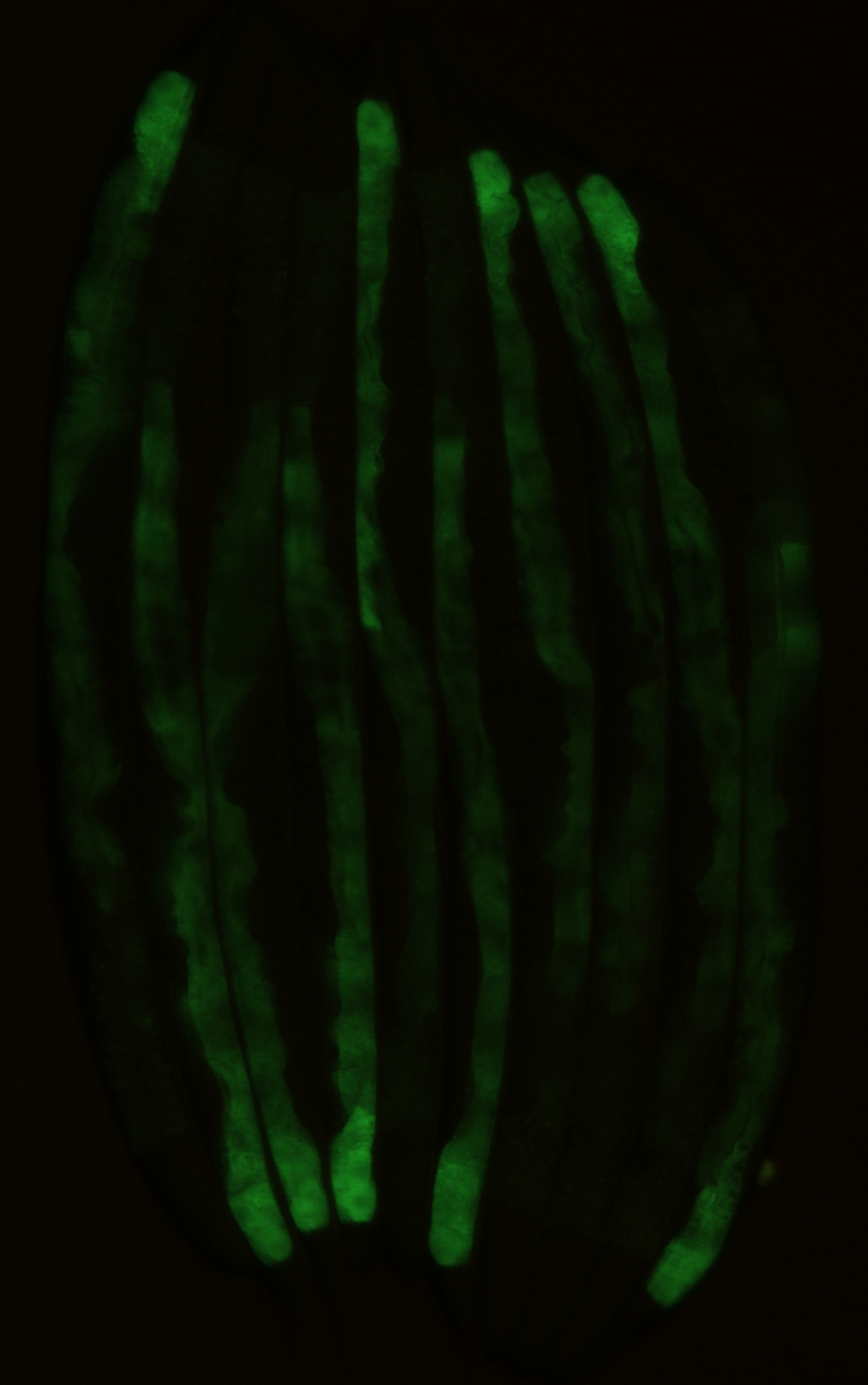

Supplement: Supplementary file 15 — Figure Source Data EV figures [file 44318_2025_634_MOESM15_ESM.zip › EMBOJ-2025-121287-T_SourceDataEVFigures/Figure EV5/Source data_Figure EV5B/fat-7 bw.tif]

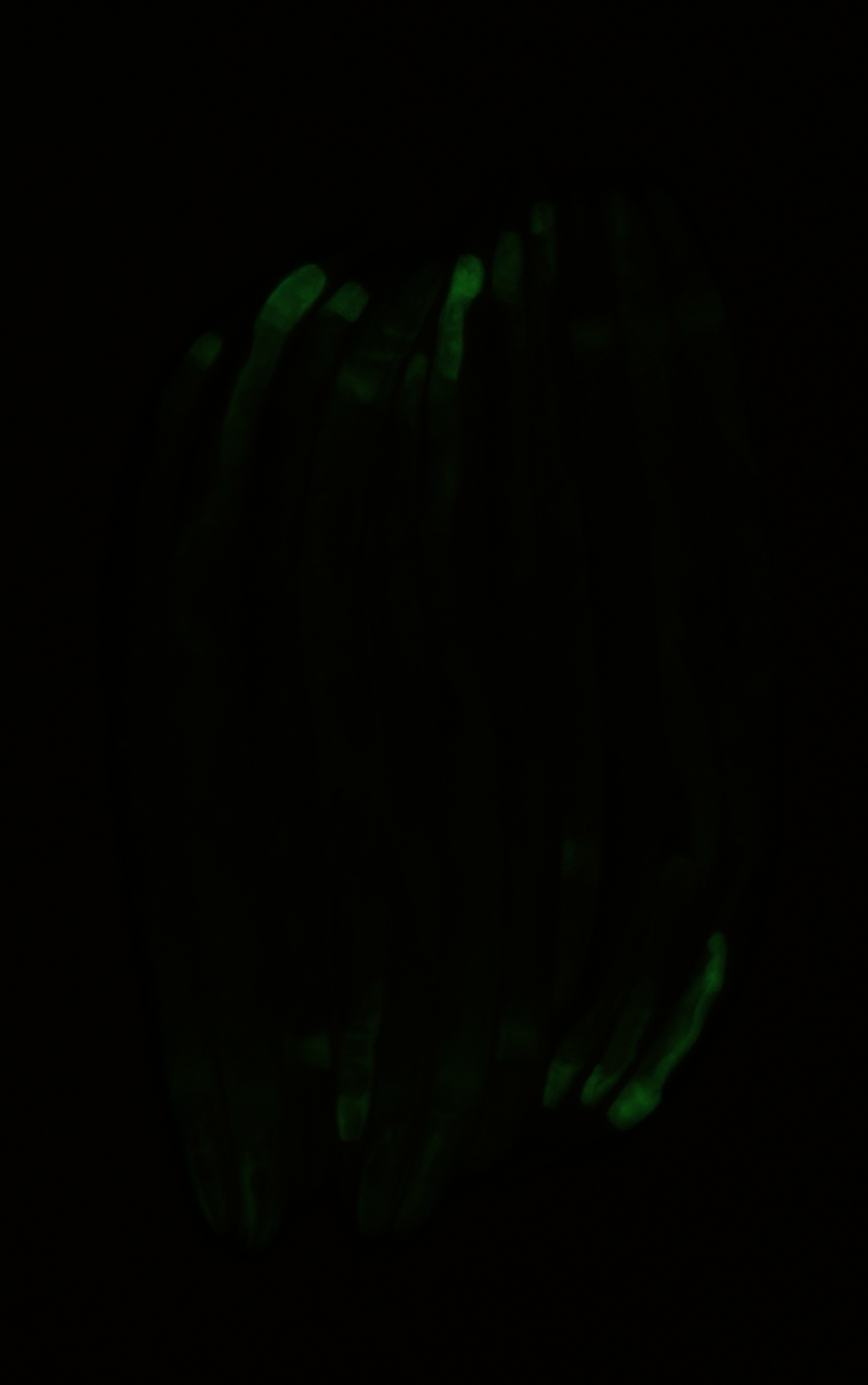

Supplement: Supplementary file 15 — Figure Source Data EV figures [file 44318_2025_634_MOESM15_ESM.zip › EMBOJ-2025-121287-T_SourceDataEVFigures/Figure EV5/Source data_Figure EV5D/eat-2;hsp-6.tif]

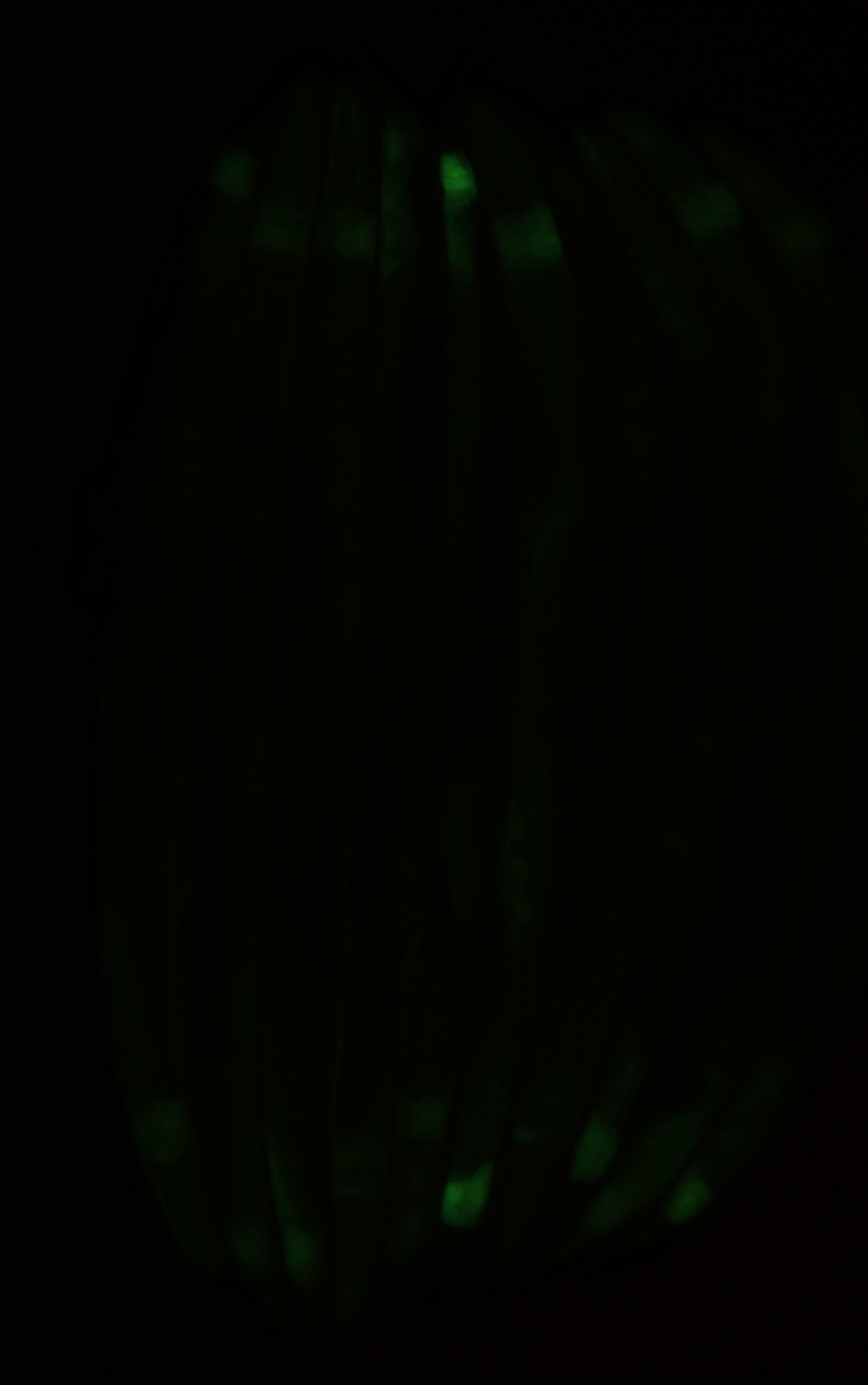

Supplement: Supplementary file 15 — Figure Source Data EV figures [file 44318_2025_634_MOESM15_ESM.zip › EMBOJ-2025-121287-T_SourceDataEVFigures/Figure EV5/Source data_Figure EV5D/hsp-6.tif]
