# Supplementary material for: Iron-deplete diet enhances Caenorhabditis elegans lifespan via oxidative stress response pathways
Source: EMBO J. 2025 Nov 10;44(24):7565–89. doi: 10.1038/s44318-025-00634-7 (PMC12706066; doi:10.1038/s44318-025-00634-7)
Supplement: Supplementary file 16 — Expanded View Figures [file 44318_2025_634_MOESM16_ESM.pdf]

Expanded View Figures

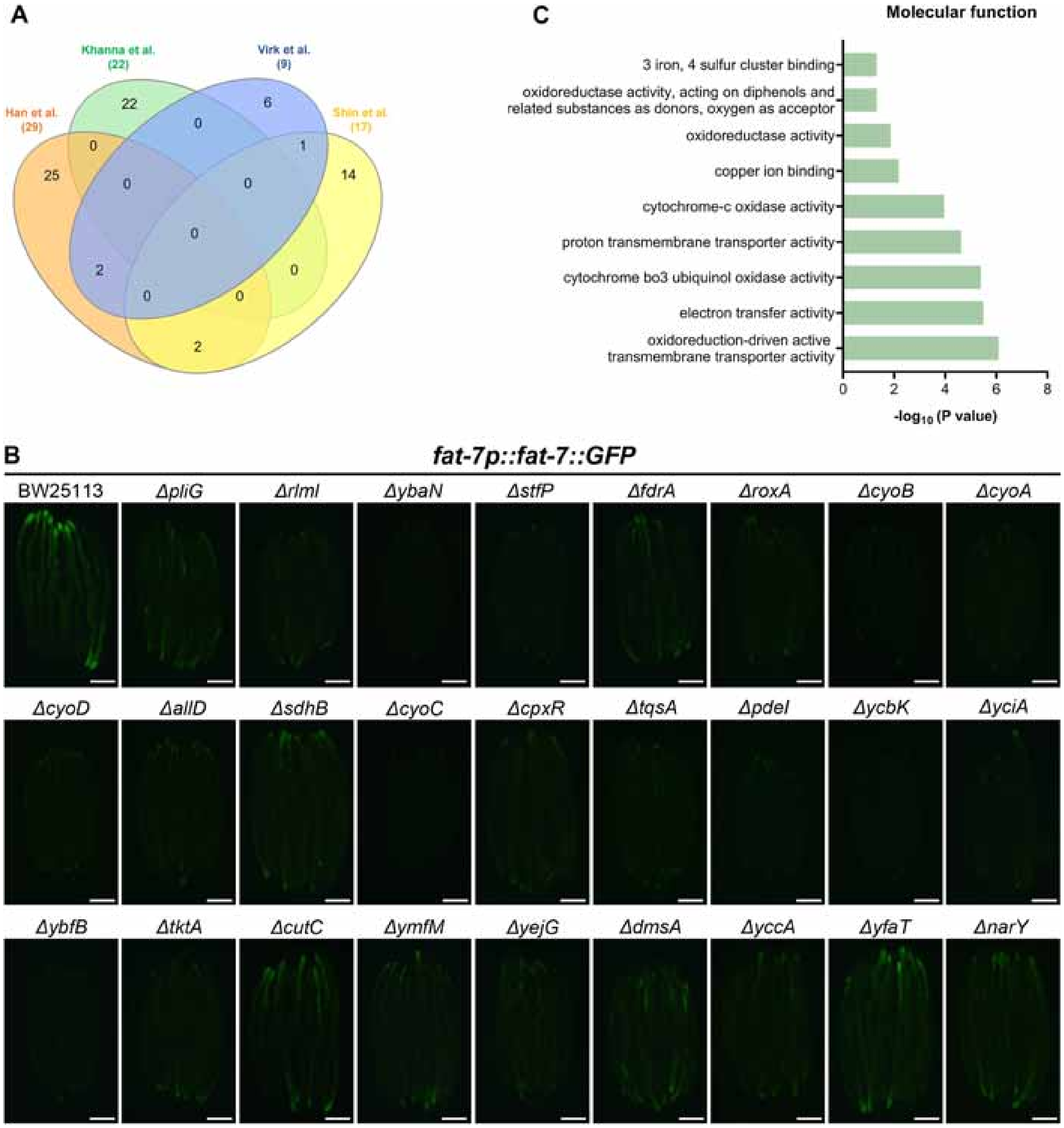

Figure EV1. Genome-wide bacterial screen identifies *E. coli* mutants that modulate *C. elegans* FAT-7 levels.

(**A**) Venn diagram showing the overlap among *E. coli* mutants identified in genome-wide bacterial screens for mutants that extend *C. elegans* lifespan (Han et al, 2017a; Khanna et al, 2016; Shin et al, 2020; Virk et al, 2016). (**B**) Representative fluorescence images of *fat-7p::fat-7::GFP* worms grown on *E. coli* BW25113 and mutant diets. Scale bar = 200 µm. (**C**) Gene ontology enrichment analysis for molecular functions associated with the 26 *E. coli* mutants that downregulate *C. elegans* FAT-7 levels. The statistical analysis was performed using Fisher's exact test. Source data are available online for this figure.

© The Author(s)

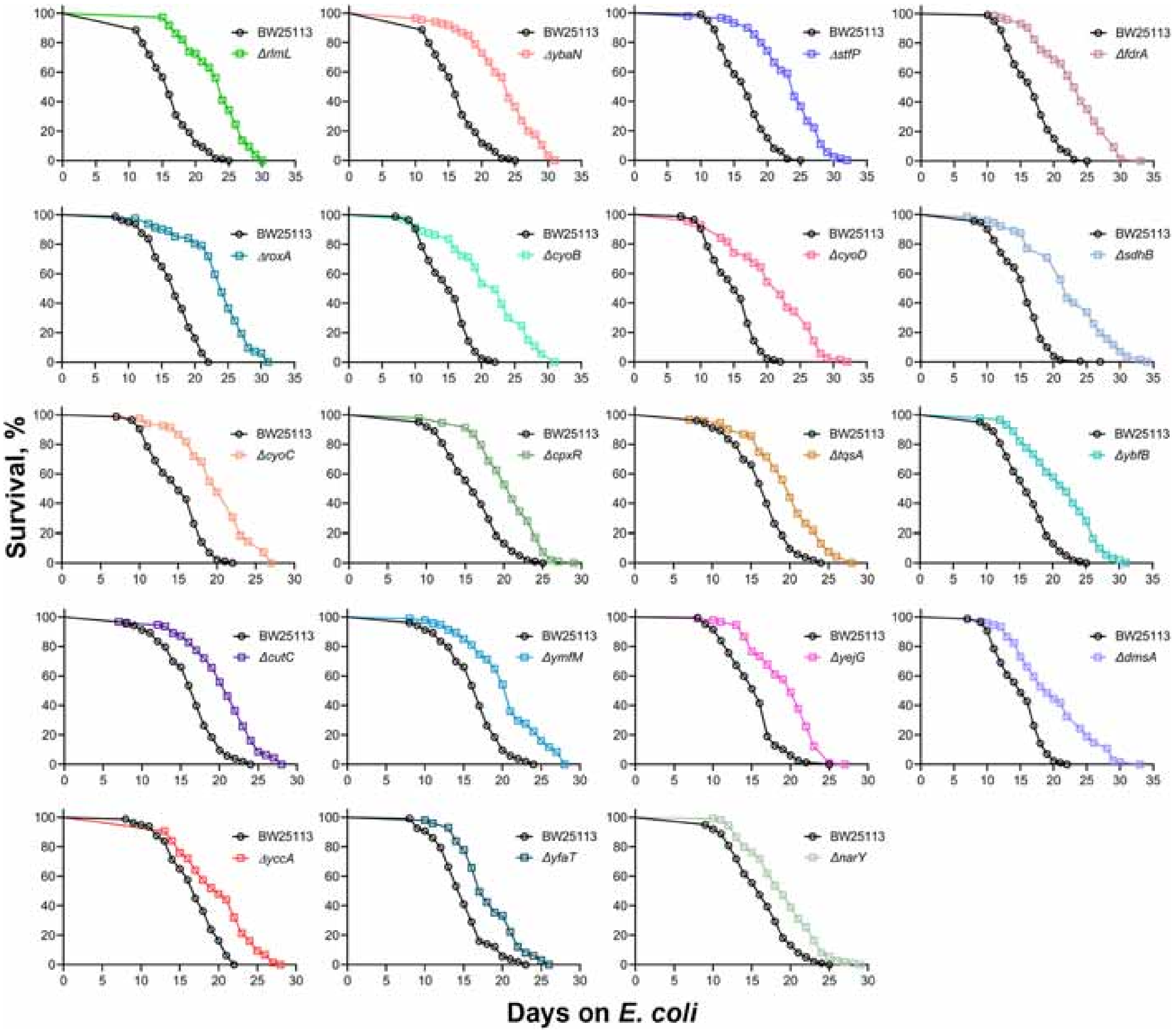

Figure EV2. ***E. coli*** **mutants that decrease FAT-7 levels extend** ***C. elegans*** **lifespan.**

Representative survival curves of N2 worms fed on *E. coli* BW25113 and mutants that decrease FAT-7 levels. The BW25113 control is common for different cohorts of the survival curves. The survival curves for the remaining mutants are shown in Figs. 2A–C and 3A. Source data are available online for this figure.

 © The Author(s)

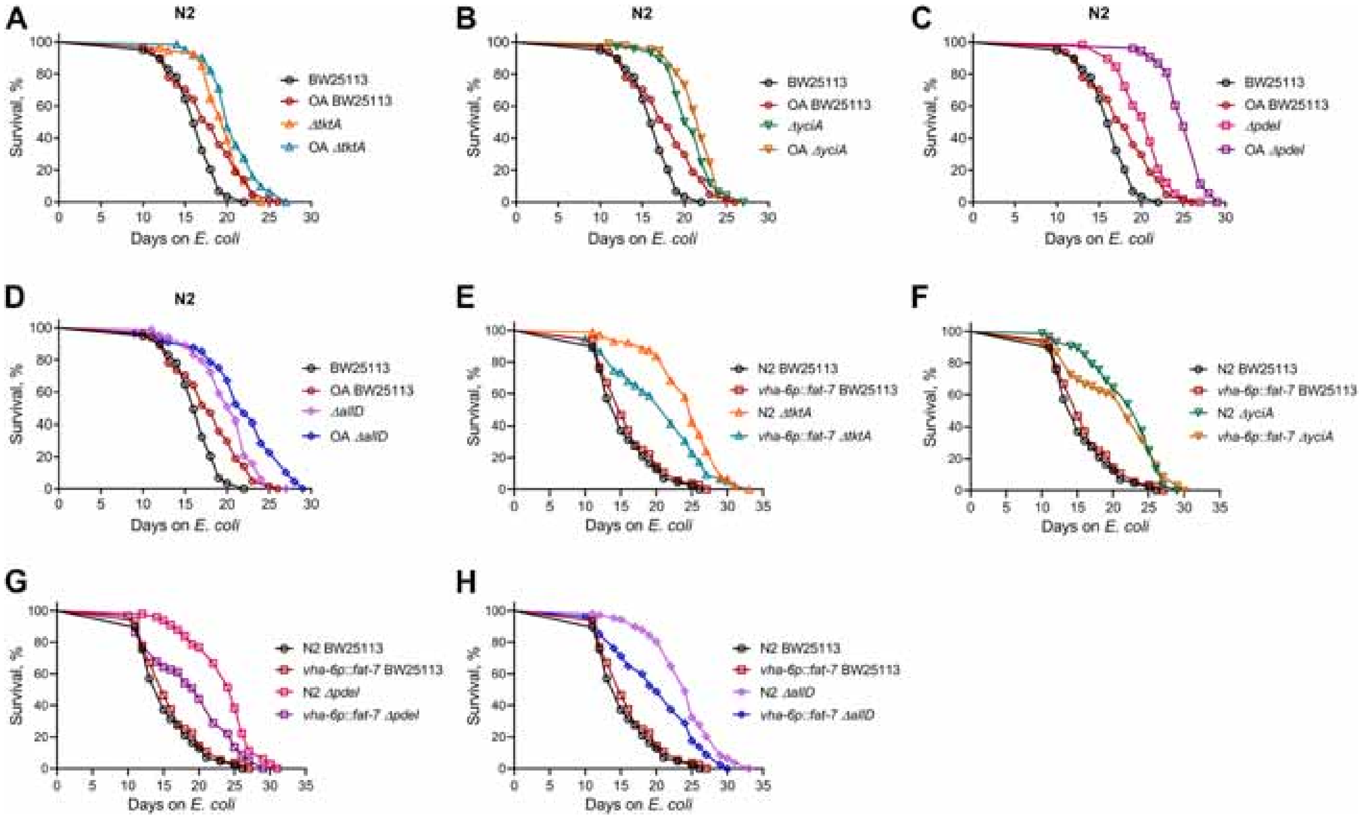

Figure EV3. ***E. coli*** **mutants do not extend** ***C. elegans*** **lifespan via oleic acid.**

(A–D) Representative survival curves of N2 worms fed on *E. coli* BW25113 with or without 2 mM oleic acid (OA), along with N2 worms fed on *ΔtktA* (**A**), *ΔyciA* (**B**), *Δpdel* (**C**), and *ΔallD* (**D**) *E. coli* mutants with or without 2 mM OA. The BW25113 control is common for all the panels. $P < 0.001$ for *ΔtktA, ΔyciA, Δpdel*, and *ΔallD* compared to their respective control BW25113 ($n = 3$ biological replicates; animals per condition per replicate >52). The hazard ratios and P values calculated by Cox regression analysis on each of the mutant diets for their interaction with oleic acid supplementation are the following: *tktA*:OA (hazard ratio = 1.629, $P = 0.0669$), *yciA*:OA (hazard ratio = 2.356, $P = 0.0006$), *pdel*:OA (hazard ratio = 0.4756, $P = 0.0094$) and *allD*:OA (hazard ratio = 1.096, $P = 0.711$). **(E–H)** Representative survival curves of N2 and *vha-6p::fat-7* worms grown on *E. coli* BW25113, along with N2 and *vha-6p::fat-7* worms grown on *ΔtktA* (**E**), *ΔyciA* (**F**), *Δpdel* (**G**), and *ΔallD* (**H**) *E. coli* mutants. The BW25113 control is common for all the panels. $P < 0.001$ for *ΔtktA, ΔyciA, Δpdel*, and *ΔallD* compared to their respective control BW25113 ($n = 3$ biological replicates; animals per condition per replicate >49). Source data are available online for this figure.

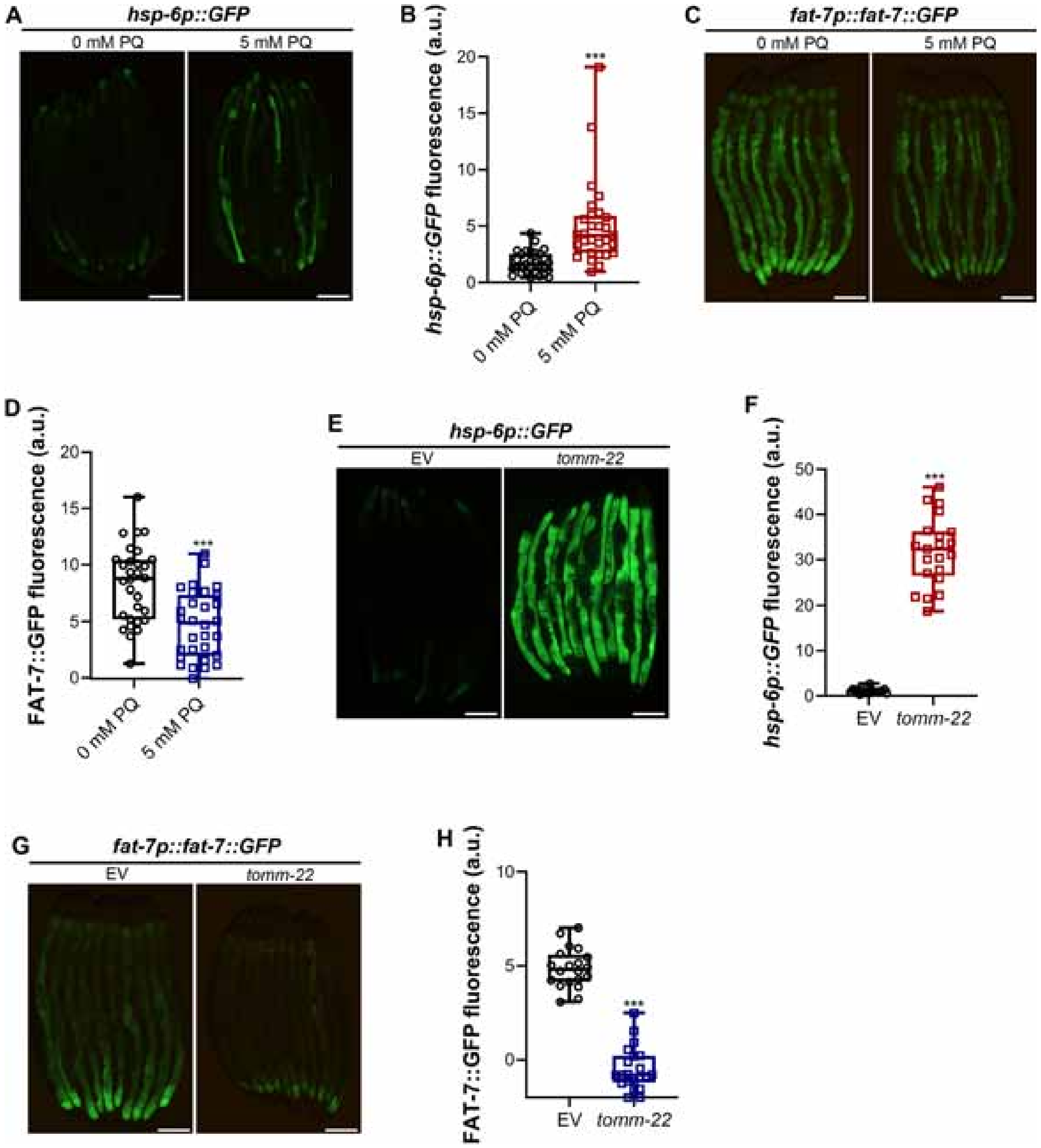

Figure EV4. Mitochondrial stress suppresses *fat-7* expression.

(**A**) Representative fluorescence images of *hsp-6p::GFP* worms exposed to *E. coli* BW25113 supplemented with 0 or 5 mM paraquat dichloride (PQ) for 24 h. Scale bar = 200 µm. (**B**) Quantification of GFP levels of *hsp-6p::GFP* worms exposed to *E. coli* BW25113 supplemented with 0 or 5 mM PQ for 24 h. ***$P < 0.0001$ via the *t* test ($n = 29$–30 worms each). (**C**) Representative fluorescence images of *fat-7p::fat-7::GFP* worms grown on *E. coli* BW25113 supplemented with 0 or 5 mM PQ for 24 h. Scale bar = 200 µm. (**D**) Quantification of GFP levels of *fat-7p::fat-7::GFP* worms grown on *E. coli* BW25113 supplemented with 0 or 5 mM PQ for 24 h. ***$P < 0.0001$ via the *t* test ($n = 30$ worms each). (**E**) Representative fluorescence images of *hsp-6p::GFP* worms grown on empty vector (EV) control or *tomm-22* RNAi. Scale bar = 200 µm. (**F**) Quantification of GFP levels of *hsp-6p::GFP* worms grown on EV control or *tomm-22* RNAi. ***$p < 0.0001$ via the *t* test ($n = 19$–21 worms each). (**G**) Representative fluorescence images of *fat-7p::fat-7::GFP* worms grown on EV control or *tomm-22* RNAi. Scale bar = 200 µm. (**H**) Quantification of GFP levels of *fat-7p::fat-7::GFP* worms grown on EV control or *tomm-22* RNAi. ***$p < 0.0001$ via the *t* test ($n = 20$–21 worms each). Data information: In the boxplots in (**B**, **D**, **F**, **H**), the central bands represent the median value, the boxes represent the upper and lower quartiles, and the whiskers represent the minimum and maximum values. Source data are available online for this figure.

 © The Author(s)

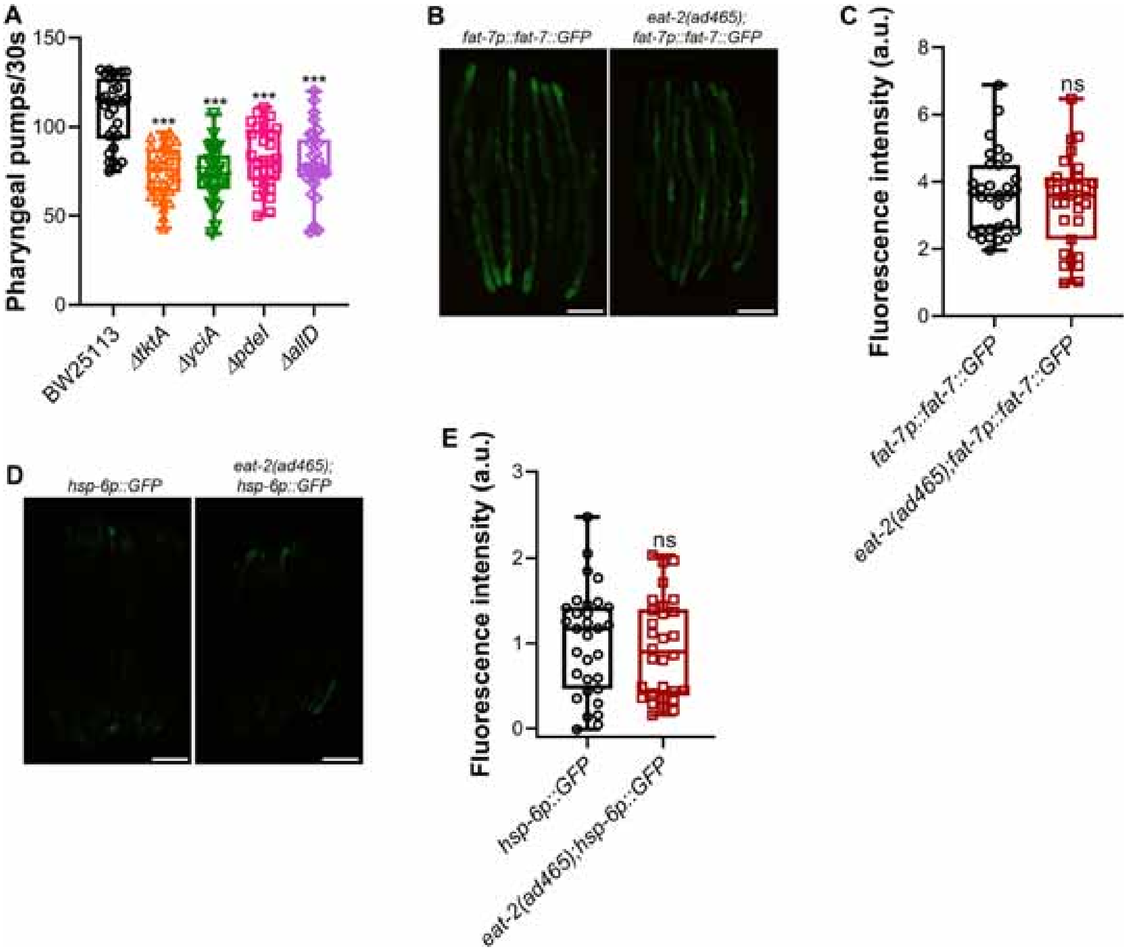

Figure EV5. Reduced pharyngeal pumping does not cause mitochondrial stress.

(**A**) Pharyngeal pumps per 30 s of 1-day-old adult N2 animals grown on *E. coli* BW25113 and FAT-7-suppressing diets at 20 °C. ***$P < 0.0001$ via the *t* test ($n = 30$ worms each). (**B**) Representative fluorescence images of *fat-7p::fat-7::GFP* and *eat-2(ad465);fat-7p::fat-7::GFP* worms grown on *E. coli* BW25113. Scale bar = 200 µm. (**C**) Quantification of GFP levels of *fat-7p::fat-7::GFP* and *eat-2(ad465);fat-7p::fat-7::GFP* worms grown on *E. coli* BW25113. $P = 0.4056$, ns, nonsignificant via the *t* test ($n = 31$ worms each). (**D**) Representative fluorescence images of *hsp-6p::GFP* and *eat-2(ad465);hsp-6p::GFP* worms grown on *E. coli* BW25113. Scale bar = 200 µm. (**E**) Quantification of GFP levels of *hsp-6p::GFP* and *eat-2(ad465);hsp-6p::*GFP worms grown on *E. coli* BW25113. $p = 0.6479$, ns, nonsignificant via the *t* test ($n = 30–31$ worms each). Data information: In the boxplots in (**A**, **C**, **E**), the central bands represent the median value, the boxes represent the upper and lower quartiles, and the whiskers represent the minimum and maximum values. Source data are available online for this figure.
